# Social Functioning Interventions in Psychosis: A Systematic Review

**DOI:** 10.1002/cpp.70090

**Published:** 2025-05-29

**Authors:** M. Vinu, A. Georgiades

**Affiliations:** ^1^ Department of Psychosis Studies, Institute of Psychiatry, Psychology, and Neuroscience (IoPPN) King's College London London UK; ^2^ Brent Early Intervention Service CNWL NHS Foundation Trust London UK

**Keywords:** cognitive model, interventions, psychosis, schizophrenia, social functioning

## Abstract

**Objective:**

A decline in social functioning is a hallmark of psychosis and is evident across the psychosis continuum. However, no study to date has summarised the existing evidence base regarding social functioning interventions in psychosis, nor have they synthesised the factors associated with high or low social functioning in psychosis.

**Method:**

A systematic review was conducted to summarise the extant literature regarding social functioning interventions in psychosis.

**Results:**

Sixty‐five studies were eligible for inclusion. Physical exercise, art therapy, social recovery therapy, social skills training, virtual reality, online programmes and psychosocial interventions improved social functioning and reduced both positive and negative symptoms of psychosis. Factors associated with low social functioning in psychosis included *self‐perception* (self‐esteem, self‐efficacy, internalised stigma), *symptoms* (social anxiety, depression, positive and negative symptoms), *emotion* (reduced emotional awareness/regulation, emotional suppression, negative affect), *cognition* (appraisals, negative self‐beliefs, dependency and enmeshment schema, negative self‐statements, defeatist performance beliefs, metacognitive beliefs), *social cognition* (ToM, neurocognition) and *behaviours* (motivation, social relatedness, avoidance). Factors associated with high social functioning in psychosis included *emotional awareness*, *acceptance of emotions*, *positive affect*, *cognitive reappraisal*, *positive performance beliefs* and *adaptive coping*.

**Conclusions:**

A number of factors were associated with high or low social functioning in psychosis, which highlights important clinical intervention targets for devising novel social functioning interventions. The *cognitive model of social functioning in psychosis* could facilitate the development of personalised and idiosyncratic formulations and targeted interventions in CBTp to enhance social functioning in psychosis.

**Summary**

- *Negative factors contributing to poor social functioning in psychosis* include self‐perception, symptoms, emotion, cognition, social cognition and behaviours.
- *Positive factors contributing to social functioning in psychosis* include increasing emotional awareness, acceptance of emotions, positive affect, cognitive reappraisals, positive performance beliefs and adaptive coping. This therefore highlights a strengths‐based approach to improving social functioning in psychosis.
- No social functioning interventions to date have explicitly incorporated both sets of positive and negative factors within their intervention protocols, highlighting an avenue for future research.
- The *cognitive model of social functioning in psychosis* enables the development of idiosyncratic case formulations regarding social functioning impairments, which would enhance current CBTp practices.

## 1 Introduction

Social functioning has been defined as an individual's interactions with their environment and their ability to fulfil their role in work, social activities and relationships (Bosc 2000). A decline in social functioning is a hallmark of psychosis and often emerges before illness onset and typically develops into long‐standing difficulties (Addington et al. 2008; Carrión et al. 2013; Robinson et al. 2004). A significant decline in social functioning has been found across all stages of psychosis, from clinical high risk (CHR), and first episode of psychosis (FEP) to chronic schizophrenia (SZ) (Abel and Minor 2021; Addington et al. 2008; Raghavan et al. 2017), and across life domains such as work, societal roles and relationships (de Winter et al. 2022). Impairments in social functioning have been associated with positive symptoms of psychosis, increased levels of risk and self‐harm and increased risk of relapse, as well as a delayed return to employment and re‐engagement with previously held social roles (Bae et al. 2010; Bergé et al. 2016; Carrión et al. 2021; Kim et al. 2019; Oorschot et al. 2012; Velthorst et al. 2017). Long‐term social functioning deficits have also been associated with an increased severity of negative symptoms such as social withdrawal, avolition and apathy (Kirkpatrick et al. 2006; Marder and Galderisi 2017). The negative impact of protracted social disablement following from an episode of psychosis therefore highlights the clinical relevance of understanding the factors that contribute to and/or maintain low levels of social functioning. The identification of cognitive, emotional and behavioural factors linked to poor social functioning in psychosis would facilitate the development of targeted interventions. Indeed, cognitive factors that have been associated with poor social functioning include social cognition (Addington et al. 2006), reappraisal (Henry et al. 2008) and schema (Taylor and Harper 2017); affective factors include emotional awareness (Kimhy et al. 2016), negative affect (Grove et al. 2016) and emotional suppression (Perry et al. 2011); and behavioural factors include coping strategies (Jalbrzikowski et al. 2014). A comparison of factors found to be associated with poor social functioning with existing interventions designed to improve social functioning would foster innovations in targeted interventions for psychosis. Indeed, increasing interest is growing for the development of interventions explicitly designed to improve social functioning in psychosis (Brissos et al. 2011). However, the substantial heterogeneity in social functioning interventions, the variability in what they explicitly target and the wide range of factors associated with social functioning impairments emphasises the need to synthesise the available literature. This would inform innovative interventions to improve social functioning in psychosis and would facilitate the development of a cognitive model to devise personalised formulations in cognitive behavioural therapy for psychosis (CBTp), thereby supporting the functional recovery of individuals with psychosis.

### 1.1 Aims of the Study

The aim of this systematic review is to provide a comprehensive summary of the existing literature regarding interventions designed to improve social functioning in psychosis, along with the identification of factors associated with high or low social functioning in psychosis, with a view to proposing a cognitive model of social functioning in psychosis.

## 2 Methods

This review was conducted in accordance with the Preferred Reporting Items for Systematic Reviews and Meta‐Analyses (PRISMA) guidelines (Page et al. 2021). Methods and inclusion criteria were specified in advance and documented in a protocol registered with the PROSPERO International Prospective Register of Systematic Reviews (registration number: CRD42024552882).

### 2.1 Search Strategy

A systematic database search of MEDLINE (including PubMed), EMBASE, Global Health and APA PsycINFO was conducted using an OVID search tool from database conception to 30 June 2024. The following search strings were used: Psychosis OR Psychotic OR Schizophreni* **AND** Social function* OR Social recovery **AND** Intervention OR Cognitive behavi* therap* OR CBT OR Therapy.

### 2.2 Study Selection

**FIGURE 1:**
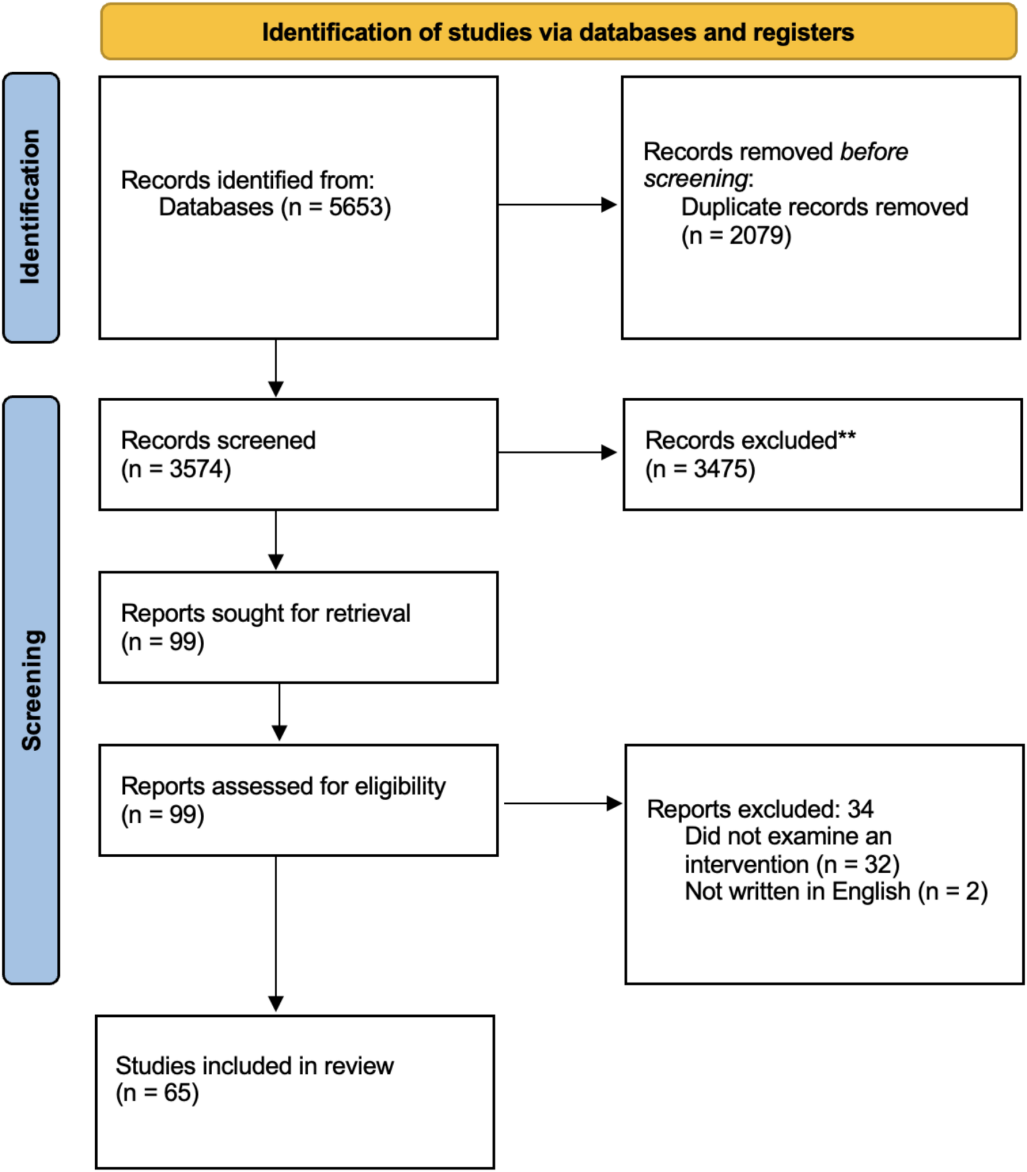
PRISMA 2020 flow diagram.

Studies, regardless of their design, publication date or length of follow‐up, were considered. Eligible studies needed to examine an intervention that targeted social functioning in psychosis. Studies were eligible for inclusion if they were published in peer‐reviewed journals, written in English and included participants with a psychotic disorder (SZ or FEP) diagnosed using a reliable psychometric tool (e.g., *Diagnostic and Statistical Manual of Mental Disorders*, fifth edition, or DSM‐5; American Psychiatric Association 2013; *International Classification of Diseases*, eleventh edition, or ICD‐11; World Health Organisation 2019). The study selection process is summarised in the PRISMA flow diagram (see Figure 1).

### 2.3 Exclusion Criteria

Studies written in languages other than English, unpublished or grey literature, conference abstracts, book chapters, meta‐analyses, systematic reviews, and case studies were excluded from this review. Studies were also excluded if patients had drug‐induced psychosis or psychosis due to organic causes. Lastly, studies were excluded if they did not investigate a social functioning intervention for psychosis.

### 2.4 Data Extraction Process

Relevant literature was de‐duplicated and imported into Microsoft Excel. An Excel spreadsheet was created to record the characteristics of studies. The extracted data included the following variables: author (name/year), country and type of study, sample size and setting, mean age (SD), questionnaires/diagnostic tools, main findings and clinical implications. The screening process was conducted independently by one reviewer (M.V.) and was subsequently cross‐checked by a second reviewer (A.G.).

### 2.5 Quality Appraisal

The methodological quality of the included studies was assessed using the Effective Public Health Practice Project (EPHPP) (Thomas et al. 2004) for quantitative studies (see Tables 1 and 4) and the Joanna Briggs Institute Critical Appraisal Checklist for Qualitative Research (Joanna Briggs Institute; Lockwood et al. 2015) for qualitative studies (see Table 2).

**TABLE 1:** Quality assessment for quantitative studies—EPHPP tool—for social functioning intervention studies.

Author (year)	Selection bias	Study design	Confounders	Data collection methods	Withdrawals and dropouts	Analyses (appropriateness)	Global rating
Abaoğlu et al. (2020)	Moderate	Strong	Moderate	Strong	Moderate	Yes	Moderate
Addington et al. (2023)	Strong	Strong	Weak	Strong	Moderate	Yes	Moderate
Adery et al. 2018)	Strong	Moderate	Moderate	Strong	Moderate	Yes	Moderate
Alvarez‐Jimenez et al. (2018)	Strong	Strong	Weak	Strong	Strong	Yes	Moderate
Alvarez‐Jimenez et al. (2021)	Strong	Strong	Strong	Strong	Strong	Yes	Strong
Atkinson et al. (1996)	Moderate	Strong	Weak	Moderate	Moderate	Yes	Moderate
Baskaran et al. (2023)	Strong	Strong	Weak	Strong	Strong	Yes	Moderate
Byrne et al. (2015)	Moderate	Moderate	Moderate	Strong	Strong	Yes	Moderate
Choi et al. (2017)	Moderate	Moderate	Weak	Strong	Strong	Yes	Moderate
Dabit et al. (2021)	Moderate	Moderate	Moderate	Strong	Strong	Yes	Moderate
Donohoe et al. (2018)	Moderate	Strong	Moderate	Strong	Moderate	Yes	Moderate
Dubreucq et al. (2020)	Moderate	Moderate	Strong	Strong	Weak	Yes	Moderate
Firth et al. (2018)	Moderate	Strong	Moderate	Weak	Strong	Yes	Moderate
Friedman‐Yakoobian et al. (2022)	Moderate	Strong	Weak	Strong	Moderate	Yes	Moderate
Fowler et al. (2009)	Strong	Moderate	Moderate	Strong	Moderate	Yes	Moderate
Fowler et al. (2018)	Strong	Strong	Strong	Strong	Strong	Yes	Strong
Fowler et al. (2019)	Moderate	Strong	Moderate	Strong	Strong	Yes	Moderate
Fulford et al. (2021)	Moderate	Moderate	Moderate	Strong	Strong	Yes	Moderate
Garety et al. (2006)	Moderate	Strong	Moderate	Strong	Strong	Yes	Moderate
Girón et al. (2010)	Moderate	Strong	Moderate	Strong	Strong	Yes	Moderate
Glenthøj et al. (2020)	Moderate	Strong	Moderate	Strong	Moderate	Yes	Moderate
Granholm et al. (2005)	Strong	Strong	Strong	Strong	Strong	Yes	Strong
Han and Lee (2022)	Moderate	Strong	Moderate	Weak	Strong	Yes	Moderate
Harder et al. (2014)	Strong	Moderate	Strong	Strong	Moderate	Yes	Moderate
Herman et al. (2018)	Moderate	Moderate	Moderate	Moderate	Moderate	Yes	Moderate
Hoşgelen et al. (2023)	Strong	Weak	Weak	Strong	Strong	Yes	Weak
Inchausti et al. (2018)	Strong	Strong	Strong	Strong	Strong	Yes	Strong
Karaman et al. (2020)	Moderate	Weak	Weak	Strong	Weak	Yes	Weak
Katsumi et al. (2019)	Moderate	Strong	Moderate	Strong	Weak	Yes	Moderate
Kern et al. (2020)	Moderate	Moderate	Moderate	Strong	Strong	Yes	Moderate
Kimhy et al. (2021)	Moderate	Moderate	Moderate	Strong	Moderate	Yes	Moderate
Lal et al. (2023)	Moderate	Moderate	Weak	Moderate	Strong	Yes	Weak
Lee et al. (2013)	Strong	Strong	Strong	Strong	Moderate	Yes	Moderate
Li et al. (2018)	Strong	Strong	Moderate	Strong	Moderate	Yes	Moderate
Mazzi et al. (2018)	Strong	Strong	Moderate	Weak	Strong	Yes	Moderate
Minor et al. (2022)	Moderate	Moderate	Moderate	Strong	Strong	Yes	Moderate
Nahum et al. (2014)	Moderate	Moderate	Moderate	Moderate	Moderate	Yes	Moderate
Nahum et al. (2021)	Strong	Strong	Strong	Moderate	Weak	Yes	Moderate
Nijman et al. (2023)	Strong	Strong	Strong	Strong	Weak	Yes	Moderate
Özer and Dişsiz (2024)	Strong	Strong	Moderate	Strong	Strong	Yes	Moderate
Pérez‐Aguado et al. (2024)	Strong	Moderate	Moderate	Strong	Strong	Yes	Moderate
Priebe et al. (2020)	Strong	Strong	Strong	Strong	Moderate	Yes	Moderate
Restek‐Petrović et al. (2014)	Weak	Moderate	Weak	Weak	Weak	Yes	Weak
Ruiz‐Iriondo et al. (2020)	Moderate	Strong	Strong	Strong	Strong	Yes	Moderate
Rus‐Calafell et al. (2013)	Strong	Strong	Moderate	Strong	Weak	Yes	Moderate
Rus‐Calafell et al. (2014)	Moderate	Moderate	Weak	Strong	Moderate	Yes	Moderate
Sarandöl et al. (2024)	Moderate	Strong	Strong	Strong	Strong	Yes	Moderate
Şenormancı et al. (2021)	Strong	Strong	Strong	Strong	Strong	Yes	Strong
Shen et al. (2022)	Strong	Strong	Weak	Strong	Moderate	Yes	Moderate
Shih and Yang (2023)	Strong	Strong	Moderate	Strong	Strong	Yes	Moderate
Shimada et al. (2016)	Moderate	Moderate	Strong	Strong	Strong	Yes	Moderate
Shimada et al. (2018)	Strong	Strong	Moderate	Weak	Strong	Yes	Moderate
Shimada et al. (2022)	Strong	Strong	Moderate	Strong	Weak	Yes	Moderate
Štrkalj‐Ivezić et al. (2013)	Weak	Strong	Moderate	Strong	Weak	Yes	Moderate
Tong et al. (2021)	Strong	Strong	Moderate	Strong	Strong	Yes	Moderate
Torres et al. (2002)	Strong	Strong	Moderate	Strong	Strong	Yes	Moderate
Varga et al. (2018)	Strong	Strong	Strong	Strong	Strong	Yes	Strong
Veltro et al. (2011)	Moderate	Strong	Strong	Strong	Weak	Yes	Moderate
Ventura et al. (2019)	Strong	Strong	Moderate	Strong	Moderate	Yes	Moderate
Wong et al. (2024)	Strong	Strong	Strong	Strong	Strong	Yes	Strong
Yildiz et al. (2004)	Moderate	Moderate	Weak	Moderate	Weak	Yes	Moderate
Yildiz et al. (2018)	Strong	Strong	Strong	Strong	Strong	Yes	Strong
Zhu et al. (2020)	Strong	Strong	Strong	Strong	Weak	Yes	Moderate
Zimmer et al. (2007)	Strong	Strong	Moderate	Strong	Strong	Yes	Moderate

Abbreviation: EPHPP, Effective Public Health Practice Project (Thomas et al. 2004).

**TABLE 2:** Quality Assessment for Qualitative studies—JBI tool—For Social Functioning Intervention Study.

Author (year)	1. Is there congruity between the stated philosophical perspective and the research methodology?	2. Is there congruity between the research methodology and the research question or objectives?	3. Is there congruity between the research methodology and the methods used to collect data?	4. Is there congruity between the research methodology and the representation and analysis of data?	5. Is there congruity between the research methodology and the interpretation of results?	8. Are participants, and their voices, adequately represented?	9. Is the research ethical according to current criteria or, for recent studies, and is there evidence of ethical approval by an appropriate body?	10. Do the conclusions drawn in the research report flow from the analysis, or interpretation, of the data?	Include if yes to 2–5, 8–10
Gee et al. (2023)	Yes	Yes	Yes	Yes	Yes	Yes	Yes	Yes	✓✓

Abbreviation: JBI, Joanna Briggs Institute Quality Assessment Tool (Lockwood et al. 2015).

## 3 Results

Of the 5653 papers initially identified through database screening, a total of 65 studies were included in this review. Table 3 summarises the characteristics and main findings of all included studies. Table 5 summarises the main findings from studies examining the factors associated with high or low social functioning in psychosis.

**TABLE 3:** Social Functioning Interventions in Psychosis (*n* = 65).

Author (year)	Country and type of study	Intervention, sample size, setting and follow‐ups	Mean age (SD)	Questionnaire and diagnostic tools	Main findings and clinical implications
Abaoğlu et al. (2020)	Turkey RCT	Individualised life skills training (*n* = 15) vs. control group (*n* = 17) 2 sessions per week for 8 weeks 32 SZ (19M/13F) Outpatients Pre‐ and post‐intervention	Individualised life skills training = 40.33 (11.90) Control group = 38.89 (9.38)	DSM‐5 Positive Negative Syndrome Scale (PANSS) Social Functioning Scale (SFS) Clinical Global Impression‐Severity (CGI) Functional Assessment Short Test (FAST) Activities of Daily Living (ADL) Instrumental Activities of Daily Living (IADL)	Statistically significant pre and post‐test mean scores were found in the intervention group compared to the control group for the following variables: Negative symptoms (*p* ≤ 0.01) General psychopathology (*p* ≤ 0.01) PANSS total (*p* < 0.001) Independence in activities of daily living (*p* = 0.02) Instrumental activities of daily living (*p* < 0.001) Individualised life skills training may be an effective therapeutic method for the rehabilitation of individuals diagnosed with schizophrenia and should be supported by long‐term follow‐up studies.
Addington et al. (2023)	Canada RCT	Cognitive behavioural social skills training (*n* = 70) vs. supportive therapy (*n* = 82) 18 weeks 152 CHR (69M/83F) Outpatients Pre‐intervention Post‐intervention 12‐month follow‐up	Cognitive behavioural social skills training = 17.36 (4.01) Supportive therapy = 17.49 (4.12)	Criteria of Psychosis Risk Syndromes (COPS) Structured Interview for Psychosis Risk Syndromes (SIPS) SCID‐5 Scale of Psychosis‐Risk Symptoms Negative symptoms (SOPS) Global Functioning: Social (GF:S) Global Functioning: Role (GF:R) Defeatist Performance Beliefs Scale (DPAS) Scale of Psychosis‐Risk Symptoms Positive Symptoms (SOPS +) Scale of Psychosis‐Risk Symptoms Negative symptoms (SOPS −) Calgary Depression Scale for Schizophrenia (CDSS) Social Anxiety Scale (SAS) Social Interaction Anxiety Scale (SIAS) Social Self‐Efficacy Scale (SSES) Asocial Beliefs Scale (ABS) Brief Core Schema Scale (BCSS other) Brief Core Schema Scale (BCSS self)	There were no significant between‐group differences in any outcome measure. For the key outcomes, there were statistically significant within‐group improvements for cognitive behavioural social skills training in social functioning (end of treatment, *p* ≤ 0.01; 12‐month follow‐up *p* ≤ 0.01) and defeatist beliefs (end of treatment, *p* ≤ 0.05) and for both groups in role functioning (12‐month follow‐up *p* ≤ 0.01). However, the within‐group changes in social and role functioning have to be viewed with caution as on average the changes were less than one point which suggests no clinical significance. Both groups showed significant improvement in positive and negative symptoms, depression and anxiety. There was minimal if any change in the self‐belief measures. A decline in social functioning is a significant predictor of later transition to psychosis, future work is needed to find effective treatments for this decline in functioning for CHR youth.
Adery et al. (2018)	America Preliminary study	Multimodal adaptive social intervention in virtual reality (*n* = 18) 10 sessions 18 SZ (10M/8F) Outpatients Pre‐ and post‐intervention	48.78 (7.42)	SCID‐5 National Adult Reading Test, Revised (NART‐R) Wechsler Abbreviated Scale of Intelligence (WASI) Brief Psychiatric Rating Scale (BPRS) Scale for the Assessment of Positive Symptoms (SAPS) Scale for the Assessment of Negative Symptoms (SANS) Social Functioning Scale (SFS)	Statistically significant pre and post‐test mean scores were found in the intervention group for the following variables: BPRS (*p* ≤ 0.01) SANS (*p* ≤ 0.01) The preliminary finding of reduced negative symptoms following 10‐session‐training highlights multimodal adaptive social intervention in virtual reality as an effective and accessible intervention for social impairments in schizophrenia. A randomised clinical trial in the future is needed to determine whether the multimodal adaptive social intervention in virtual reality training successfully targets social impairments.
Alvarez‐Jimenez et al. (2018)	Australia Uncontrolled single group	MOMENTUM (combines two novel approaches to social recovery: A strengths‐ and mindfulness‐based intervention within a social media environment and the application of self‐determination theory of motivation, *n* = 14) 2 months 14 UHR (3M/11F) Outpatients Pre‐intervention 2‐month follow‐up	20.30 (2.40)	Comprehensive Assessment of At Risk Mental States (CAARMS) Montgomery‐Asberg Depression Rating Scale (MADRS) Perceived Stress Scale (PSS) Global Functioning: Social (GF:S) Freiburg Mindfulness Inventory (FMI) Strengths Use Efficacy Scale (SUS) Social Provisions Scale (SPS) Attachment Social integration Worth reassurance Reliable alliance Guidance Nurturance Satisfaction With Life Scale (SWLS) Self‐Efficacy Scale (SES) Self‐Esteem Rating Scale‐Short Form (SERS‐SF) UCLA Loneliness Scale (UCLA)	Statistically significant pre‐intervention and 2‐month follow‐up mean scores were found in the intervention group: GF:S (*p* < 0.01) SUS (*p* = 0.03) FMI (*p* = 0.04) Attachment (*p* ≤ 0.05) SWLS (*p* = 0.03) Nurturance (*p* ≤ 0.01) Guidance (*p* = 0.03) The intervention appeared to engage the theoretical target mechanisms and the large and reliable improvement in social functioning observed during the study was correlated with indicators of system usage. A large randomised controlled trial is needed to strengthen these current findings.
Alvarez‐Jimenez et al. (2021)	Australia Parallel‐group, single‐blind RCT	Horyzons (moderated online social therapy model, which combines interactive online therapy, peer‐to‐peer social networking, peer moderation and expert support) plus TAU (*n* = 86) vs. TAU (*n* = 84) 18 months 170 FEP (90M/80F) Outpatients Pre‐intervention 6‐, 12‐ and 18‐month follow‐ups	Horyzons plus TAU = 21.01 (2.93) TAU group = 20.81 (2.83)	Positive Negative Syndrome Scale (PANSS) SCID‐2 Calgary Depression Scale for Schizophrenia (CDSS) Personal and Social Performance Scale (PSP) UCLA Loneliness Scale (UCLA) Medical Outcomes Study Social Support Survey (MOS‐SSS) Self‐Esteem Rating Scale‐Short Form (SERS‐SF) Mental Health Confidence Scale (MHCS) Satisfaction With Life Scale (SWLS) Assessment of Quality of Life (AQoL)	There were no significant between‐group differences in any outcome measure. Additionally, there were no significant improvements in outcome measure within‐groups. However, a secondary analysis revealed statistically significant pre‐intervention and 2‐month follow‐up test mean scores were found in the intervention group compared to the TAU group for the following variables: Vocational or educational recovery (*p* = 0.04) Visits to emergency services (*p* = 0.03) Horyzons holds significant promise as a novel, engaging and sustainable intervention to improve vocational recovery, reduce utilisation of emergency services and provide continuous support for young people with FEP beyond specialised care. Further research is required to determine the optimal features and operations of online social media‐based interventions so that they support connectedness and meaningful engagement.
Atkinson et al. (1996)	England Preliminary study	Education group (*n* = 57) vs. waiting list (*n* = 73) 20 weeks 130 SZ (82M/48F) Outpatients Pre‐ and post‐intervention 3‐month follow‐up	Age breakdown not reported	Social Avoidance and Distress Scale (SADS) Global Assessment Scale (GAS) Quality Of Life according to Heinrichs et al.'s scale 1984 (QOL) Social Functioning Scale (SFS) Social Network Schedule (SNS) DSM‐3	Statistically significant pre and post mean scores were found in the intervention group compared to the waiting list group for the following variables: QOL (*p* ≤ 0.05) SNS (*p* ≤ 0.05) Statistically significant post‐group and follow‐up mean scores were found in the intervention group compared to the waiting list group for the following variables: QOL (*p* ≤ 0.01) SFS (*p* ≤ 0.05) Education groups for people with schizophrenia have a positive impact on social functioning, social networks and quality of life. It must be noted that the groups were most acceptable to patients with high compliance with medication. Therefore, future research is needed to further strengthen these findings.
Baskaran et al. (2023)	India Experimental study	Social skills programme (*n* = 20) vs. TAU (*n* = 20) 1 week 40 SZ (24M/16F) Inpatients Pre‐ and post‐intervention	Age breakdown not reported	DSM‐5 ICD‐10 Vellore Assessment of Social Performance scale (VASP)	Statistically significant pre and post‐test mean scores were found in the intervention group compared to the TAU group for the following variables: Nonverbal social skills (*p* < 0.001) Verbal social skills (*p* < 0.001) Receptive social competence skills (*p* < 0.001) Processing social competence skills (*p* < 0.001) Expressive social competence skills (*p* < 0.001) Total social performance skills (*p* < 0.001) Social skills programme effectively improved social performance among schizophrenia in‐patients and can be implemented as part of routine care therapy for the holistic treatment of these patients.
Byrne et al. (2015)	China RCT	Cognitive remediation computerised drill training programme plus TAU (*n* = 20) vs. TAU (*n* = 20) 6 weeks 40 SZ (All M) Outpatients Pre‐ and post‐intervention	Cognitive remediation computerised drill training programme plus TAU = 38.3 (12.5) TAU group = 37.0 (8.1)	DSM‐4 Positive Negative Syndrome Scale (PANSS) Clinical Global Impression Scale (CGI) Personal and Social Performance Scale (PSP) Letter‐Number Sequencing Task (LNST) Hong Kong List Learning Test (HKLLT) FAR (Facial Affect Recognition)	Statistically significant pre and post‐test mean scores were found in the intervention group compared to the TAU group for the following variables: PSP variable (*p* = 0.02) Fearful faces: number correct (*p* = 0.03) Computer administrated cognitive remediation programme improved recognition of facial emotions by individuals with schizophrenia and, thus, improve their social functioning. But more work on developing the training modules and on testing in larger, more diverse samples will be needed before this can be recommended as a standard part of cognitive remediation programmes.
Choi et al. (2017)	America Double‐blind RCT	Processing speed training (is a tablet‐based programme that uses pupillometry‐based neurofeedback to improve visual scanning efficiency, *n* = 32) vs. active control (exposure to computers and clinician contact, *n* = 30) 30 h over the course of 2 months 62 CHR (All F) Outpatients Pre‐ and post‐intervention 4‐month follow‐up	Processing speed training = 18.17 (3.81) Active control group = 18.53 (3.72)	Structured Interview for Prodromal, Syndromes/Scale of Prodromal Symptom (SIPS/SOPS) Wechsler Adult Intelligence Scale‐Third Edition (WAIS‐III) Minnesota Clerical Test (MCT) Continuous Performance Test‐Identical Pairs (CPT‐IP) Social Adjustment Scale‐Self Report (SAS‐SR) Social Anxiety Scale (SAS) Beck Depression Inventory‐II (BDI‐II)	Statistically significant post‐intervention and 2‐month follow‐up mean scores were found in the intervention group compared to the active control group for the following variable: SAS‐SR (*p* ≤ 0.01) WAIS‐III (*p* ≤ 0.01) The study was the first to test focal neurofeedback‐based cognitive training for processing speed deficits in the putatively prodromal phase of schizophrenia to address associated social morbidity. Targeting processing speed appears to be a promising pathway to decreasing co‐morbidity and mitigating a risk factor for psychosis.
Dabit et al. (2021)	Online Parallel arm, double‐blind RCT	CLIMB (a clinician‐assisted, computerised social cognition training, ecological momentary assessments, group teletherapy and moderated messaging, *n* = 12) vs. active control (computerised general cognitive training, unstructured support groups and unmoderated messaging, *n* = 12) 9 weeks 31 SSD (11M/16F/4B) Outpatients Pre‐ and post‐intervention	CLIMB = 39.47 (12.06) Control group = 35.13 (9.13)	DSM‐5 Positive Negative Syndrome Scale (PANSS) Abbreviated Quality of Life Scale (aQLS) Social Functioning Scale (SFS) Columbia‐Suicide Severity Rating Scale (C‐SSRS)	As a group, participants showed significant improvements in SFS (*p* ≤ 0.05), with no between‐group differences. Intent‐to‐treat analyses indicated greater improvements in aQLS (*p* = 0.03) for the active control compared to the intervention group. Further research is required with larger sample sizes and more balanced pre‐intervention characteristics to evaluate the potential of mobile interventions for social functioning and better understand the implications for improving quality of life in SSD.
Donohoe et al. (2018)	England RCT	Cognitive remediation (*n* = 48) vs. control group (*n* = 42) 8 weeks 90 SSD (36M/54F) Outpatients Pre‐intervention 2‐week follow‐up 3–6‐month follow‐up	Cognitive remediation = 43.5 (11.5) Control group = 43.1 (11.0)	SCID‐4 Letter Number Sequencing (LNS) Wechsler Abbreviated Scale of Intelligence (WASI) Stockings Of Cambridge task (SOC) Social and Occupational Functional Assessment Scale (SOFAS) Independent Living Scale, Problem‐Solving scale (ILS‐PS) UCSD Performance Based Skills Assessment Brief (UPSA‐B)	Statistically significant mean scores were found in the intervention group compared to the control group for the following variables: SOFAS (3–6‐month follow‐up, *p* < 0.001) ILS‐PS (2‐week post‐treatment, *p* = 0.03, 3–6‐month post‐treatment, *p* ≤ 0.05) UPSA‐B (2‐week post‐treatment, *p* ≤ 0.01) Cognitive remediation training was reliably associated with improved cognitive performance on non‐trained tasks and with changes in cortical networks, particularly in terms of enhanced inter‐hemispheric connectivity within prefrontal and parietal cortex. These benefits are, furthermore, associated with benefits to social and occupational functioning, aspects of psychosis related disability not addressed by pharmacological treatments. Given the limited resources available within many health services for delivering cognitive remediation training, the evidence suggests that even limited amounts of training, delivered remotely and with low support, can lead to benefits. A follow‐up study is needed to determine the duration of the effectiveness of the intervention.
Dubreucq et al. (2020)	France Controlled quasi experimental	Exercise‐enriched integrated social cognitive remediation (RemedRugby, *n* = 57) vs. active control (practising Touch Rugby, *n* = 30) 12 weekly 2 h sessions 87 SZ (70M/17F) Outpatients Pre‐ and post‐intervention	RemedRugby group = 33.3 (8.8) Active control group = 33.3 (7.9)	DSM‐5 Personal and Social Performance Scale (PSP) Movie for the Assessment of Social Cognition (MASC) Social Perception test (PerSo) The Ambiguous Intentions Hostility Questionnaire (AIHQ) Questionnaire of Cognitive and Affective Empathy (QCAE) Self‐Assessment of Social cognition (ACSo) Social Cognition Functional Outcomes Scale (ERF‐CS) Positive Negative Syndrome Scale (PANSS) Internalised Stigma of Mental Illness scale (ISMI) Self‐Esteem Rating Scale‐Short Form (SERS‐SF) Boston University Empowerment Scale (BUES) Stage of recovery instrument (STORI) Working memory, AXE‐CPT, Executive control (WAIS‐IV) Trail Making Test (TMT‐A and B)	Statistically significant pre and post‐test mean scores were found in the intervention group compared to the active control group for the following variables: MASC (*p* = 0.02) PANSS (*p* = 0.02) Negative symptoms (*p* < 0.001) PSP (*p* < 0.001) AIHQ (*p* = 0.01) An integrated exercise‐enriched social cognitive remediation programme was effect in improving social functioning. Future research should investigate the potential effectiveness of the intervention in the long‐term.
Firth et al. (2018)	England Feasibility study	Exercise intervention (*n* = 31) vs. TAU (*n* = 7) 2 days/week, 90 min for 10 weeks 38 FEP (30M/8F) Outpatients Pre‐ and post‐intervention	Exercise group = 25.8 (4.6) Control group = 25.9 (5.9)	Beck Depression Inventory‐II (BDI‐II) Social Interaction Anxiety Scale (SIAS) WHO Disability Assessment Scale 2.0 (WHODAS) WHO Quality of Life Brief Assessment (WHOQOL‐BREF) Social and Occupational Functional Assessment Scale (SOFAS) Positive Negative Syndrome Scale (PANSS) Short‐term memory (STM)	Statistically significant pre and post‐test mean scores were found in the intervention group compared to TAU group for the following variables: PANSS (*p* = 0.01) SOFAS (*p* < 0.001) The findings indicate the intervention could improve physical, psychological and social outcomes for young adults diagnosed with FEP. Future work should investigate novel methods for connecting EI services with exercise resources and for embedding exercise specialists within EI services, in order to maximise accessibility and broaden possibilities for delivering such interventions in clinical practice.
Friedman‐Yakoobian et al. (2022)	America RCT	CLUES (Cognition for Learning and for Understanding Everyday Social Situations, *n* = 20) vs. EnACT (Enriched Acceptance and Commitment Therapy, *n* = 18) 6 months 38 CHR (23M/10F/5B) Outpatients Pre‐ and post‐intervention 3‐month follow‐up	CLUES group = 19.2 (2.92) EnACT group = 19.1 (3.02)	Criteria of Psychosis Risk Syndromes (COPS) Structured Interview for Psychosis Risk Syndromes (SIPS) Wide Range Achievement Test 4th Edition Word Reading Subtest (WRAT) Global Functioning Scale: Social and Role (GFS) Brief Assessment of Cognition in Schizophrenia (BACS) Letter Number Sequencing (LNS) Hopkins Verbal Learning Test (HVLT) Brief Visuospatial Memory Test (BVMT) Mayer‐Salovey‐Caruso Emotional Intelligence Test (MSCEIT) Orientation Remedial Module reaction time test (ORM) The Awareness of Social Inference Test (TASIT)	There was a significant treatment group by time interaction for the GFS at post‐intervention (*p* = 0.02) and at 3‐month follow‐up (*p* = 0.02) both favouring CLUES. Analysis of the linear trend across all time points (including 3‐month follow‐up) revealed a significant overall time by treatment group interaction for GFS (*p* = 0.02) favouring CLUES. For TASIT variable, there was a significant interaction at post‐intervention (*p* = 0.02) also favouring CLUES. For the MSCEIT managing emotions subtest, there was a significant treatment by time interaction at 3‐month follow‐up (*p* = 0.03) favouring CLUES. In regard SIPS both groups at post‐intervention (*p* < 0.001) and follow‐up (*p* < 0.001). Future studies of CLUES investigating larger samples of participants will be important for determining the potential impact of this treatment on outcomes and the utility of CLUES as a treatment for youth at clinical high risk for psychosis.
Fowler et al. (2009)	England Single‐blind RCT	Cognitive behaviour therapy plus TAU (*n* = 35) vs. TAU alone (*n* = 42) 12 sessions over 9 months 77 FEP (55M/22F) Outpatients Pre‐ and post‐intervention	Cognitive behaviour therapy plus TAU group = 27.8 (6.1) TAU group = 30.0 (7.2)	Positive Negative Syndrome Scale (PANSS) Structured Activity Constructive Economic Activity Beck Hopelessness Scale (BHS) Quality of Life Scale (QoLS) Role Functioning Camberwell Assessment of Needs (CAN Number of Needs) Beck Depression Inventory (BDI) Beck Anxiety Inventory (BAI)	Statistically significant pre and post‐test mean scores were found in the intervention group compared to TAU group for the following variables: Structured Activity (*p* < 0.001) Constructive Economic Activity (*p* < 0.05) PANSS (*p* < 0.05) CAN Number of Needs (*p* < 0.05) The results indicate the possible promise of undertaking further research as CBT plus TAU seems to be a feasible intervention to improve activity in non‐affective psychosis. A further large‐scale trial of this intervention is needed.
Fowler et al. (2018)	England Single‐blind RCT	Social recovery therapy plus early intervention services (*n* = 79) vs. early intervention services alone (*n* = 75) 16 sessions over 9 months 154 FEP (116M/38F) Outpatients Pre‐intervention Post‐intervention 15‐month follow‐up (6‐month follow‐up)	EIS plus social therapy condition group = 24.84 (SD not reported) EIS alone condition group = 24.15 (SD not reported)	Time Use Survey (TUS) Structured Activity Constructive Economic Activity Positive Negative Syndrome Scale (PANSS) Scale for the Assessment of Negative Symptoms (SANS) Beck Depression Inventory (BDI) Social Interaction Anxiety Scale (SIAS) Beck Hopelessness Scale (BHS) Adult Trait Hope Scale (ATHS) Meaning in Life Questionnaire (MLQ) The Role and Social Global Functioning Scale (GF:S/GF:R)	Statistically significant pre and post‐test mean scores were found in the intervention group compared to EIS alone group for the following variables: Structured Activity (*p* = 0.01) Constructive Economic Activity (*p* = 0.03) Negative PANSS (*p* = 0.03) General PANSS (*p* = 0.04) SANS total (0.04) SIAS (*p* = 0.02) BHS (*p* = 0.02) Statistically significant 15‐month follow‐up mean scores were found in the intervention group compared to EIS alone group for the following variables: BHS (*p* = 0.02) ATHS total score (*p* = 0.01) MLQ total score (*p* = 0.04) Structured activity (*p* = 0.04) Constructive economic activity (*p* = 0.05) The results suggest that social recovery therapy techniques could be a useful addition in this group. A larger more definitive trial is required to examine long‐term effects more clearly. The extent to which interventions of this type might be enhanced by top‐up therapy or a greater number of sessions over a longer duration could be worthy of further scrutiny in view of evidence from similar studies in adults with more established schizophrenia.
Fowler et al. (2019)	England Single‐blind RCT	Social recovery therapy plus early intervention services (SRT + TAU, *n* = 35) vs. early intervention services alone (TAU, *n* = 42) 16 sessions over 9 months 77 SSD (Gender distribution not reported) Outpatients Pre‐intervention Post‐intervention 15‐month follow‐up (6‐month follow‐up)	Age breakdown not reported	Time Use Survey (TUS) Positive Negative Syndrome Scale (PANSS) Beck Hopelessness Scale (BHS)	In the combined sample of individuals with affective and non‐affective psychosis, more individuals in the SRT + TAU group had engaged in paid work over the 15 months since the end of the intervention period compared to the TAU alone group (31.0% vs. 16%). However, there were no significant differences between the SRT + TAU and TAU alone groups in terms of engagement in work, education or voluntary work. There were no significant differences between the SRT + TAU and TAU alone groups for those with affective psychosis in terms of frequency of engagement in paid work (44.4% vs. 46.2%). At 2‐year follow‐up there was a strong trend suggesting an allocation by diagnosis interaction for hopelessness, with the non‐affective psychosis treatment group scoring lower on the BHS than individuals in the non‐affective psychosis control group (*p* = 0.08). Further research is also necessary to explore whether SRT could be effective for individuals at a later stage of illness, outside of Early Intervention Services.
Fulford et al. (2021)	USA Pilot intervention	Motivation and Skills Support smart app (*n* = 31) 8 weeks 31 SSD (16M/15F) Outpatients Pre‐ and post‐intervention 3‐month follow‐up	46 (11)	Social Functioning Scale (SFS) Quality of Life Scale Interpersonal Relations (QLS‐IR) Clinical Assessment Interview for Negative Symptoms‐Motivation and Pleasure (CAINS‐MAP) Brief Psychiatric Rating Scale (BPRS)	Statistically significant pre and post‐test mean scores were found in the intervention group for the following variables: SFS (*p* = 0.02) BPRS (*p* = 0.02) Statistically significant 3‐month follow‐up mean scores were found in the intervention group for the BPRS variable (*p* = 0.03). The motivation and Skills Support smart app demonstrated promise as a stand‐alone mobile intervention and ultimately used as a supplemental intervention designed to address social functioning needs in people with schizophrenia. Further research is needed to strengthen the findings.
Garety et al. (2006)	England RCT	Early onset team (*n* = 71) vs. standard care (*n* = 73) 18 months 144 SSD (45M/99F) Outpatients Pre‐intervention 18‐month follow‐up	26 (SD not reported)	ICD–10 Positive Negative Syndrome Scale (PANSS) Global Assessment of Functioning (GAF) Calgary Depression Rating Scale (CDRS)	Statistically significant pre‐test and follow‐up mean scores were found in the intervention group compared to the standard care group for the following variables: Negative symptoms (*p* ≤ 0.05) GAF (*p* ≤ 0.01) Social and clinical outcomes in early psychosis can be improved by the provision of an assertive outreach team offering a package of evidence‐based treatments and care. Service users report a better quality of life and greater satisfaction with an early intervention service than with generic sector teams. A systematic approach to monitoring and preventing the early development of persisting psychotic symptoms should be taken.
Gee et al. (2023)	England RCT Qualitative study	Social recovery therapy plus early intervention services (*n* = 14) vs. early intervention services alone (*n* = 5) (intervention duration not reported) 19 FEP (13M/6F) Outpatients Pre‐intervention 9‐month follow‐up 15‐month follow‐up	26 (SD not reported)	N/A	The six themes identified were used to generate an explanatory model of SRT's enhancement of social recovery. Participant experiences highlight the importance of the therapist cultivating increased self‐understanding and assertively encouraging clients to face feared situations in a way that is perceived as supportive, while managing ongoing symptoms. The sense of achievement generated by reaching targets linked to personally meaningful goals promotes increased self‐agency and generates hope and optimism. The findings suggest potentially important processes through which social recovery was enhanced in this trial. Further research is needed to ensure the benefits observed are replicated.
Girón et al. (2010)	Spain RCT	Psychosocial family intervention, individual counselling plus TAU (*n* = 50) vs. individual counselling plus TAU (*n* = 50) 2 years 50 SZ (37M/13F) Outpatients Pre‐intervention 9‐month follow‐up 24‐month follow‐up	Psychosocial family intervention, individual counselling plus TAU group = 30.92 (6.98) Individual counselling plus TAU group = 32.12 (9.05)	DSM‐4 Psychiatric Assessment Scale (PAS) WHO Disability Assessment Scale 2.0 (WHO‐DAS) Quality of Life Scale (QoLS) Global Assessment of Functioning (GAF) Quantity of Useful Work item of the Strauss and Carpenter Prognostic Scale Pre‐morbid Adjustment Scale Spanish version of the Social Behaviour Assessment Schedule (SBAS)	Family intervention significantly reduced the number of clinical relapses (*p* = 0.02), major incidents (*p* < 0.01), positive (*p* < 0.001) and negative symptoms (*p* < 0.01) and admissions to hospital (*p* = 0.03), improved GAF (*p* = 0.05) and relieved family burden (*p* < 0.01), compared to the Individual counselling group at the 24‐month follow‐up. The results of the study could be interpreted in the light of the stress‐vulnerability model of schizophrenia (Zubin and Spring 1977; Nuechterlein et al. 1992). This model postulates that psychotic episodes result from the interaction between the individual vulnerability of the patient and the level of environmental stress the patient is exposed to. According to this model, the improvement in patients' clinical conditions and social functioning may be related to changes in relatives' attitudes, as a result of the psycho‐social intervention. This hypothetical relationship between family intervention and change in family behaviour and attitudes will be the subject of a future study.
Glenthøj et al. (2020)	Denmark RCT	Cognitive remediation plus TAU (*n* = 73) vs. TAU (*n* = 73) 20 weeks 146 UHR (64M/82F) Inpatients and outpatients Pre‐intervention 6‐month follow‐up 12‐month follow‐up	Cognitive remediation plus TAU group = 23.93 (4.67) TAU group = 23.90 (3.79)	Comprehensive Assessment of At Risk Mental State (CAARMS) Scale for the Assessment of Negative Symptoms (SANS) Montgomery‐Asberg Depression Rating Scale (MADRS) Young Mania Rating Scale (YMRS) Scale of Social Skills for Psychiatric Inpatients (SSPI) Social Responsiveness Scale (SRS) Assessment of Quality of Life (AQoL) Emotion Recognition Task (ERT) The Awareness of Social Inference Test (TASIT) Social Cognition Screening Questionnaire (SCSQ) High Risk Social Challenge (HiSoC) Personal and Social Performance Scale (PSP) social and Occupational Functional Assessment Scale (SOFAS) Global Functioning Scale (GFS) Brief Assessment of Cognition in Schizophrenia (BACS) Cambridge Neuropsychological Test Automated Battery (CANTAB) Paired Associates Learning (PAL) Emotion Recognition Test (ERT) Behaviour Rating Inventory of Executive Functions – Adult Version (BRIEF‐A) Client Satisfaction Questionnaire (CSQ)	Statistically significant pre‐intervention and 6‐month follow‐up mean scores were found in the intervention group compared to the TAU group for the following variables: ERT latency total (*p* = 0.02) ERT latency happiness (*p* < 0.001) ERT latency sadness (*p* = 0.01) ERT latency fear (*p* = 0.01) At 12 months, the intervention group exhibited significantly better performance on two measures of executive function (*p* = 0.03) and visual memory (*p* = 0.02). The findings point to the need for investigating the effect of more individualised and possibly more focused cognitive remediation along with the intensity and dosage of cognitive remediation needed to more broadly benefit cognition as well as functioning and symptoms.
Granholm et al. (2005)	America RCT	Cognitive Behavioural Social Skills Training plus TAU (*n* = 37) vs. TAU (*n* = 39) 24 weekly group sessions over 6 months 76 SSD (56M/20F) Outpatients Pre‐intervention 3‐month follow‐up during intervention Post‐intervention	Cognitive Behavioural Social Skills Training plus TAU group = 54.5 (7.0) TAU group = 53.1 (7.5)	DSM‐4 Hamilton Depression rating scale (HAM‐D) Independent Living Skills Survey (ILSS) Performance Based Skills Assessment Brief (UCSD) Positive Negative Syndrome Scale (PANSS) Beck Cognitive Insight (BCI)	Statistically significant pre and post‐test mean scores were found in the intervention group compared to the TAU group for the following variables: Independent Living Skills Survey (*p* = 0.02) BCI (*p* < 0.001) UCSD (*p* = 0.05) Researchers should continue to develop and test group and individual cognitive behaviour therapy interventions that are tailored to the unique needs of different subgroups of patients with schizophrenia and identify which treatments are most effective for which patients and in what circumstances.
Han and Lee (2022)	Korea Pretest‐post‐test quasi experimental study	Metacognitive Intervention for Schizophrenia plus TAU (*n* = 25) vs. TAU (*n* = 20) 10 sessions for 10 weeks 43 SZ (30M/13F) Outpatients Pre‐ and post‐intervention 4‐week follow‐up	Metacognitive Intervention for Schizophrenia plus TAU group = 47.42 (10.35) TAU group = 42.95 (10.26)	DSM‐5 Psychotic Symptom Rating Scale Personal and Social Performance Scale (PSP) Beck Cognitive Insight (BCI)	Statistically significant pre and post‐test mean scores were found in the intervention group compared to the TAU group for the following variables: Auditory hallucinations (*p* < 0.001) Delusions (*p* < 0.001) PSP (*p* < 0.001) Socially useful activities (*p* < 0.001) Personal and social relationships (*p* < 0.001) There is a need for further research on the metacognitive Intervention for Schizophrenia with a larger sample size. Follow‐up assessments should be conducted at 3, 6 and 12 months to determine the programme's lasting effects.
Harder et al. (2014)	Denmark Prospective, longitudinal, comparative, multicentre investigation	Supportive psychodynamic psychotherapy plus early intervention service (*n* = 119) vs. early intervention service alone (*n* = 150) 3 years 269 FEP (181M/88F) Outpatients Pre‐intervention 1‐year follow‐up 2‐year follow‐up 5‐year follow‐up	Supportive psychodynamic psychotherapy plus early intervention service group = 24.6 (SD not reported) Early intervention service alone group = 23.2 (SD not reported)	ICD‐10 Global Assessment of Functioning (GAF) Positive Negative Syndrome Scale (PANSS)	A significant treatment effect on GAF‐functioning was found at Year 1 (*p* = 0.01) and Year 2 (*p* = 0.01) in the intervention group compared to the early intervention group. Similarly, a significant treatment effect on GAF‐symptom was found at Year 1 (*p* = 0.05) and Year 2 (*p* < 0.001) in the intervention group compared to the early intervention group. Additionally, a significant treatment effect on PANSS was found at Year 2 (*p* = 0.04) in the intervention group compared to the early intervention only group. Future research should further seek to link the therapeutic interventions in supportive psychodynamic psychotherapy to specific developmental processes in the person suffering from psychosis and to explore which interventions are the most instrumental in furthering psychological and social recovery.
Herman et al. (2018)	Canada Feasibility study	Cognitive behavioural social skills training (*n* = 22) 18 sessions 22 FEP (13M/9F) Outpatients Pre‐ and post‐intervention 3‐month follow‐up	25.0 (3.8)	Multnomah Community Ability Scale (MCAS) Independent Living Skills Survey (ILSS) Recovery Assessment Scale (RAS) Satisfaction with Life Scale (SLS) Brief Psychiatric Rating Scale (BPRS) Global Assessment Scale (GAS) Global Assessment of Functioning (GAF) Beck Cognitive Insight (BCI)	Statistically significant pre and post mean scores were found in the intervention group for the following variables: ILSS (*p* < 0.001) MCAS (*p* < 0.001) GAF (*p* < 0.01) RAS (*p* = 0.02) SLS (*p* < 0.001) GAS (*p* < 0.001) Statistically significant 3‐month follow‐up mean scores were found in the intervention group for the following variables: ILSS (*p* < 0.01) MCAS (*p* < 0.001) GAF (*p* < 0.01) RAS (*p* = 0.01) SLS (*p* = 0.01) GAS (*p* < 0.001) The preliminary findings warrant further testing in a larger trial to determine efficacy.
Hoşgelen et al. (2023)	Turkey Preliminary study	VR Psychosocial treatment programme (*n* = 7) Semiweekly for 5 weeks 7 SZ (5M/2F) Outpatients Pre‐ and post‐intervention	43 (7)	DSM‐5 Personal and Social Performance Scale (PSP) Positive Negative Syndrome Scale (PANSS) Social Skills Checklist (SSC)	Statistically significant pre and post‐test mean scores were found in the intervention group for the PSP variable (*p* = 0.02). In the near future, cognitive improvement programmes and psychosocial functioning treatments are needed to be researched further as they may be provided through VR and could help patients cope with their symptoms and day‐to‐day challenges.
Inchausti et al. (2018)	Spain Single‐blind RCT	Metacognition‐oriented social skills training (MOSST, *n* = 36) vs. social skills training (*n* = 33) 16 group sessions 69 SSD (38M/31F) Outpatients Pre‐ and post‐intervention 6‐month follow‐up	MOSST group = 38.08 (12.09) Social skills training group = 37.30 (13.01)	ICD‐10 Social and Occupational Functional Assessment Scale (SOFAS) Personal and Social Performance Scale (PSP) Metacognitive Assessment Scale‐Abbreviated (MAS‐A) Positive Negative Syndrome Scale (PANSS) Beck Depression Inventory‐II (BDI‐II) Beck Anxiety Inventory (BAI)	Statistically significant pre and post mean scores were found in the intervention group compared to the social skills training group for the following variables: SOFAS (*p* < 0.01) PSP (*p* < 0.01) MAS‐A (*p* < 0.01) BDI‐II (*p* < 0.01) BAI (*p* < 0.01) Statistically significant 6‐month follow‐up mean scores were found in the intervention group for the following variables: SOFAS (*p* < 0.01) PSP (*p* < 0.01) MAS‐A (*p* < 0.01) BDI‐II (*p* < 0.01) BAI (*p* < 0.01) MOSST appears to have short‐ and long‐term beneficial effects on social functioning and symptoms. Further studies are required to replicate the current results in other samples.
Karaman et al. (2020)	Turkey Comparative study	Psychosocial skills training plus routine case management and occupational therapy (*n* = 21) vs. routine case management and occupational therapy (*n* = 22) vs. control group (*n* = 21) 18 sessions were completed between 10 and 18 weeks 100 SZ (42M/22F) Outpatients Pre‐ and post‐intervention	Psychosocial skills training plus routine case management and occupational therapy and routine case management and occupational therapy group = 35.62 (12.81) Routine case management and occupational therapy group = 43.23 (9.35) Control condition group = 38.95 (8.23)	DSM‐5 Personal and Social Performance Scale (PSP) Social Functioning Scale (SFS) Positive Negative Syndrome Scale (PANSS) Patient perspective ‐Social Functioning Scale (P‐SFS) Relative Perspective—Social Functioning Scale (R‐SFS)	There was no statistically significant difference between pretest and post‐test measurements of the control group for all measures. In analyses of intervention groups, a statistically significant decrease was determined in PANSS scores (*p* < 0.05) at the end of follow‐up. Additionally, there was a statistically significant increase in PSP (*p* < 0.05), P‐SFS (*p* < 0.05) and R‐SFS (*p* < 0.05) scores of intervention groups. Larger samples and randomised controlled trials are needed to extend the study's findings.
Katsumi et al. (2019)	Japan RCT	Neuropsychological educational approach to cognitive remediation (*n* = 22) vs. control group (provided with conventional drug therapy and either day care or occupational therapy, *n* = 22) 19 sessions 44 SSD (26M/18F) Outpatients Pre‐ and post‐intervention 6‐month follow‐up 9‐month follow‐up 15‐month follow‐up	Neuropsychological educational approach to cognitive remediation group = 37.5 (9.1) Control group = 38.0 (9.1)	ICD‐10 Japanese Adult Reading Test (JART) Positive Negative Syndrome Scale (PANSS) Brief Assessment of Cognition in Schizophrenia (BACS) Global Assessment of Functioning (GAF)	Statistically significant pre and post‐test mean scores were found in the intervention group compared to the control group for the BACS variable (*p* < 0.001). Statistically significant 15‐month follow‐up mean scores were found in the intervention group compared to the control group for the GAF variable (*p* < 0.001). Future research is necessary to assess the effectiveness of concurrent implementation of cognitive remediation and psychiatric rehabilitation on social functions.
Kern et al. (2020)	America RCT	Aerobic exercise programme (*n* = 35) vs. non‐aerobic stretching exercise (*n* = 18) 36 sessions 40 min conducted 3 times per week over 12 weeks 53 SSD (51M/2F) Outpatients Pre‐ and post‐intervention	Aerobic exercise programme group = 56.3 (6.2) Non‐aerobic stretching exercise group = 55.7 (7.1)	SCID‐5 Maximal Oxygen consumption (VO_2_ max) Continuous Performance Test‐Identical Pairs (CPT‐IP) Speed of processing (BACS) Working memory (WAIS‐IV) Executive control (AXE‐CPT) Brief Psychiatric Rating Scale (BPRS) Birchwood Social Functioning Scale (BSFS) Neurotrophic factor (BDNF) Body Mass Index (BMI) Theory Of Mind (TOM) The Awareness of Social Inference Test (TASIT)	Statistically significant pre and post‐test mean scores were found in the intervention group compared to the non‐aerobic exercise group for the following variables: VO_2_ max (*p* = 0.02) BMI (*p* = 0.03) CPT‐IP (*p* = 0.02) Future studies are needed to explore the mechanism(s) by which AE leads to improvements in social functioning by exploring the role of candidate mediators. Additionally, the findings from the study need to be replicated in larger randomised controlled trials, to support implementation on a broader scale.
Kimhy et al. (2021)	America Single‐blind RCT	Aerobic exercise plus TAU (*n* = 13) vs. TAU (*n* = 13) 12 weeks, 3 days a week for 1 h 33 SSD (21M/12F) Outpatients Pre‐intervention Post‐intervention, after 12 weeks	Aerobic exercise plus TAU group = 36.56 (10.37) TAU group = 37.24 (9.85)	DSM‐4 Scale for the Assessment of Positive Symptoms (SAPS) Scale for the Assessment of Negative Symptoms (SANS) Beck Depression Inventory (BDI) Beck Anxiety Inventory (BAI) Provision of Social Relations Scale (PSRS) Specific Levels of Functioning Scale (SLOF) Heinrichs‐Carpenter Quality of Life Scale (QLS)	Statistically significant pre and post 12‐week mean scores were found in the intervention group compared to TAU group for the PSRS variable (*p* < 0.05). Future studies should aim to examine whether the social functioning benefits of AE among individuals with schizophrenia‐spectrum disorders are mediated by changes in emotion awareness and regulation. Future studies should aim to replicate the findings with larger samples.
Lal et al. (2023)	Canada Mixed‐methods research design	Horyzons‐Canada (Canadian adaptation of a digital mental health intervention consisting of psychosocial interventions, online social networking and clinical and peer support moderation, *n* = 23) 8 weeks 23 FEP (10M/12F/1B) Outpatients Pre‐intervention 20‐week follow‐up after intervention	26.8 (5.3)	Social Functioning Scale (SFS) Social and Occupational Functional Assessment Scale (SOFAS) Clinical Global Impression Scale (CGI) Scale for the Assessment of Negative Symptoms (SANS) Brief Psychiatric Rating Scale (BPRS) Multidimensional Scale of Perceived Social Support (MSPSS) Calgary Depression Scale (CDS) Strengths Use Efficacy Scale (SUS) Strengths Knowledge Scale (SKS) Self‐Efficacy Scale (SES) Scale for the Assessment of Positive Symptoms (SAPS)	No significant changes from pre‐intervention to 20 week follow‐up for any of the variables. More research is needed with larger sample sizes and using in‐depth qualitative methods to better understand the implementation and impact of Horyzons‐Canada.
Lee et al. (2013)	Australia RCT	Cognitive remediation (*n* = 28) vs. TAU (*n* = 27) 2‐h sessions for a total of 10 weeks 55 FEP (28M/27F) Outpatients Pre‐ and post‐intervention	Cognitive remediation group = 22.88 (4.0) TAU group = 22.74 (4.7)	DSM‐4 Wechsler Test of Adult Reading (WTAR) 17‐item Hamilton Depression Rating Scale (HAMD‐17) Brief Psychiatric Rating Scale (BPRS‐E) Social Functioning Scale (SFS) Trail Making Test (TMT) Longest Digit Span Forward (LDSF) Longest Digit Span Backward (LDSB) Rey Auditory Verbal Learning Test (RAVLT Total) Logical Memory II Percentage Retention (LM) Cambridge Neuropsychological Test Automated Battery (CANTAB) Logical Memory I Paired Associates Learning (PAL) Rapid Visual Processing (RVP)	Statistically significant pre and post‐test mean scores were found in the intervention group compared to the TAU group for the following variables: LDSF (*p* < 0.01) RAVLT Total (*p* < 0.001) SFS (*p* ≤ 0.05) Future studies need to build on the findings in larger samples using blinded allocation and should incorporate longitudinal follow‐up and assessment of potential moderators (e.g. social cognition, self‐efficacy) to examine sustainability and the precise mechanisms of cognitive remediation effects respectively.
Li et al. (2018)	China RCT	Community‐based comprehensive intervention (*n* = 169) vs. control group (*n* = 158) 9 months 384 SZ (197M/187F) Outpatients Pre‐intervention 6‐month follow‐up 9‐month follow‐up	Community‐based comprehensive intervention group = 40.21 (7.57) Control group = 39.70 (7.83)	ICD‐10 Global Assessment of Functioning (GAF) Schizophrenia Quality of Life Scale (SQLS) Internalized Stigma of Mental Illness scale (ISMI) Discrimination and Stigma Scale (DISC‐12) Self‐Efficacy Scale (SES) Brief Psychiatric Rating Scale (BPRS) Positive Negative Syndrome Scale (PANSS)	At 6 months and 9 months, mean scores of DISC‐12 in intervention group were significantly higher than the control group (both *p* < 0.001). At 6 months and 9 months, GAF total scores in intervention group were significantly higher than the control group (both *p* < 0.001). At 6 months and 9 months, there was a significant reduction on BPRS total score in the intervention group when compared with the control group (both *p* < 0.05), after adjustment for pre‐intervention BPRS total scores. At 6 months and 9 months, there was a significant reduction on PANSS‐N total score in the intervention group when compared with the control group (both *p* < 0.05). At 6 months, SES total score in intervention group was significantly lower than the control group (*p* = 0.01). A higher frequency and intensity of the interventions is needed in future research.
Mazzi et al. (2018)	Italy Cross‐sectional	Social point programme (social inclusion interventions, *n* = 27) vs. waiting list group (*n* = 21) 12 months 48 SSD (34M/14F) Outpatients Post‐intervention	Age breakdown not reported	ICD‐10 Global Assessment of Functioning (GAF) Rosenberg Self Esteem scale (RSE) Internalized Stigma of Mental Illness scale (ISMI) WHO Quality of Life Brief Assessment (WHOQOL‐BREF)	Statistically significant post‐intervention mean scores were found in the intervention group compared to the waiting list group for the following variables: GAF (*p* < 0.001) ISMI (*p* = 0.02) Further investigation with larger sample and longitudinal design is warranted to replicate the result of the study.
Minor et al. (2022)	America RCT	Standard metacognitive reflection and insight therapy (MERIT, *n* = 8) vs. tailored MERIT (real‐world interactions were captured via the electronically activated recorder, a smartphone application that passively records audio in daily life, *n* = 12) 24 sessions 20 SZ (13M/7F) Outpatients Pre‐ and post‐intervention	Standard metacognitive reflection and insight therapy group = 45.88 (8.81) Tailored MERIT group = 43.17 (12.43)	Metacognitive Beliefs Questionnaire‐Brief (MCQ‐30) Global Functioning: Social (GF: S) Quality of Life Scale Interpersonal Relations (QLS‐IR) Positive Negative Syndrome Scale (PANSS)	No significant changes from pre and post‐test mean scores were found for any of the variables in both the standard metacognitive reflection and insight therapy group and tailored MERIT group. Future trials with larger samples should test if the strategy of implementing real‐world social interactions to personalise therapy engages metacognition and improves social functioning.
Nahum et al. (2014)	America Pilot study	SocialVille (an online, plasticity‐based training programme that targets social cognition deficits in schizophrenia, *n* = 34) 24 h of online SocialVille game play either from home or at a clinic, over a 6–10‐week period 34 SZ (25M/9F) Outpatients Pre‐ and post‐intervention	(*N* = 17) SZ group = 23.8 (3.2) (*N* = 17) HC group = 23.6 (3.6)	DSM‐4 Global Functioning Scale: Social and Role (GFS) Social Functioning Scale (SFS) Performance‐based Prosody Identification Test (PROID) Mayer‐Salovey‐Caruso Emotional Intelligence Test (MSCEIT) Heinrichs‐Carpenter Quality of Life Scale (QLS) Behavioural Inhibition Scale/Behavioural Activation Scale (BIS/BAS) Temporal Experience of Pleasure Scale (TEPS)	Statistically significant post‐intervention mean scores were found in the intervention group for the following variables: PROID (*p* < 0.01) GFS: Social (*p* < 0.03) TEPS (*p* < 0.04) Future randomised controlled trials are needed to determine whether the findings are replicable.
Nahum et al. (2021)	America A Double‐Blind, randomised, controlled multi‐site clinical trial	SocialVille (an online, plasticity‐based training programme that targets social cognition deficits in schizophrenia, *n* = 76) vs. computer games control (*n* = 71) 40 sessions over 8–12 weeks 147 SZ (102M/45F) Outpatients 16‐week follow‐up Post‐intervention	SocialVille group = 42.5 (13.9) Computer games group = 43.27 (11.5)	DSM‐5 Social Functioning Scale (SFS) Specific Levels of Functioning Scale (SLOF) The Penn Emotional Recognition Test (ER40) Performance‐based Prosody Identification Test (PROID) Penn Faces Memory Test (PFMT) Mayer‐Salovey‐Caruso Emotional Intelligence Test (MSCEIT) UCSD, Performance‐based Skills Assessment second edition (UPSA‐2) Positive Negative Syndrome Scale (PANSS) Virtual Reality Functional Capacity Assessment Tool (VRFCAT) Global Functioning Scale: Social and Role (GFS) Heinrichs‐Carpenter Quality of Life Scale (QLS) The Awareness of Social Inference Test (TASIT) Ambiguous Intentions Hostility Questionnaire (AIHQ) Temporal Experience of Pleasure Scale (TEPS) Behavioural Inhibition Scale/Behavioural Activation Scale (BIS/BAS)	Statistically significant post‐intervention mean scores were found in the intervention group compared to the computer games group for the following variables: SFS (*p* < 0.001) VRFCAT (*p* = 0.03) QLS (*p* = 0.03) TASIT (*p* = 0.01) GFS—Social (*p* = 0.01) The results from the study importantly show that an individualised online intervention may be efficacious in schizophrenia and may serve as an adjunct to psychosocial and/or pharmacological treatments. The study serves as another step in the direction of integrating computerised interventions as part of the therapeutic regime of patients with chronic schizophrenia. Further research is needed to establish the study's findings.
Nijman et al. (2023)	Netherlands Single‐blind RCT	Dynamic interactive social cognition training in virtual reality (*n* = 41) vs. active control group (virtual reality relaxation, *n* = 40) 16 twice a week session 81 SSD (56M/25F) Inpatients and outpatients Pre and post‐intervention 3‐month follow‐up	Dynamic interactive social cognition training in virtual reality group = 39.7 (12.4) Active control group = 35.9 (10.4)	The Mini International Neuropsychiatric Interview Plus The Awareness of Social Inference Test (TASIT) Personal and Social Performance Scale (PSP) Experience Sampling Method diaries (ESM) National Adult Reading Test (NART) Trail Making Test (TMT) Social Interaction Anxiety Scale (SIAS) Positive Negative Syndrome Scale (PANSS) Beck Depression Inventory (BDI) Beck Anxiety Inventory (BAI) Perceived Stress Scale (PSS) Self Esteem Rating Scale (SERS) Simulator Sickness Questionnaire (SSQ) Facial emotion recognition computerised measure (Ekman 60 Faces)	Statistically significant 3‐month follow‐up mean scores were found in the intervention group compared to the active control group for the following variables: Ekman 60 Faces (*p* = 0.02) PSP (*p* = 0.03) TMT‐A (*p* = 0.001) TMT‐B (*p* = 0.01) SIAS (*p* = 0.02) PANSS‐N (*p* = 0.01) ESM‐Positive affect (*p* < 0.001) Further research is needed to strengthen the findings from the study. Additionally, the integration of SCT with other treatments may be necessary, such as CBT, CRT, behavioural activation and/or psychosocial rehabilitation programmes such as Individual Placement and Support. Ultimately, focusing on any single process may be insufficient, as social dysfunction is caused by an interplay of many different factors.
Özer and Dişsiz (2024)	Turkey RCT	Online group‐based acceptance and commitment therapy (ACT, *n* = 26) vs. control group (*n* = 27) 8 sessions, 2 sessions per week for 4 weeks 53 SSD (40M/13F) Outpatients Pre‐ and post‐intervention 3‐month follow‐up	ACT group = 23.26 (3.88) Control group = 23.55 (3.82)	DSM‐5 Positive Negative Syndrome Scale (PANSS) Social Functioning Assessment Scale (SFAS)	Statistically significant pre and post‐test mean scores were found in the intervention group compared to the control group for the following variables: PANSS (*p* < 0.001) SFAS (*p* < 0.001) Statistically significant 3‐month follow‐up mean scores were found in the intervention group compared to the control group for the following variables: PANSS (*p* < 0.001) SFAS (*p* < 0.001) Long follow‐up studies are needed to evaluate ACT programmes' effectiveness in individuals diagnosed with early psychosis.
Pérez‐Aguado et al. (2024)	Spain RCT	Group music Therapy plus TAU (*n* = 30) vs. TAU (*n* = 30) 22 sessions 60 SZ (48M/12F) Outpatients Pre‐ and post‐intervention	Group music Therapy plus TAU group = 36.20 (9.81) TAU group = 42.07 (8.35)	DSM‐5 Positive Negative Syndrome Scale (PANSS) State–Trait Anxiety Inventory (STAI) Calgary Depression Scale for Schizophrenia (CDSS) Internalised Stigma of Mental Illness scale (ISMI) Facial Emotion Discrimination Test (FEDT) Facial Emotion Identification (FEIT) Theory Of Mind (ToM) Social Functioning Scale (SFS) WHO Quality of Life Brief Assessment (WHOQOL‐BREF)	Statistically significant pre and post‐test mean scores were found in the intervention group compared to the TAU group for the following variables: ISMI (*p* < 0.001) SFS (*p* < 0.001) WHOQOL‐BREF (*p* < 0.001) Future multicentre, randomised, controlled trials are needed to strengthen the findings. In addition, it is recommended that future trials are needed to extend to other socio‐cultural contexts in countries where people with mental problems do not have access to MT services.
Priebe et al. (2020)	England RCT	Befriending programme (*n* = 63) vs. active control condition (where they met with unmasked researcher who provided them information about social activities, *n* = 61) 1 year 124 SSD (81M/43F) Outpatients Post‐intervention 6‐month follow‐up	Befriending programme group = 43.4 (10.7) Active control condition group = 41.3 (10.0)	ICD–10 Positive Negative Syndrome Scale (PANSS) Self‐Esteem Rating Scale‐Short Form (SERS‐SF) Clinical Assessment Interview for Negative Symptoms (CAINS) Beck Depression Inventory‐II (BDI‐II) Manchester Short Assessment of Quality of Life (MANSA) Social Outcomes Index (SIX) Time Use Survey (TUS)	Participants in the intervention group had significantly more social contacts (*p* = 0.03) and more favourable SIX scores (*p* = 0.04) compared to the active control group. The analyses comparing the groups at the 6‐month follow‐up showed that patients in the intervention group still had significantly more social contacts (*p* = 0.04) and better scores on the SIX (*p* = 0.03). The findings have implications for both practice and research. In practice, participants and volunteers should be offered programmes with sufficient flexibility to accommodate their varying initial preferences and changes in preferences over time. This may be in regard to the duration of the relationship, the frequency of meetings, the personal motivations of the volunteer etc. For research, the question arises as to whether RCTs are the most appropriate method for evaluating befriending programmes. Trials focus on gains achieved at the end of an intervention period. Yet, in the case of befriending relationships over 12 months, there may be experiences during that period that are not reflected in changed outcome criteria at the 1‐year point, but are still important for patients, perhaps making overall participation worthwhile. Future research may be to collect such data from a large number of befriending programmes currently in practice.
Restek‐Petrović et al. (2014)	Croatia RCT	Psychodynamic group psychotherapy (*n* = 30) vs. TAU (*n* = 30) 2 years 60 SSD (36M/24F) Outpatients Post‐intervention	Psychodynamic group psychotherapy = 39.5 (SD not reported) TAU group = 41.5 (SD not reported)	ICD‐10 Two questionnaires were applied: The self‐assessment instrument and the therapist‐assessment instrument. The questionnaires addressed changes in patient communication in existing and new interpersonal relations, romantic and working functioning and overall social functioning.	The therapists assessed the overall functioning and communication of the majority of patients from both groups as improved, with significantly higher number of patients from psychodynamic group psychotherapy assessed to have improved these areas of social functioning (overall functioning *p* = 0.01; communication *p* = 0.01). Moreover, among patients from psychodynamic group psychotherapy group, a significantly higher number were assessed by the therapists to have unchanged work functioning, while among patients from the TAU group significantly higher number of patients were assessed to have worsened work functioning (*p* < 0.001). The comparison of patient self‐assessment and therapist‐assessments of patients showed that there was a significant difference was found in the area of romantic relations (*p* = 0.04) and overall social functioning (*p* = 0.05). It is necessary to conduct further research to ascertain whether the psychodynamic approach to group psychotherapy truly creates the conditions for psychological change which is reflected in the social sphere by improved functioning.
Ruiz‐Iriondo et al. (2020)	Spain RCT	Integrated psychological therapy and emotional management therapy (*n* = 42) vs. TAU (*n* = 35) 60 group sessions twice a week for 32 weeks 77 SSD (53M/24F) Outpatients Pre‐ and post‐intervention	43.69 (9.03)	DSM‐5 Brief Psychiatric Rating Scale (BPRS) verbal learning (VLT‐I) Wechsler Adult Intelligence Scale‐Third Edition (WAIS‐III) Social Functioning Scale (SFS) Lancashire Quality of Life Profile (LQoLP) Short Assessment of Quality of Life (SCIP)	Statistically significant pre and post‐test mean scores were found in the intervention group compared to the TAU group for the following variables: BPRS (*p* < 0.001) VLT‐I (*p* < 0.001) WAIS‐III (*p* < 0.001) SFS‐Work activities (*p* < 0.001) LQoLP (*p* < 0.001) Further studies are needed to strengthen the findings.
Rus‐Calafell et al. (2013)	Spain RCT	Cognitive‐behavioural social skills (*n* = 13) vs. TAU (*n* = 18) 16 sessions 31 SSD(25M/6F) Outpatients Pre‐ and post‐intervention 6‐month follow‐up	Social skills training group = 37.54 (8.05) TAU group = 42.39 (8.1)	DSM‐5 Positive Negative Syndrome Scale (PANSS) Assertion Inventor (AI) Social Interaction Self‐Statement Test (SISST) Simulated Social Interaction Test (SSIT) Social Functioning Scale (SFS) Health Survey (SF‐36) Screen for Cognitive Impairment in Psychiatry (SCIP)	Statistically significant pre and post‐test mean scores were found in the intervention group compared to the TAU group for the following variables: PANSS (*p* < 0.001) SFS (*p* < 0.001) SISST (*p* < 0.001) AI (*p* < 0.001) SF‐36 (*p* < 0.001) Statistically significant post‐test and 6‐month follow‐up mean scores were found in the intervention group compared to the TAU group for the following variables: PANSS (*p* < 0.001) SFS (*p* < 0.001) SISST (*p* < 0.001) AI (*p* < 0.001) SF‐36 (*p* < 0.001) Future well‐controlled studies should, in one hand, compare the present intervention with other group therapy interventions with different objectives from the social skills training and, on another hand, determine the contribution of each component of the SST to the participant's functioning improvement.
Rus‐Calafell et al. (2014)	Spain Repeated measures experimental design	Virtual reality‐integrated programme for improving social skills (*n* = 12) 16 sessions 12 SSD (7M/5F) Outpatients Pre‐ and post‐intervention 4‐month follow‐up	36.50 (6.01)	DSM‐4 Positive Negative Syndrome Scale (PANSS) Assertion Inventor (AI) Simulated Social Interaction Test (SSIT) Social Avoidance and Distress Scale (SADS) Social Functioning Scale (SFS)	Statistically significant pre and post‐test mean scores were found in the intervention group: PANSS (*p* < 0.001) SSIT (*p* < 0.001) SADS (*p* < 0.001) SFS (*p* = 0.02) Statistically significant post‐test and 4‐month follow‐up mean scores were found in the intervention group: PANSS (*p* < 0.001) SSIT (*p* < 0.001) SADS (*p* < 0.001) SFS (*p* < 0.05) A large, randomised controlled trial is needed to establish the efficacy of the SST with the VR‐integrated programme compared with other well‐established interventions for social skill impairments.
Sarandöl et al. (2024)	Turkey RCT	Art therapy (*n* = 7) vs. psychosocial skills training (*n* = 8) 90‐min sessions once a week for 17 weeks 15 SZ (11M/4F) Outpatients Pre‐intervention 3‐month follow‐up 6‐month follow‐up 12‐month follow‐up	Art therapy group = 44 (6.92) Psychosocial skills training group = 41.62 (7.28)	DSM‐4 Positive Negative Syndrome Scale (PANSS) Calgary Depression Scale for Schizophrenia (CDSS) Social Functioning Scale (SFS) Beck Depression Inventory (BDI) Beck Anxiety Inventory (BAI) Zarit Burden Interview (ZBI) Unexpected Outcomes Test (UOT)	In the art therapy group, there were positive changes in PANSS negative symptoms (*p* = 0.001) from the 3rd month, and there were significant changes in passive withdrawal (*p* = 0.001), abstract thinking difficulty (*p* = 0.001), loss of spontaneity (*p* = 0.001) and flow of conversation (*p* = 0.001). SFS evaluation showed a significant difference in the social withdrawal subscale (*p* = 0.001) between the months starting from the 3rd month, as well as in pro‐social activities (*p* < 0.001) and independence level (*p* = 0.03) subscales between the pre‐intervention and 12‐month scores. In psychosocial skills training group, there was a significant change in the PANSS general psychopathology score between all evaluated months and the 12th month (*p* < 0.001), as well as in the independence level subscale of the SFS when the results from pre‐intervention, 6 months and 12 months were compared (*p* < 0.001). Only the abstract thinking difficulty subscale of the negative symptoms of the PANSS scale showed a significant difference (*p* = 0.01). Studies with larger samples and involving multiple healthcare centres are needed.
Şenormancı et al. (2021)	Turkey RCT	Exercise intervention (*n* = 20) vs. control group (*n* = 19) 2 days a week, 1 h for 3 months 39 SZ (24M/15F) Inpatients Pre‐ and post‐intervention	Exercise group = 40.5 (SD not reported) Control group = 43 (SD not reported)	DSM‐5 Scale for the Assessment of Positive Symptoms (SAPS) Scale for the Assessment of Negative Symptoms (SANS) Calgary Depression Scale (CDS) Schedule for Assessment of Insight (SAI) Resilience Scale for Adults (RSA) Functional Remission of General Schizophrenia (FROGS)	In both groups significant decrease in SANS scores (intervention group *p* < 0.001, control group *p* = 0.04), significant increase in scores of FROGS total (intervention group *p* = 0.001, control group *p* < 0.001) and social functioning (intervention group *p* = 0.001, control group *p* = 0.04), daily life skills subscale (intervention group *p* = 0.02, control group *p* < 0.001) and RSA perception of the self (intervention group *p* < 0.001, control group *p* < 0.001)were observed. In the exercise group, significant decrease in CDS scores (*p* < 0.001) and significant increase in SAI awareness of illness (*p* = 0.02), FROGS health and treatment (*p* = 0.02), occupational functioning scores (*p* = 0.04) were found. Long‐term studies with larger sample sizes, conducted in multiple nursing home settings and also investigating the neurobiological effects of exercise, are needed.
Shen et al. (2022)	China Preliminary efficacy	VR‐based social cognition and interaction training plus TAU (*n* = 28) vs. traditional social cognition and interaction training plus TAU (*n* = 30) vs. waiting groups plus TAU (*n* = 29) 10 sessions over 3 weeks 76 SSD (50M/26F) Outpatients Pre‐ and post‐intervention	VR‐based social cognition and interaction training group = 30.81 (4.90) Traditional social cognition and interaction training group = 31.00 (7.48) Waiting list group = 30.74 (6.59)	DSM‐4 Chinese version of Social Cognition Screening Questionnaire (C‐SCSQ) Theory Of Mind (ToM) Hostile attributional Bias (HB) Metacognition (MC) Personal and Social Performance Scale (PSP) Chinese version of the Face‐Affective Identification Task (C‐FAIT) The Digit Span Test (DST) Verbal Fluency Test (VFT)	Both VR‐based social cognition and interaction and traditional social cognition and interaction groups had a significantly higher scores than the waiting‐list group post‐intervention for the following variables: C‐FAIT (*p* < 0.001) (*p* = 0.04) MC (*p* < 0.001) (*p* = 0.02) PSP (*p* < 0.001) (*p* = 0.01) The VR‐based social cognition and interaction group had a significantly higher post‐intervention C‐FAIT score than the TR‐SCIT group (*p* = 0.03). VR‐based social cognition and interaction group had a significantly lower HB score than the waiting‐list group at post‐intervention (*p* = 0.05). The VR‐based social cognition and interaction group had a significantly higher post‐intervention MC score than the traditional social cognition and interaction group (*p* = 0.04). Further multicentre, large‐sample, long‐term follow‐up randomised controlled studies are needed in the future.
Shih and Yang (2023)	Taiwan Longitudinal, single‐blind experimental study	Animal‐assisted therapy (*n* = 45) vs. control group (engaged in routine discussion groups and watched short films about animals, *n* = 45) 1 h/week for 12 weeks 90 SZ (45M/45F) Inpatients Pre‐ and post‐intervention 3‐month follow‐up	Animal‐assisted therapy group = 49.5 (9.5) Control group = 51.0 (9.7)	DSM‐5 Social Adaptive Function Scale (SAFS) Mental Health‐Social Functioning Scale (MHSFS) WHO Quality of Life Brief Assessment (WHOQOL‐BREF)	The experimental group, the MHSFS scores of participants were significantly higher at post‐intervention (*p* < 0.01). Participants in the control group had significantly higher MHSFS scores at post‐intervention (*p* < 0.05). The MHSFS scores of the experimental group decreased significantly at 3‐month follow‐up (*p* < 0.05). In the experimental group, the SAFS scores of participants at post‐intervention were significantly lower than those at pre‐intervention (*p* < 0.01). The control group had significantly lower SAFS scores at post‐intervention and 3‐month follow‐up than at pre‐intervention (*p* < 0.05). The WHOQOL‐BREF scores of the experimental group at post‐intervention and 3‐month follow‐up were significantly higher than their scores at pre‐intervention (both *p* < 0.01). The effect of intervention is easily influenced by the environment of the institutions; therefore, future studies should use the random assignment methods.
Shimada et al. (2016)	Japan Quasi‐experimental controlled trial using a non‐randomised method	Individualised occupational therapy plus group occupational therapy (*n* = 30) vs. group occupational therapy (*n* = 21) 1–2 h at a time, 3–5 times per week 51 SSD (26M/25F) Inpatients Discharge or 3 months following hospitalisation	Individualised occupational therapy plus group occupational therapy group = 40.80 (10.89) Group occupational therapy group = 43.67 (8.77)	DSM‐4 Global Assessment of Functioning (GAF) Brief Assessment of Cognition in Schizophrenia‐Japanese version (BACS‐J) Positive Negative Syndrome Scale (PANSS)	Participants in the individualised occupational therapy plus group occupational therapy group had demonstrated significant improvements in BACS‐J scores compared to group occupational therapy only for the following variables: Verbal memory (*p* < 0.01), working memory (*p* < 0.01), verbal fluency (*p* < 0.01), attention (*p* < 0.01), executive function (*p* < 0.01), BACS‐J composite score (*p* < 0.01). In addition, significant improvements were found for general psychopathology subscale (*p* < 0.05), positive subscale (*p* < 0.01) and total PANSS score (*p* < 0.01). Additional study is needed to replicate and extend the effects of individualised occupational therapy.
Shimada et al. (2018)	Japan Multicentre, open‐labelled, blinded‐endpoint RCT	Individualised occupational therapy plus group occupational therapy (*n* = 66) vs. group occupational therapy (*n* = 63) 1–2 h at a time, 3–5 times per week 136 SSD (67M/69F) Inpatients Discharge or 3 months following hospitalisation	Individualised occupational therapy plus group occupational therapy group = 41.39 (11.03) Group occupational therapy group = 43.39 (9.97)	DSM‐5 Brief Assessment of Cognition in Schizophrenia‐Japanese version (BACS‐J) Schizophrenia Cognition Rating Scale Japanese version (SCoRS‐J) Social Functioning Scale Japanese version (SFS‐J) Global Assessment of Functioning (GAF) The Intrinsic Motivation Inventory Japanese version (IMI‐J) Morisky Medication Adherence Scale‐8 (MMAS‐8) Positive and Negative Syndrome Scale (PANAS) Client Satisfaction Questionnaire‐8 (CSQ‐8J)	Results of linear mixed effects models indicated that the individualised occupational therapy plus group occupational therapy group showed significant improvements in verbal memory (*p* < 0.01), working memory (*p* = 0.02), verbal fluency (*p* < 0.01), attention (*p* < 0.01) and composite score (*p* < 0.01)) on the BACS‐J; interest/enjoyment (*p* < 0.01), value/usefulness (*p* < 0.01), perceived choice (*p* < 0.01) and IMI‐J total (*p* < 0.01) on the IMI‐J; MMAS‐8 score (*p* < 0.01) compared with the group occupational therapy alone. Patients in the individualised occupational therapy plus group occupational therapy group demonstrated significant improvements on the CSQ‐8J compared with the group occupational therapy alone (*p* < 0.01). Future study is needed to address the prognostic significance of individualised occupational therapy by exploring the impact of individualised occupational therapy and clinical variables on functional outcome.
Shimada et al. (2022)	Japan RCT	Individualised occupational therapy plus group occupational therapy (*n* = 48) vs. group occupational therapy (*n* = 54) follow‐up study from (Shimada et al. 2016) 102 SSD (54M/48F) Inpatients 5‐year follow‐up	Individualised occupational therapy plus group occupational therapy group = 40.58 (10.53) Group occupational therapy group = 44.30 (10.33)	DSM‐5 Social Functioning Scale (SFS) Brief Assessment of Cognition in Schizophrenia (BACS) Schizophrenia Cognition Rating Scale (SCoRS) Intrinsic Motivation Inventory (IMI) Global Assessment of Functioning (GAF) Positive Negative Syndrome Scale (PANSS) Client Satisfaction Questionnaire‐8 (CSQ‐8)	Significant differences were detected for the assessment scores at post assessment for verbal memory (*p* < 0.01), executive function (*p* = 0.03) and composite score in BACS (*p* < 0.01); patient global rating in SCoRS (*p* = 0.03); interest/enjoyment (*p* < 0.01), perceived choice (*p* < 0.01), value/usefulness (*p* < 0.01) and total in IMI (*p* < 0.01); positive (*p* < 0.01), general psychopathology (*p* = 0.01) and total in PANSS (*p* < 0.01); GAF (*p* = 0.04); and CSQ‐8 (*p* < 0.01) in the comparisons between GOT + IOT and GOT alone groups. Significant changes in withdrawal/social engagement (*p* < 0.001) interpersonal communication (*p* < 0.001), pro‐social activities (*p* < 0.001), recreation (*p* < 0.001), independence‐competence (*p* = 0.02) and total score (*p* < 0.001) in SFS from pre‐intervention to follow‐up, in the individualised occupational therapy plus group occupational therapy group compared with the group occupational therapy only. A key clinical implication: Adding individualised occupational therapy, an individualised and goal‐oriented intervention, to treatment as usual during hospitalisation may improve social functioning 5 years after discharge. The findings should be considered preliminary until replicated by future studies.
Štrkalj‐Ivezić et al. (2013)	Croatia RCT	Rehabilitation day centre programme (*n* = 50) vs. waiting list (*n* = 48) 6 months 98 SZ (51M/47F) Outpatients Pre‐ and post‐intervention	Rehabilitation day centre programme group = 39.6 (SD not reported) Waiting list group = 39.9 (SD not reported)	ICD‐10 Occupational Self‐Assessment (OSA) Manchester Short Assessment of Quality of Life (MANSA) Rosenberg Self Esteem scale (RSE)	Statistically significant pre and post‐test mean scores were found in the intervention group compared to the waiting‐list group for the following variables: OSA (*p* < 0.001) MANSA (*p* < 0.001) RSE (*p* < 0.001) Additional study is needed to replicate and extend the effects of the programme.
Tong et al. (2021)	China Preliminary efficacy	Group art therapy programme (*n* = 53) vs. TAU (*n* = 51) 30 sessions 104 SZ (53M/51F) Inpatients Pre‐ and post‐intervention	Group art therapy group = 43.68 (7.25) TAU group = 46.06 (8.26)	DSM‐5 General Self‐Efficacy Scale (GSES) Scale of Social Skills for Psychiatric Inpatients (SSPI)	Statistically significant pre and post‐test mean scores were found in the intervention group compared to the TAU group for the following variables: GSES (*p* = 0.01) SSPI (*p* = 0.01) Further work must include research samples using common materials to explore the effects of group art therapy on those from different cultural backgrounds.
Torres et al. (2002)	Spain RCT	Group 1: El Tren (board game), social skills training, psychomotor skills training and occupational therapy, *n* = 19) vs. Group 2: Social skills training, psychomotor skills training and occupational therapy (*n* = 16) vs. Group 3: Occupational therapy only (*n* = 14) 6 months 49 SZ (36M/13F) Inpatients Pre‐ and post‐intervention	Age breakdown not reported	DSM‐4 Social Functioning Scale (SFS)	The participants in Group 1 demonstrated significantly greater improvement in interpersonal functioning than the participants in the other two groups (*p* < 0.001 for Group 1 versus Group 3, *p* < 0.001) and significantly greater improvement in social withdrawal than the participants who were assigned to Group 3 (*p* < 0.001) but not those who were assigned to Group 2. El Tren has been tested at only one centre; therefore, the new resource needs to be tested in similar centres around the world to cross‐validate the results of the study.
Varga et al. (2018)	Hungary RCT	Case management (*n* = 26) vs. community‐based club (*n* = 26) vs. TAU (*n* = 23) 6 months 75 SZ (38M/37F) Outpatients Pre‐ and post‐intervention	Case management group = 37.5 (9.4) Community‐based club group = 39.6 (8.4) TAU group = 39.9 (8.8)	DSM‐5 Global Assessment of Functioning (GAF) Positive Negative Syndrome Scale (PANSS) Social Cognition Analyser ApplicatioN (SCAN)	A significant between‐group differences in GAF scores at pre‐intervention (*p* = 0.01) as well as after 6 months (*p* < 0.01). In addition, compared to pre‐intervention GAF scores increased significantly after 6 months both in the community‐based club group (*p* < 0.01) and in the case management group (*p* < 0.01). In the within‐group analysis of the summary SCAN scores the community‐based club group was found to have increased their performance significantly (*p* < 0.01), while in the between‐ group analysis significant differences were found between the three groups at 6 months (*p* = 0.04). A significant positive correlation between changes in GAF scores and changes in summary social cognition scores during the 6‐month‐long treatment period in the community‐based club group (*p* = 0.04) as well as in the case management group (*p* = 0.03). The study did not assess potential relations between neurocognitive factors, social cognition, functional outcomes, clinical symptoms and vocational status. Future studies should address the effects between these factors. In addition, other aspects of social cognition such as attributional style as well as social perception should be reflected in future measures.
Veltro et al. (2011)	Italy RCT	Cognitive‐emotional rehabilitation (*n* = 12) vs. problem‐solving training (*n* = 12) Weekly sessions for 12 months 24 SZ (gender distribution not reported) Outpatients Pre‐ and post‐intervention	Cognitive‐emotional rehabilitation group = 38.8 (6.3) Problem‐solving training = 37.7 (11.16)	DSM‐4 SCID‐1 Social Functioning Scale (SFS) Positive Negative Syndrome Scale (PANSS) Social and Occupational Functional Assessment Scale (SOFAS) Trail Making Test A (TMT‐A) Rey Auditory Verbal Learning Test (RAVLT) Trail Making Test B (TMT‐B) Memory Theory Of Mind (TOM) Emotion Recognition	Both training methods were found to be effective in psychopathological measures (*p* < 0.01) and in social functioning (*p* < 0.05). On cognitive function improvements were specific to the rehabilitative approach (*p* < 0.05). Problem‐solving training group mainly improved capacities for planning and memory (*p* < 0.01), while the cognitive‐emotional rehabilitation group improved measures such as social cognition (*p* < 0.01), TOM (*p* < 0.01) and emotion recognition (*p* < 0.01). Future research, is desirable to study the effectiveness of such treatments also in combination, in order to broadly treat cognitive disorders representing the core symptoms of the disease.
Ventura et al. (2019)	America RCT	Cognitive remediation (*n* = 39) vs. healthy behaviours training (*n* = 41) 50 sessions over 6 months and then booster sessions over the next 6 months 80 SSD (62M/18F) Outpatients 12‐month follow‐up	Cognitive remediation group = 21.5 (3.0) Healthy behaviours training group = 21 (4.0)	DSM‐4 Scale for the Assessment of Negative Symptoms (SANS) Brief Psychiatric Rating Scale (BPRS) The UCLA Social Attainment Survey (SAS)	Improvements over 12 months were found favouring cognitive remediation for SANS Expressive Symptoms (*p* < 0.01), which was composed of Affective Flattening (*p* < 0.01) and Alogia (*p* = 0.04), and for SANS Experiential Symptoms, composed of Avolition/Apathy (*p* = 0.04) and Anhedonia/Asociality (*p* < 0.01). Cognitive remediation was associated with improvements in social functioning (*p* = 0.05) as compared to healthy behaviours training. Future research should explore whether improvements in negative symptoms and social functioning are mediated by improvements in facilitator contact or cognition.
Wong et al. (2024)	Hong Kong Single‐blind RCT	Strength‐based cognitive‐behavioural therapy (*n* = 42) vs. peer‐to‐peer support (*n* = 44) vs. TAU (*n* = 41) 1 year 127 SSD (51M/76F) Outpatients Pre‐intervention 6‐month follow‐up (mid‐intervention) Post‐intervention 18‐month follow‐up (6‐month follow‐up)	Age breakdown not reported	DSM‐5 Social Functioning Questionnaire (SFQ) Mental Health Recovery Measure (MHRM) The Trait Hope Scale Quality of Life Scale (QoL) Defeatist performance belief subscale of the Dysfunctional Attitude Scale (DAS) Recovery Assessment Scale (RSAS) Medical Outcomes Study Social Support Survey (MOS‐SSS)	After strength‐based cognitive‐behavioural therapy intervention, significant improvements were recorded for SFQ (*p* < 0.05) and social support (*p* < 0.05) and RSAS worsened (*p* < 0.05). Among these effects, only the improvements of SFQ (*p* < 0.05) and social support (*p* < 0.01) remained significant at 6‐month follow‐up. For the peer‐to‐peer support intervention, significant improvements were found in social QoL (*p* < 0.05) and DAS (*p* < 0.05). At 6‐month follow‐up, there were significant improvements in physical QoL (*p* < 0.05). The study provides some initial evidence for the need to explore further the specific linkage between two major lines of research and intervention (the hope‐motivation‐strength pathway and the defeatist‐asocial belief pathway) for people recovering from schizophrenia. Further clarity about the inter‐relationship between the various factors in these two lines of inquiry would yield great theoretical and practical implications.
Yildiz et al. (2004)	Turkey RCT	Psychosocial skills training (*n* = 15) vs. TAU (*n* = 15) Weekly 90 min in two sessions for 8 months 30 SZ (18M/12F) Outpatients Pre‐ and post‐intervention	Psychosocial skills training group = 33.53 (10.47) TAU group = 29.80 (4.93)	DSM‐4 Social Functioning Scale (SFS) Global Assessment of Functioning (GAF) Heinrichs‐Carpenter Quality of Life Scale (QLS) Positive Negative Syndrome Scale (PANSS)	Statistically significant pre and post‐test mean scores were found in the intervention group compared to the TAU group for the following variables: SFS (*p* < 0.01) GAF (*p* < 0.01) QLS (*p* < 0.01) PANSS (*p* = 0.02) Whether treatment gains continue for a long time should be tested for this programme in another study. The study needs to be replicated with larger samples.
Yildiz et al. (2018)	Turkey RCT	Psychosocial skills group training (*n* = 10) vs. metacognitive training group 40 sessions, once a week in two sessions, each lasting 40–50 min 20 SSD (13M/7F) Outpatients Pre‐ and post‐intervention	Psychosocial Skills Training group = 37.4 (10.7) Metacognitive training group = 33.1 (4.6)	DSM‐4 Global Assessment of Functioning (GAF) Positive Negative Syndrome Scale (PANSS) CGI‐S, Clinical Global Impression‐Severity (CGI‐S) Heinrichs‐Carpenter Quality of Life Scale (QLS) Cognitive Assessment Interview (CAI)	Statistically significant pre and post‐test mean scores were found in the psychosocial skills group training for the following variables: PANSS (*p* < 0.01) GAF (*p* < 0.01) CAI (*p* < 0.01) QLS (*p* < 0.01) Statistically significant pre and post‐test mean scores were found in the metacognitive training group for the following variables: PANSS (*p* = 0.04) CAI (*p* < 0.01) QLS (*p* = 0.03) Improvement in cognitive and social functioning should be tested by neurocognitive tests in the large‐scale studies with control groups.
Zhu et al. (2020)	China Longitudinal, multicentre RCT	Computerised cognitive remediation therapy (*n* = 78) vs. TAU (*n* = 79) 12 weeks with 4–5 sessions per week 157 SZ (85M/72F) Outpatients Pre‐ and post‐intervention 6‐month follow‐up	Computerised cognitive remediation therapy group = 43.74 (9.24) TAU group = 43.65 (8.64)	DSM‐4 MATRICS Consensus Cognitive Battery (MCCB) Hopkins Verbal Learning Test‐Revised (HVLT‐R) Brief Visuospatial Memory Test‐Revised (BVMT‐R) Mayer‐Salovey‐Caruso Emotional Intelligence Test (MSCEIT) Positive Negative Syndrome Scale (PANSS) UCSD Performance‐Based Skills Assessment (UPSA) Personal and Social Performance Scale (PSP)	Statistically significant pre and post‐test mean scores were found in the intervention group compared to the TAU group for the following variables: PSP (*p* = 0.01) MSCEIT (*p* = 0.04) MCCB (*p* = 0.05) Further studies are needed with younger patients with schizophrenia, longer follow‐up periods and more active control groups to more clearly demonstrate the cognition and functional benefits of this form of remediation.
Zimmer et al. (2007)	Brazil RCT	Integrated psychological therapy programme (*n* = 20) vs. TAU (*n* = 36) 12 weekly sessions 56 SSD (42M/14F) Outpatients Pre‐ and post‐intervention	Integrated psychological therapy group = 36.05 (7.09) TAU group =39.31 (8.85)	ICD‐10 Global Assessment of Functioning (GAF) Social and Occupational Functional Assessment Scale (SOFAS) Social Anxiety Scale (SAS) Mini‐Mental State Examination (MMSE) Word‐Span WHO Quality of Life Brief Assessment (WHOQOL‐BREF) Operational Criteria Checklist for Psychotic Illness (OPCRIT)	Statistically significant pre and post‐test mean scores were found in the intervention group compared to the TAU group for the following variables: MMSE (*p* = 0.05) SAS (*p* = 0.03) GAF (*p* < 0.01) SOFAS (*p* = 0.01) WHOQOL‐BREF (*p* = 0.02) Studies involving larger samples, longer follow‐up periods and additional outcome measures are needed in order to assess the specific effects on dimensions of social functioning, cognitive functioning and quality of life in patients with schizophrenia.

Abbreviations: CHR, clinical high risk; FEP, first episode of psychosis; DSM‐3/4/5, *Diagnostic and Statistical Manual of Mental* Disorders third/fourth/fifth editions; ICD‐10, *International Classification of Diseases* 10th edition; RCT, randomised control trial; SCID‐1/2/4/5, Structured Clinical Interview for DSM Disorders first/s/fourth/fifth edition; SSD, schizophrenia spectrum disorder; SZ, schizophrenia.

### 3.1 Risk of Bias and Certainty Assessment

According to the EPHPP tool, the global quality rating for the included intervention studies were as follows: ‘strong’ (*n* = 8), ‘moderate’ (*n* = 52) and ‘weak’ (*n* = 4) (see Table 1). Additionally, the one included qualitative study scored a global rating of ‘strong’ according to the JBI tool (see Table 2). Moreover, according to the EPHPP tool, the global quality ratings for the studies examining factors associated with high or low social functioning in psychosis were as follows: ‘strong’ (*n* = 2), ‘moderate’ (*n* = 29) and ‘weak’ (*n* = 1) (see Table 4). This suggests that the majority of the studies included in the present review included studies with low risk of bias.

**TABLE 4:** Quality assessment for quantitative studies—EPHPP tool—for factors associated with high or low social functioning in psychosis.

Author (year)	Selection bias	Study design	Confounders	Data collection methods	Withdrawals and dropouts	Analyses (appropriateness)	Global rating
Addington et al. (2006)	Moderate	Strong	Moderate	Strong	Strong	Yes	Moderate
Bright et al. (2018)	Moderate	Moderate	Strong	Moderate	Moderate	Yes	Moderate
Chang et al. (2017)	Moderate	Strong	Moderate	Moderate	Weak	Yes	Moderate
Chudleigh et al. (2011)	Moderate	Weak	Moderate	Strong	Strong	Yes	Moderate
Clay et al. (2021)	Moderate	Moderate	Moderate	Strong	Strong	Yes	Moderate
Corcoran et al. (2011)	Moderate	Moderate	Moderate	Moderate	Strong	Yes	Moderate
Favrod et al. (2019)	Moderate	Moderate	Moderate	Moderate	Strong	Yes	Moderate
García‐López et al. (2022)	Moderate	Moderate	Moderate	Strong	Strong	Yes	Moderate
González‐Blanch et al. (2020)	Moderate	Strong	Moderate	Strong	Moderate	Yes	Moderate
González‐Blanch et al. (2022)	Moderate	Weak	Moderate	Strong	Weak	Yes	Moderate
Granholm et al. (2013)	Moderate	Moderate	Moderate	Strong	Moderate	Yes	Moderate
Grant and Beck (2009)	Moderate	Moderate	Moderate	Moderate	Moderate	Yes	Moderate
Grove et al. (2016)	Moderate	Moderate	Moderate	Moderate	Moderate	Yes	Moderate
Henry et al. (2008)	Strong	Moderate	Moderate	Moderate	Moderate	Yes	Moderate
Hill and Startup (2013)	Moderate	Moderate	Moderate	Moderate	Weak	Yes	Moderate
Jalbrzikowski et al. (2014)	Moderate	Strong	Moderate	Moderate	Strong	Yes	Moderate
Kimhy et al. (2012)	Moderate	Moderate	Moderate	Moderate	Weak	Yes	Moderate
Kimhy et al. (2016)	Moderate	Moderate	Moderate	Moderate	Weak	Yes	Moderate
Lysaker et al. (2007)	Moderate	Moderate	Moderate	Strong	Moderate	Yes	Moderate
Moe et al. (2021)	Moderate	Moderate	Moderate	Moderate	Moderate	Yes	Moderate
Patton et al. (2022)	Moderate	Moderate	Moderate	Moderate	Moderate	Yes	Moderate
Pennou et al. (2021)	Moderate	Moderate	Moderate	Moderate	Moderate	Yes	Moderate
Perry et al. (2011)	Moderate	Moderate	Moderate	Moderate	Moderate	Yes	Moderate
Phalen et al. (2017)	Moderate	Moderate	Moderate	Moderate	Weak	Yes	Moderate
Romm et al. (2012)	Moderate	Moderate	Moderate	Strong	Moderate	Yes	Strong
Schlosser et al. (2015)	Moderate	Moderate	Moderate	Strong	Moderate	Yes	Strong
Sullivan et al. (2013)	Weak	Moderate	Strong	Moderate	Weak	Yes	Moderate
Sullivan et al. (2014)	Moderate	Strong	Moderate	Strong	Weak	Yes	Moderate
Taylor and Harper (2017)	Moderate	Weak	Weak	Moderate	Moderate	Yes	Weak
Vines et al. (2022)	Moderate	Moderate	Weak	Moderate	Moderate	Yes	Moderate
Voges and Addington (2005)	Moderate	Moderate	Weak	Strong	Moderate	Yes	Moderate
Woolverton et al. (2018)	Moderate	Moderate	Weak	Moderate	Moderate	Yes	Moderate

Abbreviation: EPHPP, Effective Public Health Practice Project (Thomas et al. 2004).

### 3.2 Physical Exercise Interventions

Five studies examined physical exercise interventions (Dubreucq et al. 2020; Firth et al. 2018; Kern et al. 2020; Kimhy et al. 2021; Şenormancı et al. 2021). Four studies found statistically significant improvements in social functioning in psychosis compared to the treatment‐as‐usual (TAU) group (Dubreucq et al. 2020; Firth et al. 2018; Kimhy et al. 2021; Şenormancı et al. 2021). Statistically significant improvements following the intervention included reduced positive and negative symptoms of psychosis, hostile and cognitive biases and depressive symptoms and increased social cognition, cardiorespiratory fitness, BMI, resilience and insight (Dubreucq et al. 2020; Firth et al. 2018; Kern et al. 2020; Şenormancı et al. 2021). There were also statistically non‐significant improvements in social interaction anxiety, quality of life (QoL), waist circumference, BDNF and occupational functioning (Firth et al. 2018; Kern et al. 2020; Şenormancı et al. 2021). Interventions that demonstrated statistically significant social functioning outcomes ranged from 10 weeks to 3 months with 60–120 min of weekly sessions (Dubreucq et al. 2020; Firth et al. 2018; Kimhy et al. 2021; Şenormancı et al. 2021). Alternatively, sessions under 40 min exerted no significant effects (Kern et al. 2020), suggesting that session duration could be critical to effectiveness. The negative findings might also be attributed to the sample having a higher mean age (55) (Kern et al. 2020), compared to the younger cohorts (age range 25–40) who appeared to benefit more from this type of intervention (Dubreucq et al. 2020; Firth et al. 2018; Kimhy et al. 2021; Şenormancı et al. 2021). Among the five physical exercise studies, heterogeneity was observed across the included samples. For example, the one FEP study (mean age = 25.85) was characterised by more vigorous and intense exercise regimes (Firth et al. 2018), whereas the two schizophrenia studies (mean age = 33–43) engaged in low‐intensity exercises such as brisk walking or resistance band exercises (Dubreucq et al. 2020; Şenormancı et al. 2021). This highlights that the type of exercise intervention delivered may need to be adapted based on the clients' age in order for it to be beneficial.

### 3.3 Art Interventions

Three studies examined art interventions (Pérez‐Aguado et al. 2024; Sarandöl et al. 2024; Tong et al. 2021). All studies found statistically significant improvements in social functioning in psychosis compared to TAU (Pérez‐Aguado et al. 2024; Sarandöl et al. 2024; Tong et al. 2021). Statistically significant improvements following the intervention also included reduced positive and negative symptoms, increased self‐efficacy, reduced stigma and improved QoL (Pérez‐Aguado et al. 2024; Sarandöl et al. 2024; Tong et al. 2021). There were also statistically non‐significant improvements found for self‐esteem, relationship building, social and emotional withdrawal, and communication skills (Pérez‐Aguado et al. 2024; Sarandöl et al. 2024; Tong et al. 2021). Art interventions that demonstrated statistically significant social functioning outcomes included group art therapy (painting, music, literature, theatre and clay) to enhance social skills (Sarandöl et al. 2024); music therapy, focusing on songwriting and instrument play to aid emotional regulation and social interaction (Pérez‐Aguado et al. 2024); and creative activities to improve self‐expression and confidence (Tong et al. 2021). All three studies, employed structured group‐based art interventions, facilitated by trained art therapists, with sessions ranging from 60 to 90 min over 12–22 weeks (Pérez‐Aguado et al. 2024; Sarandöl et al. 2024; Tong et al. 2021). These shared features highlight the importance of structured, therapist‐guided interventions to achieve meaningful clinical outcomes. As these studies included schizophrenia only samples, it is unclear whether these findings are applicable to CHR or FEP populations and therefore highlights avenues for future research.

### 3.4 Social Recovery Therapy

Social recovery therapy (SRT) is a CBT intervention designed to enhance positive beliefs, reduce avoidance and increase social activity (Fowler et al. 2018, 2019). Two studies found statistically significant improvements in social functioning in FEP compared to TAU (Fowler et al. 2018, 2019). Statistically significant improvements following the intervention also included reduced positive and negative symptoms of psychosis, social anxiety and hopelessness (Fowler et al. 2018). There were also statistically non‐significant improvements found for depression; sense of agency and optimism; and engagement in paid work, education and voluntary work (Fowler et al. 2018, 2019). A qualitative study found that SRT therapists who encouraged self‐understanding, supported clients to confront their fears and helped them to achieve meaningful goals played an important role in fostering self‐agency, hope and optimism, which have been deemed essential for social recovery (Gee et al. 2023). This qualitative study employed reflexive thematic analysis with a combined deductive–inductive coding process (Gee et al. 2023). A coding framework based on the SRT adherence checklist, which included components such as engagement, assessment and cognitive strategies, was developed and refined after coding eight transcripts (Gee et al. 2023). An inductive process then followed whereby new codes were added to capture unidentified relevant themes. Each transcript was then independently coded by at least two researchers to ensure consistency, and regular meetings were held to discuss discrepancies and to reach consensus on the themes and interpretations (Gee et al. 2023). This multi‐phase analysis entailed thoroughly examining the clients' experiences and identifying theory‐driven and novel themes (Gee et al. 2023).

In terms of SRT outcomes, at the 15‐month follow‐up, 25% of the SRT + TAU group with non‐affective psychosis engaged in paid work, compared to none in the TAU group. Additionally, no statistically significant differences were found between the SRT + TAU group versus TAU group for the affective psychosis group regarding paid work (44.4% vs. 46.2%, respectively) (Fowler et al. 2019). Over 40% of non‐affective psychosis participants engaged in education or voluntary work, regardless of treatment (Fowler et al. 2019). The authors therefore suggested that SRT appears to benefit individuals with non‐affective psychosis more than affective psychosis. However, the small affective psychosis sample (*n* = 22) compared to the non‐affective psychosis sample (*n* = 44) may have been potentially underpowered to detect statistically significant between group effects (Fowler et al. 2019). It is also important to note that both SRT studies lacked a matched control intervention group, which would clarify whether the observed improvements were specific to SRT or due to non‐specific factors pertaining to social interaction or general therapeutic support. Without this control, it is difficult to determine whether the benefits stemmed from SRT's unique components (e.g., behavioural experiments, goal‐oriented formulation) or was due to the general engagement with mental health services (Fowler et al. 2018, 2019).

### 3.5 Metacognitive Interventions

Two studies examined metacognitive interventions in schizophrenia samples (*n* = 20–24 patients) (Han and Lee 2022; Minor et al. 2022). This intervention targeted metacognitive beliefs to increase self‐awareness and understanding of others, reduce frustration and increase social interactions (Han and Lee 2022). Both studies employed structured, manualised interventions delivered by trained therapists who received specialised supervision to ensure consistency across protocols (Han and Lee 2022; Minor et al. 2022). However, these studies differed in their structure and strategies employed. One study implemented a 10‐week programme, consisting of weekly sessions designed to specifically address delusions and auditory hallucinations in addition to enhancing one's understanding of others and social situations (Han and Lee 2022). This study found statistically significant improvements in social functioning, social activity levels and social interactions and reduced auditory hallucinations and delusions in psychosis compared to TAU (Han and Lee 2022). In contrast, the second study implemented a 24‐week intervention that employed audio recordings of real‐world interactions to target metacognitive beliefs (Minor et al. 2022). However, this study did not find statistically significant improvements in social functioning, possibly due to the lack of immersive realism (Minor et al. 2022). There were also statistically non‐significant improvements found for social cognition, disorganised symptoms and negative beliefs about the uncontrollability of thoughts (Han and Lee 2022; Minor et al. 2022). Future studies with larger samples sizes would enhance the statistical power to examine between group effects and to identify the effectiveness of the above interventions (Minor et al. 2022; Han and Lee 2022). Additionally, given that only schizophrenia samples were studied, it remains unclear whether and to what extent these metacognitive interventions may benefit early psychosis samples such as CHR and FEP.

### 3.6 Occupational Therapy (OT)

Three studies examined OT in improving social functioning and daily living skills among individuals with schizophrenia who had a psychiatric admission in the past year (Shimada et al. 2016, 2018, 2022). These OT interventions aimed to enhance self‐awareness, motivation and cognitive skills through personalised and group‐based activities, with a focus on improving communication and social interaction. All three studies focused on activities targeting daily living skills, such as time management, household tasks and social interactions, which have been deemed critical for functional recovery (Shimada et al. 2016, 2018, 2022). Additionally, these interventions included psychoeducation components, such as relapse prevention, crisis planning and discharge preparation, in order to support clients with their transition to community living (Shimada et al. 2016, 2018, 2022). These studies differed in their intervention formats, with two employing individualised occupational therapy (IOT) (Shimada et al. 2016, 2018) and one utilising group occupational therapy (GOT) (Shimada et al. 2022). Both IOT and GOT were delivered three to five times per week, with sessions lasting 1–2 h but differed in their primary focus: IOT prioritised individualised skill‐building and was delivered in a one‐to‐one format, while GOT emphasised group engagement and social participation through cooking and music (Shimada et al. 2016, 2018). Long‐term follow‐up (5 years) revealed that IOT was associated with sustained benefits in employment readiness and independent living, whereas GOT was more effective in promoting group engagement and social participation (Shimada et al. 2022). These differences suggest that the choice between IOT and GOT should be guided by the individual's specific needs, with IOT being more suitable for skill‐building and long‐term recovery and GOT for enhancing socialisation and group engagement.

In terms of clinical outcomes, all three studies demonstrated statistically significant improvements following the intervention in terms of reduced positive and negative symptoms of psychosis, improved cognition, increased motivation and higher client satisfaction (Shimada et al. 2016, 2018, 2022). There were also statistically non‐significant improvements found for motor speed, medication adherence and employment (Shimada et al. 2016, 2018, 2022). However, findings regarding social functioning were mixed, with statistically significant improvements observed when the Social Functioning Scale (SFS) was used regardless of whether an individualised or group format was employed (Shimada et al. 2022). In contrast, statistically non‐significant findings for social functioning were found when studies employed the Global Assessment of Functioning (GAF) scale (Shimada et al. 2016, 2018). These mixed findings regarding social functioning outcomes might be attributed to the differing measures employed. For example, the GAF is a subjective measure of functioning on a scale of 0–100 as rated by the observer, while the SFS assesses social functioning across a number of domains including social engagement/withdrawal, interpersonal, prosocial, recreation, independence–competence/performance and employment as rated by the individual themselves, thereby rendering this scale a more comprehensive measure of social functioning (Birchwood et al. 1990).

### 3.7 Social Skills Training (SST)

Nine studies examined SST interventions, eight in schizophrenia samples (Baskaran et al. 2023; Fulford et al. 2021; Inchausti et al. 2018; Rus‐Calafell et al. 2013; Karaman et al. 2020; Torres et al. 2002; Yildiz et al. 2004, 2018) and one in a CHR sample (Addington et al. 2023). All studies found statistically significant improvements in social functioning in SZ/CHR compared to TAU. Statistically significant improvements following the intervention also included reduced positive and negative symptoms of psychosis, defeatist beliefs, depression and anxiety, as well as improved verbal and non‐verbal skills, cognition, metacognition, social interaction and QoL (Addington et al. 2023; Baskaran et al. 2023; Inchausti et al. 2018; Karaman et al. 2020; Yildiz et al. 2004, 2018). Statistically non‐significant improvements were also found for stigma, motivation, metacognition, employment and social withdrawal (Addington et al. 2023; Fulford et al. 2021; Inchausti et al. 2018; Torres et al. 2002).

The interventions that achieved statistically significant improvements in social functioning shared several key features, namely, being structured; using interactive methods such as role‐playing and goal setting; being guided by trained therapists/mobile apps; integrating cognitive‐behavioural problem‐solving and coping strategies; and utilising group settings to improve social interaction, motivation and peer support (Addington et al. 2023; Baskaran et al. 2023; Fulford et al. 2021; Inchausti et al. 2018; Rus‐Calafell et al. 2013; Karaman et al. 2020; Torres et al. 2002; Yildiz et al. 2004, 2018). This therefore highlights that effective interventions combined practical skill‐building with professional guidance and a supportive group environment. Most studies delivered the intervention in group settings, which facilitated peer interaction, and followed structured manualised protocols to ensure consistency (Addington et al. 2023; Inchausti et al. 2018; Yildiz et al. 2018). Furthermore, many interventions emphasised the application of skills in real‐life contexts, such as practising with family or friends to improve functional recovery (Baskaran et al. 2023; Fulford et al. 2021; Rus‐Calafell et al. 2013). Despite these commonalities, studies varied in the delivery methods and formats employed. For example, some interventions utilised mobile apps (Fulford et al. 2021), while others relied on in‐person sessions or used board games to engage participants (Addington et al. 2023; Inchausti et al. 2018; Torres et al. 2002). The target population also varied, ranging from CHR to chronic schizophrenia, highlighting the adaptability of SST to address diverse clinical populations (Addington et al. 2023; Baskaran et al. 2023; Yildiz et al. 2018). However, only one study to date has examined SST in CHR and none in FEP, highlighting the need for future research to examine the effectiveness of SST in early psychosis.

### 3.8 Virtual Reality (VR) Interventions

Six studies examined VR interventions, five in schizophrenia samples (Adery et al. 2018; Hoşgelen et al. 2023; Nijman et al. 2023; Rus‐Calafell et al. 2014; Shen et al. 2022) and one in a CHR sample (Alvarez‐Jimenez et al. 2018). Five studies found statistically significant improvements in social functioning in SZ/CHR compared to TAU (Alvarez‐Jimenez et al. 2018; Hoşgelen et al. 2023; Nijman et al. 2023; Rus‐Calafell et al. 2014; Shen et al. 2022). Statistically significant improvements following the intervention also included reduced positive and negative symptoms of psychosis, stress, anxiety, avoidance and distress, along with enhanced mindfulness, life satisfaction, facial emotion recognition, social interaction and metacognition (Alvarez‐Jimenez et al. 2018; Adery et al. 2018; Hoşgelen et al. 2023; Nijman et al. 2023; Rus‐Calafell et al. 2014; Shen et al. 2022). Statistically non‐significant improvements were also found for depression, social cognition and theory of mind (ToM) (Alvarez‐Jimenez et al. 2018; Nijman et al. 2023; Shen et al. 2022). Intervention duration varied across studies, ranging from 10 sessions over 5 weeks to 16 sessions over 8 weeks (Hoşgelen et al. 2023; Nijman et al. 2023). All interventions that demonstrated statistically significant social functioning outcomes included immersive, realistic scenarios focused on social and cognitive skills. These interventions allowed participants to practice essential skills in a controlled environment with tailored experiences and active therapist involvement, which aided confidence building for real‐life application (Alvarez‐Jimenez et al. 2018; Hoşgelen et al. 2023; Nijman et al. 2023; Rus‐Calafell et al. 2014; Shen et al. 2022). In contrast, the study that demonstrated non‐significant findings employed less immersive, menu‐based interactions with pre‐recorded sounds and feedback, suggesting that the effectiveness of VR interventions may depend on the degree of immersion and interactivity (Adery et al. 2018). Overall, the immersive and interactive component of VR appears to be a critical factor in its effectiveness, enabling participants to practice and refine skills in a safe and controlled environment, which appears to be applicable to CHR and schizophrenia. However, only one study to date has explored VR interventions to promote social functioning in CHR, which therefore necessitates further investigation, and no VR interventions have been applied to FEP, which highlights avenues for future research.

### 3.9 Online Interventions

Five studies examined online interventions, two in FEP samples (Alvarez‐Jimenez et al. 2021; Lal et al. 2023) and three in schizophrenia samples (Nahum et al. 2014, 2021; Özer and Dişsiz 2024). The three schizophrenia studies found statistically significant improvements in social functioning in patients compared to TAU (Nahum et al. 2014, 2021; Özer and Dişsiz 2024), while the FEP studies were non‐significant. Statistically significant improvements following the intervention included reduced positive and negative symptoms, enhanced QoL and increased temporal pleasure experience (Nahum et al. 2014, 2021; Özer and Dişsiz 2024). Statistically non‐significant improvements were also found for depression, self‐esteem, satisfaction with life, QoL, facial memory, managing emotions, motivation and employment status (Alvarez‐Jimenez et al. 2021; Lal et al. 2023; Nahum et al. 2014, 2021; Özer and Dişsiz 2024). Interventions that demonstrated statistically significant social functioning outcomes targeted cognitive and emotional processes to enhance social skills. Specifically, the SocialVille intervention employed computerised exercises to enhance social cognition and improve the processing and interpretation of social information (Nahum et al. 2014). This intervention also employed computerised exercises to enhance visual and vocal perception, social cue recognition and empathy by using socially relevant stimuli to improve processing speed and accuracy in social information (Nahum et al. 2021). The acceptance and commitment therapy (ACT) intervention promoted flexibility and emotion management through an online group therapy centred on ACT principles (Özer and Dişsiz 2024). In contrast, the non‐significant FEP studies only focused on moderated online networking, peer interaction and community support (Alvarez‐Jimenez et al. 2021; Lal et al. 2023). This suggests that interventions that combine targeted cognitive and emotional training with structured skill development may be more effective in improving social functioning compared to those relying solely on peer support and networking.

### 3.10 Psychosocial Interventions

Fourteen studies examined psychosocial interventions, 13 in schizophrenia samples (Atkinson et al. 1996; Dabit et al. 2021; Fowler et al. 2009; Granholm et al. 2005; Girón et al. 2010; Li et al. 2018; Restek‐Petrović et al. 2014; Ruiz‐Iriondo et al. 2020; Štrkalj‐Ivezić et al. 2013; Varga et al. 2018; Veltro et al. 2011; Wong et al. 2024; Zimmer et al. 2007) and one in an FEP sample (Herman et al. 2018). All studies found statistically significant improvements in social functioning in SZ/FEP compared to TAU. Statistically significant improvements following the interventions also included reduced positive and negative symptoms, fewer relapses and hospital admissions, reduced family burden, increased independent living skills, higher life satisfaction and quality, improved cognition and insight, improved romantic relationships, reduced social anxiety, improved verbal learning, higher self‐esteem and decreased dysfunctional attitudes. There were also statistically non‐significant improvements found for suicidal thoughts, hopelessness, depression, employment status, stigma, medication compliance, communication and defeatist beliefs (Atkinson et al. 1996; Dabit et al. 2021; Fowler et al. 2009; Granholm et al. 2005; Girón et al. 2010; Li et al. 2018; Restek‐Petrović et al. 2014; Veltro et al. 2011; Wong et al. 2024).

Interventions that demonstrated statistically significant social functioning outcomes employed tailored and multifaceted approaches. Cognitive‐behavioural SST interventions integrated cognitive and social skills with problem‐solving to address cognitive impairments and enhance interpersonal functioning (Granholm et al. 2005; Herman et al. 2018). Similarly, CBT with vocational case management interventions focused on personal goals and structured social activities to promote functional recovery (Fowler et al. 2009). Problem‐solving interventions improved coping skills and social interactions through cognitive restructuring (Veltro et al. 2011), while strength‐based CBT leveraged personal strengths to overcome barriers and foster resilience (Wong et al. 2024). These findings highlight the importance of combining cognitive, behavioural and SST within a structured framework to address the complex needs of individuals with psychosis.

The majority of studies employed group‐based interventions that incorporated elements of CBT, SST and psychoeducation. One study employed structured group sessions to address social behaviour in individuals with schizophrenia (Atkinson et al. 1996), whilst another study used a similar group‐based approach to target cognitive deficits and improve social functioning in older aged clients with schizophrenia (Granholm et al. 2005). Other studies integrated cognitive and SST within group settings, demonstrating the effectiveness of combining these methods to enhance social cognition and interpersonal problem‐solving (Dabit et al. 2021; Zimmer et al. 2007). Individualised goal setting and problem‐solving techniques were also emphasised in several studies, as these strategies fostered motivation and promoted social recovery (Fowler et al. 2009; Herman et al. 2018). Additionally, family involvement also emerged as a critical component in one study, where family‐based psychoeducation and problem‐solving strategies significantly reduced relapse rates and improved social functioning (Girón et al. 2010). These findings collectively highlight the versatility and efficacy of group‐based psychosocial interventions in addressing the multidimensional challenges faced by individuals with psychosis. Despite these similarities, the studies varied in their focus on specific deficits versus a more holistic approach to treatment. Some interventions targeted specific areas, such as emotional management through structured cognitive‐behavioural and emotional training or stigma reduction and self‐esteem through psychoeducation and SST (Ruiz‐Iriondo et al. 2020; Li et al. 2018). In contrast, other studies adopted comprehensive rehabilitation programmes that integrated multiple psychosocial strategies, including social and life skills training, relapse prevention and OT to address broader functional outcomes (Štrkalj‐Ivezić et al. 2013; Varga et al. 2018). The integration of cognitive, behavioural and SST within group‐based formats, combined with individualised goal setting and family involvement, appears to be effective in improving social functioning in psychosis. However, as most studies examined chronic schizophrenia samples, future research would benefit from assessing which strategies and interventions are deemed effective for early psychosis samples such as CHR and FEP. Additionally, as most psychosocial interventions employed a group‐based format, it highlights the need to enhance individual CBTP practices to enhance social functioning.

### 3.11 Application of Findings to Clinical Contexts

The findings from this review highlight the importance of tailoring interventions to the specific needs of individuals at different stages of psychosis and across clinical settings. For example, individuals at CHR were found to benefit from SST and VR interventions. However, as these interventions were only examined by one study each, more research is needed to corroborate their effectiveness.

For FEP clients, SRT, psychosocial interventions and physical exercise groups were found to be effective in improving social functioning and reducing positive and negative symptoms of psychosis. Physical exercise interventions were particularly beneficial for young psychosis clients, especially when sessions were 60–120 min in duration and were tailored to the individual's physical capabilities. As these interventions were found to be effective for FEP samples, it highlights the importance of integrating them into early intervention services to promote long‐term recovery.

For non‐affective psychosis, SRT demonstrated significant improvements in social functioning and vocational outcomes; however, its benefits for affective psychosis remain less clear.

For individuals with schizophrenia, SST and OT interventions were found to be effective especially when delivered in a structured, group‐based format that focused on skill‐building, social interaction and the promotion of daily living skills. VR and online interventions also demonstrated promise for SZ samples, especially when immersive and interactive scenarios were used to practice social and cognitive skills in a controlled environment.

In regard to inpatient settings, OT interventions that focused on promoting daily living skills, relapse prevention and discharge preparation were associated with improved transition to community living and functional outcomes in SZ. Individualised OT was associated with sustained benefits in employment readiness and independent living, whereas GOT was more effective in promoting group engagement and social participation, highlighting the need to adapt the delivery format based on the clients' therapeutic goals. Additionally, outpatients with SZ were found to benefit from SST and psychosocial interventions that combined cognitive‐behavioural techniques with SST, as these approaches improved social functioning and reduced positive and negative symptoms of psychosis.

Art interventions and metacognitive interventions also enhanced social functioning and reduced positive and negative symptoms of psychosis in SZ, but further research is needed to determine their applicability to FEP and CHR populations.

By tailoring interventions to the specific needs of individuals at different stages of psychosis and within different clinical settings, clinicians can optimise outcomes and support long‐term recovery. Future research would benefit from standardising interventions and outcome measures, exploring the effectiveness of interventions across different stages of psychosis and investigating the long‐term benefits of these interventions via the use of long‐term follow‐ups.

### 3.12 Low Social Functioning Factors

Given the substantial heterogeneity in social functioning interventions, the identification of factors found to be associated with poor social functioning in psychosis from the evidence base could help inform intervention development. Indeed, the factors that were associated with poor social functioning (see Table 5) included deficits in social cognition (Addington et al. 2006; García‐López et al. 2022; Woolverton et al. 2018), ToM (Sullivan et al. 2013, 2014) and neurocognition (Chang et al. 2017), as well as symptom‐related factors such as social anxiety (Chudleigh et al. 2011; González‐Blanch et al. 2022; Romm et al. 2012), depression (Chudleigh et al. 2011), negative symptoms (Clay et al. 2021; Corcoran et al. 2011; González‐Blanch et al. 2020; Schlosser et al. 2015; Voges and Addington 2005) and positive symptoms (Phalen et al. 2017). Self‐perception factors included self‐esteem (Lysaker et al. 2007), self‐efficacy (Chang et al. 2017) and internalised stigma (Hill and Startup 2013; Lysaker et al. 2007). Behavioural factors included poor motivation (Chang et al. 2017; Moe et al. 2021), social relatedness (González‐Blanch et al. 2020) and avoidance of goal‐directed activity (Schlosser et al. 2015). Cognitive factors included appraisals (Granholm et al. 2013; Henry et al. 2008), negative self‐beliefs (González‐Blanch et al. 2020; Patton et al. 2022), dependency and enmeshment schema (Taylor and Harper 2017), negative self‐statements (Voges and Addington 2005), defeatist performance beliefs (Clay et al. 2021; Grant and Beck 2009) and metacognitive beliefs concerning uncontrollability and danger (Bright et al. 2018). Furthermore, emotion‐related factors included reduced emotional awareness (Kimhy et al. 2012; Kimhy et al. 2016), and emotional regulation (Vines et al. 2022), negative affect (Grove et al. 2016) and emotional suppression (Perry et al. 2011).

**TABLE 5:** Factors associated with high or low social functioning in psychosis.

Author	Factor	Country and type of study	Sample size and setting	Mean age (SD)	Questionnaire and diagnostic tools	Main findings and clinical implications
Addington et al. (2006)	Social cognition	Canada Longitudinal	50 FEP 53 SZ 55 HCs Outpatient	FEP 25.1 (8.0) SZ 35.5 (7.2) HCs 21.7 (6.1)	DSM‐4 Social Cue Recognition Test (SCRT) Situational Features Recognition Test (SFRT) Controlled Oral Word Association Test (COWAT) Wisconsin Card Sorting Test (WCST) Degraded stimulus continuous performance test (DS–CPT) Span of apprehension (SPAN) Quality of Life Scale (QLS) Assessment of Interpersonal Problem Solving (AIPPS) Receiving–processing– sending (RPS) Positive and Negative Syndrome Scale for Schizophrenia (PANSS)	There were significant associations between social cognition, cognition and social functioning in all three groups (FEP/SC/HCs). Deficits in social cognition were stable over time. In the first two groups (FEP/SZ), controlling for social cognition reduced the relationship between cognitive and social functioning. The study provides some evidence that social cognition mediates the relationship between cognitive and social functioning. There are clinical implications, in that the recognition of the importance of social cognition has led to the development of interventions to specifically address social cognition.
Bright et al. (2018)	Metacognitive beliefs	UK Longitudinal	109 ARMS (63%M/46F) Outpatient	20.71 (4.34)	Comprehensive Assessment of At Risk Mental States (CAARMS) Global Assessment of Functioning scale (GAF) Social and Occupational Functioning Assessment Scale (SOFAS) Time Use Survey (TUS) Meta‐Cognitions Questionnaire‐30 (MCQ‐30) Beliefs about the Self and Others (BCSS) Beck Depression Inventory‐7 (BDI7) Social Interaction Anxiety Scale (SIAS)	Metacognitive beliefs concerning uncontrollability and danger of worry were found to negatively predict structured activity. This was after controlling for age, gender, treatment allocation, cognitive schemas, positive symptom severity, social anxiety and depression. Metacognitive danger items were most important. Age was the only control variable found to be an independent predictor of structured activity in the regression model, despite negative bi‐variate relationships with structured activity found across three cognitive schema subscales and social anxiety. This is the first study to find that higher negative metacognitive beliefs about uncontrollability and danger predict lower social functioning in an ARMS sample and that the perception of thoughts being dangerous was of particular importance. Psychological interventions should consider targeting this metacognitive dimension to increase social functioning. Future longitudinal research is required to strengthen findings in this area. Higher negative metacognitive beliefs about uncontrollability and danger predict lower social functioning in an ARMS sample. Small negative relationships existed between both negative cognitive schema subscales and structured activity. A small positive correlation was present between the positive beliefs about self‐cognitive schema subscale and structured activity. Psychological interventions should consider targeting this metacognitive dimension to increase social functioning. Future longitudinal research is required to strengthen findings in this area.
Chang et al. (2017)	Motivation	Hong Kong Cross‐sectional	321 FEP (142M/179F) Outpatient	38.31 (8.42)	DSM‐4 Interview for Retrospective Assessment of Onset of Schizophrenia (IRAOS) Positive and Negative Syndrome Scale (PANSS) Scale for the Assessment of Negative Symptoms (SANS) Chinse version of the General Self‐Efficacy Scale (CGSS) Social and Occupational Functioning Assessment Scale (SOFAS) Wechsler Adult Intelligence Scale – Revised (WAIS‐R) Wechsler Memory Scale – Revised (WMS‐R) Modified Wisconsin Card Sorting test (MWCST)	Low motivation, neurocognitive impairment and general self‐efficacy exerted direct effect on social functioning. An indirect effect of neurocognition on functioning was mediated by amotivation. Amotivation also mediated the relationship between general self‐efficacy and functioning. Further investigation is warranted to clarify the neurobiological basis underlying amotivation in psychotic disorders, for instance dysfunctional reward processing (Strauss et al. 2014; Chang et al. 2016c) and altered effort‐cost computation (Gold et al. 2015), to facilitate development of effective treatments to ameliorate motivational impairment and therefore its adverse impacts on functional outcome.
Chudleigh et al. (2011)	Social anxiety and negative symptoms	Australia Cross‐sectional	20 FEP (65%M/35F%) 20 ARMS (55%M/45%F) 20 HCs (50%M/50%F) Outpatient	ARMS 20.75 (2.7) FEP 22.05 (3.0) HCs 22.00 (2.5)	Comprehensive Assessment of At Risk Mental States (CAARMS) Brief Psychiatric Rating Scale (BPRS) Social Functioning Scale (SFS) World Health Organisation Disability Assessment Scale II (WHODAS) Depression Anxiety Stress Scale (DASS) Social and Occupational Functioning Assessment Scale (SOFAS)	At‐risk individuals had comparable social deficits to the FEP group, and both patient (ARMS and FEP) groups had significantly poorer social functioning than controls. Importantly, social functioning was most strongly associated with depressive and social anxiety symptoms and to a lesser extent with positive symptoms. Psychiatric symptoms such as depression and social anxiety may provide an avenue for early interventions of social functioning deficits in psychosis.
Clay et al. (2021)	Defeatist beliefs and negative symptoms	Georgia Cross‐sectional	Study 1 184 SZ (61%M/39%F) 184 HCs (56%M/44%F) Study 2 35 CHR (23%M/77%F) 30 HCs (33%M/67F) Outpatient	Study 1 SZ 39.61 (11.21) HCs 39.59 (10.67) Study 2 CHR 21.80 (3.38) HCs 21.43 (2.18)	Structured Clinical Interview for DSM‐IV‐TR DSM‐5 Defeatist Performance Beliefs (DPB) Questionnaire Brief Negative Symptom Scale (BNSS) Brief Psychiatric Rating Scale (BPRS) Global Functioning Scale Role (GFSR) Global Functioning Scale Social (GFSS) Level of Functioning Scale (LOF) MATRICS Consensus Cognitive Battery (MCCB) Negative Symptom Inventory Prodromal—Revised (NSIPR) Structured Interview for Prodromal Syndromes (SIPS)	Significant associations between higher defeatist performance beliefs and greater severity of negative symptoms, poorer functioning and impaired social cognition. Both chronic SZ and CHR participants had elevated defeatist performance beliefs compared to HCs (*p* < 0.01). In SZ, higher defeatist performance beliefs were associated with greater negative symptoms (*p* < 0.01), poorer social functioning and impaired social cognition (*p* < 0.001). In CHR, greater *p* < 0.01 were associated with poorer social functioning (*p* < 0.05) and impairments in the neurocognitive domains of reasoning (*p* < 0.05) and processing speed (*p* < 0.05). Defeatist performance beliefs may still be an important psychological target for reducing functional decline during the prodromal period, potentially offering a window for halting social and role decline.
Corcoran et al. (2011)	Negative symptoms	USA Cross‐sectional	56 CHR(43M/13F) 22 HCs (13M/9F) Outpatient	CHR 19.6 (3.6) HCs 21.0 (3.5)	Structured Interview for Prodromal Syndromes (SIPS) Scale of Prodromal Symptoms (SOPS) Hamilton Rating Scale for Depression (HAMD) Wechsler Adult Intelligence Scale (Wechsler) Wechsler Intelligence Scale for Children Social Adjustment Scale – Self‐report (SAS‐SR)	Social function was significantly impaired in this CHR cohort, as compared with healthy controls. The study demonstrated a relationship between social dysfunction and depressive symptoms in clinical high‐risk cases; this association was primarily explained by the relationship of each of these to negative symptoms. Social impairment was related to multiple symptom domains—depressed, negative, disorganisation, general—that were themselves highly correlated with one another. These findings have relevance for potential treatment strategies for social dysfunction in schizophrenia and its risk states and predict that antidepressant drugs, cognitive behavioural therapy and/or social skills training may be effective.
Favrod et al. (2019)	Negative symptoms	Switzerland Cross‐sectional	31 SZ (16M/5F) Outpatient	33 (11.34)	DSM‐4 Scale for Assessment of Negative Symptoms (SANS) Personal and Social Performance scale (PSP) Calgary Depression Scale for Schizophrenia (CDSS) Temporal Experience of Pleasure Scale (TEPS) Savouring Beliefs Inventory (SBI)	Participation in positive emotions programme for schizophrenia is accompanied by a reduction of negative symptoms and an improvement of social functioning. This field test adds external validity to positive emotions programme for schizophrenia to improve the reduced capacity to experience syndrome in schizophrenia. Both negative syndromes, reduction of expression and reduction of experience, are improved.
García‐López et al. (2022)	Social cognition	Spain Cross‐sectional	196 FEP (127M/69F) Outpatient	25.08 (5.67)	DSM‐4 WHO Disability Assessment Schedule (WHODAS) Positive and Negative Schizophrenia Syndrome Scale (PANSS) Mayer‐Salovey‐Caruso Emotional Intelligence Test (MSCEIT)	The study found that there was a direct relationship between emotional processing and social functioning, as well as an indirect connection between them, mediated by the experiential (but not the expressiveness) factor of negative symptoms (i.e., avolition, anhedonia and asociality). In clinical practice, this would translate into worse functioning in patients with greater anhedonia, lack of motivation and social isolation. In this context, it is thought that negative symptoms would lead to less exposure to social situations, leading to decreased opportunities for reinforcement and an atrophy of social skills, which ultimately could evoke higher negative symptoms. The importance of the affectation of subdomains of social cognition, as well as the role of negative symptoms, specifically the experiential factor, in the functioning of patients with FEP seems to be relevant. The inclusion of these factors in prevention and treatment programmes would thus allow us to reduce their impact on the social functioning of these patients.
González‐Blanch et al. (2020)	Self‐beliefs and negative symptoms and social relatedness	Australia Cross‐sectional	170 FEP (90M/80F) Outpatient	20.9 (2.9)	DSM‐4 Personal and Social Performance Scale (PSP) 2‐Way Social Support Scale (2‐Way SSS) UCLA Loneliness Scale, version 3 (UCLA‐3) Mental Health Confidence Scale (MHCS) Self‐Esteem Rating Scale‐ Short Form (SERS‐FS) Positive and Negative Syndrome Scale (PANSS)Theory of mind (ToM) Bell Lysaker Emotion Recognition Task (BLERT)	Negative symptoms, social relatedness and self‐beliefs exerted a direct effect on social functioning. Social relatedness partially mediated the impact of social cognition and negative symptoms on social functioning. Self‐beliefs also mediated the relationship between social relatedness and social functioning. The observed associations highlight the potential value of targeting social relatedness and self‐beliefs to improve functional outcomes in FEP. Explanatory models of social functioning in FEP not accounting for social relatedness and self‐beliefs might be overestimating the effect of the illness‐related factors
González‐Blanch et al. (2022)	Social anxiety	Spain Longitudinal	108 FEP (41%M/49%F) Outpatient	20.9 (2.8)	DSM‐4 Social Interaction Anxiety Scale (SIAS) Personal and Social Performance scale (PSP) Assessment of Quality of Life‐ 8D (AQoL‐8D) Calgary Depression Scale for Schizophrenia (CDSS) Alcohol, Smoking and Substance Involvement Screening Test (ASSIST) Positive and Negative Syndrome Scale (PANSS)	The persistent social anxiety groups presented significantly worse social functioning and HR‐QoL levels at the 18‐month follow‐up when compared to the fluctuating and no anxiety groups. Individuals with persistent social anxiety constitute a highly vulnerable group and may require targeted interventions to improve their social functioning and HR‐QoL.
Granholm et al. (2013)	Appraisals	USA Cross‐sectional	145 SZ (61%M/39F) Outpatient	46.5 (11.2)	DSM‐4 Positive and Negative Syndrome Scale (PANSS) Beck Depression Inventory — II (BDI‐II)	Positive social interaction appraisals at a given time of day were concurrently associated with significantly greater positive, as well as less negative, affect. Social functioning, therefore, was linked to positive performance beliefs about social interactions that were associated with greater positive affect. Positive affect (i.e., happiness) at a given time of day predicted the number of social interactions later in the day. The findings suggest a useful treatment target for cognitive behavioural therapy and other psychosocial interventions that can be used to challenge defeatist beliefs and increase positive affect to enhance social functioning in schizophrenia.
Grant and Beck (2009)	Defeatist beliefs	USA Cross‐sectional	55 SZ (65%M/35%F) 22 HCs (68%M/32%F) Outpatient	SZ 36.9 (9.9) HCs 31.8 (7.8)	Scale for the Assessment of Negative Symptoms (SANS) Scale for the Assessment of Positive Symptoms (SAPS) The Quality of Life Scale (QOLS) Beck's Depression Inventory (BDI‐II) Beck Anxiety Inventory (BAI) Dysfunctional Attitude Scale (DAS) Neurocognitive performance (NP)	Defeatist belief endorsements were mediators in the relationship between cognitive impairment and both negative symptoms and functioning. Eliciting and changing the defeatist performance beliefs could lead to increased engagement in constructive activity in individuals with prominent cognitive impairment and negative symptoms. Defeatist beliefs are, furthermore, measurable targets that can be added to traditional rehabilitation efforts, cognitive remediation programmes, social skills training, as well as cognitive behavioural therapy for schizophrenia.
Grove et al. (2016)	Negative affect	USA Cross‐sectional	277 SZ (150M/127F) 145 P‐NOS (78M/67F) 75 BD (39M/56F) 84 HCs (51M/33F) Outpatient	SZ 43.4 (11.2) P‐NOS 42.3 (10.9) BD 42.2 (11.2) HCs 42.0 (12.2)	DSM‐4 The Brief Assessment of Cognition in SZ (BACS) The Mayer‐Salovey‐Caruso Emotional Intelligence Test (MSCEIT) The Scale for the Assessment of Positive Symptoms (SAPS) The Social Adjustment Scale– Self Report (SAS‐SR) The Differential Emotions Scale (DES) The Psychological Stress Index (PSI) The state subscale of the State–Trait Anxiety Inventory (Form X‐1; STAI‐S) The revised Beck Depression Inventory (BDI‐IA)	SZ (*p* < 0.001), SA (*p* < 0.001) and BD (*p* < 0.001) reported statistically significant higher levels of Negative Affect (NA) compared to HCs. All measures of NA (BDI‐IA, PSI, STAI‐S and DES‐N) showed statistically significant positive correlations with SAS‐SR in SZ, P‐NOS and BD participants: SAS‐SR and BDI‐IA: *r* = 0.75, *p* < 0.001 SAS‐SR and PSI: *r* = 0.55, *p* < 0.001 SAS‐SR and STAI‐S: *r* = 0.58, *p* < 0.001 SAS‐SR and DES‐N: *r* = 0.52, *p* < 0.001 For HCs, all measures of NA were statistically significantly positively correlated with SAS‐SR (*r* = 0.39–0.55; all *p* < 0.001), except for DES‐N (*r* = 0.11, *p* > 0.05). These findings showed that NA was elevated in individuals with SZ, P‐NOS and BD compared with HCs. NA was significantly correlated with social functioning in SZ, P‐NOS, BD and HCs. As such, NA should be routinely assessed in clinical settings and targeted with empirically supported treatments, especially psychosocial interventions, in order to improve social functioning in individuals with SZ, P‐NOS and BD.
Henry et al. (2008)	Reappraisal	Australia Cross‐sectional	41 SZ (46%M/54%F) 38 HCs (55%M/45%F) 36 outpatients and 5 inpatients Average duration of illness 14.6 years (10.50) Outpatient	SZ 37.5 (10.67) HCs 36.1 (11.99)	DSM‐4 Wechsler's Abbreviated Scale of Intelligence (WASI) The Hospital Anxiety and Depression Scale (HADS) The Emotion Regulation Questionnaire (ERQ) The Social Functioning Scale (SFS) Scale for the Assessment of Positive Symptoms (SAPS) Scale for the Assessment of Negative (SANS) Hospital Anxiety and Depression Scale (HADS)	Individuals with SZ and HCs showed no significant differences in the use of suppression and reappraisal as regulatory strategies (reappraisal: *p* = 0.415, suppression: *p* = 0.308). No statistically significant differences were found between SZ and HCs for ERQ items related to the use of positive emotions (*p* = 0.955), use of negative emotions (*p* = 0.700) and regulation of nonvalenced emotional states (*p* = 0.911). In SZ, reappraisal showed a statistically significant positive correlation with better social functioning (*r* = 0.38) and lower depression (*r* = −0.37) but was not statistically significantly correlated with anxiety (*r* = 0.04). Suppression was not statistically significantly correlated with social functioning (*r* = −0.13), depression (*r* = 0.12) or anxiety (*r* = 0.17). In HCs, reappraisal was statistically significantly positively associated with better social functioning (*r* = 0.34), while suppression was statistically significantly positively associated with higher depression (*r* = 0.32). Reappraisal and suppression were not statistically significantly correlated with depression (reappraisal: *r* = −0.23; suppression: *r* = 0.12) or anxiety (reappraisal: *r* = −0.26; suppression: *r* = 0.21). For SAPS subscales in SZ, neither suppression nor reappraisal was statistically significantly correlated with hallucinations (suppression: *r* = 0.03; reappraisal: *r* = −0.10), delusions (suppression: *r* = 0.20; reappraisal: *r* = −0.06), bizarre behaviour (suppression: *r* = 0.03; reappraisal: *r* = −0.12) or thought disorder (suppression: *r* = 0.03; reappraisal: *r* = 0.07). For SANS subscales in SZ, neither suppression nor reappraisal was statistically significantly correlated with blunted affect (suppression: *r* = 0.18; reappraisal: *r* = −0.21), alogia (suppression: *r* = 0.14; reappraisal: *r* = −0.11), apathy (suppression: *r* = 0.30; reappraisal: *r* = −0.07), anhedonia (suppression: *r* = 0.08; reappraisal: *r* = −0.24) or attention (suppression: *r* = 0.25; reappraisal: *r* = −0.28). These findings show that individuals with SZ did not differ from HCs with regard to their self‐reported use of suppression and reappraisal as methods of regulating emotion experience and expression. For both SZ group and HCs, greater use of reappraisal was associated with fewer social function difficulties, whereas for the SZ group increased use of reappraisal was also associated with significantly reduced depression. For the SZ group, self‐reported use of suppression was not significantly correlated with symptoms of SZ (blunted affect, alogia, apathy, anhedonia or attention). These authors suggest that it is important to develop cognitive‐behavioural techniques focused on improving emotion regulation in SZ.
Hill and Startup (2013)	Stigma	Australia Cross‐sectional	SZ 60 (44M/16F) Outpatient	34.4 (59.58)	DSM‐4 National Adult Reading Test (NART) Calgary Depression Scale (CDS) Internalised Stigma of Mental Illness Scale (ISMIS) Quality of Life Scale, Abbreviated (QOLSA) Scale for the Assessment of Negative Symptoms (SANS) Self‐Efficacy Questionnaire (SEQ) Faux Pas Recognition Test	Internalised stigma was strongly correlated with negative symptoms, social functioning and self‐efficacy. Furthermore, self‐efficacy was strongly related to negative symptoms and moderately associated with social functioning. The research has highlighted the importance of evidence‐based psychological interventions aimed at targeting internalised stigma in the hope of reducing the impact of debilitating stigmatising beliefs. In addition, such interventions may have positive consequences for self‐efficacy, negative symptoms and social functioning. Furthermore, interventions which focus upon negative symptom management may have the advantage of reducing the distress related to such symptomatology and, in turn, might reduce stigmatising beliefs, as well as ultimately improve one's sense of self‐efficacy. Subsequently, such interventions have the potential to enhance social functioning and adaptation.
Jalbrzikowski et al. (2014)	Coping strategies	USA Longitudinal	88 CHR (58M/30F) 53 HCs (27M/26F) Setting not specified	CHR 17.9 (3.4) HCs 17.8 (4.4)	DSM‐4 Global Functioning Social (GSF) Global Functioning Role (GRF) Wechsler Abbreviated Scale of Intelligence (WASI) Brief Cope Questionnaire Structured Interview of Psychosis‐risk Syndromes (SIPS)	CHR youth showed a statistically significant higher use of maladaptive coping strategies (*p* < 0.001) and a statistically significant lower use of adaptive coping strategies (*p* < 0.01) compared to HCs. More adaptive coping was associated with significantly fewer negative symptoms (*r* = −0.31, *p* = 0.003) and higher levels of social functioning (*r* = −0.32, *p* = 0.003) in CHR youth. No statistically significant relationship between coping styles and social functioning was reported for HCs. There was a statistically significant association between increased use of adaptive coping strategies and better social functioning (*p* = 0.001) and lower levels of negative (*p* < 0.001) and disorganised symptoms over time (*p* = 0.005). Similarly, there was a statistically significant association between increased use of maladaptive coping strategies and positive (*p* = 0.003), negative (*p* = 0.002), disorganised (*p* = 0.025) and general symptoms (*p* = 0.003). Longitudinal analyses of CHR individuals showed that adaptive coping did not significantly change over 12 months, but there were significant decreases in reported maladaptive coping strategies. The findings reveal that youth at CHR for psychosis use maladaptive coping strategies more and adaptive strategies less compared to healthy controls. More adaptive coping is linked to fewer negative symptoms and better social functioning in CHR youth, while maladaptive coping is associated with increased positive, negative, disorganised and general symptoms. Longitudinal analyses revealed that while adaptive coping remained stable over 12 months, maladaptive coping strategies significantly decreased during the same period. These findings suggest that maladaptive coping styles may be more malleable and easier to target than adaptive coping styles. Implementing interventions to teach adaptive coping styles and simultaneously reduce maladaptive coping styles in CHR individuals may also affect psychosocial functioning and clinical symptomatology. Identifying the coping strategies used by CHR individuals may aid in targeting specific interventions that may be useful in preventing the onset of psychosis.
Kimhy et al. (2012)	Emotional awareness and regulation	USA Cross‐sectional	SZ 44 (64%M/36%F) HCs 20 (50%M/50%F) Inpatient and outpatient	SZ 30.33 (8.08) HCs 24.20 (4.62)	DSM‐4 Toronto Alexithymia Scale (TAS‐20) Emotion Management Task (EMT) The Emotion Regulation Questionnaire (ERQ) Provision of Social Relations Scale (PSRS) Self‐Reflection index of the Beck Cognitive Insight Scale (BCIS‐SR) MATRICS Consensus Cognitive Battery (MCCB)	Compared to HCs, individuals with SZ display significantly lower emotion awareness as indexed by having more difficulties identifying and describing their own feelings. Likewise, individuals with SZ display deficits in emotion regulation as characterised by a significantly higher use of suppression and lower use of reappraisal. Among individuals with SZ, difficulties with emotion awareness are strongly predictive of poorer social functioning. As such, it is important to design interventions focusing on targeting deficits in emotional awareness and regulation. As deficits in both emotion awareness and emotion regulation have been linked to poor social functioning, these results invite speculation about the potential to improve social dysfunction by ameliorating such deficits. Increasing patients' abilities to identify and describe emotions is an important component of many contemporary psychotherapy approaches including ACT, Mindfulness‐Based CBT, DBT, as well as Emotion‐Focused Therapy.
Kimhy et al. (2016)	Emotional awareness	USA Cross‐sectional	54 CHR (28M/26F) 87 SZ (50M/27F) HC (2M/48F) Outpatient	CHR 20.18 (3.41) SZ 33.45 (9.47) HC 23.04 (4.10)	DSM‐4 Structured Interview for Psychosis‐Risk Syndromes (SIPS) Toronto Alexithymia Scale (TAS‐20) Emotion Regulation Questionnaire (ERQ) Global Functioning Scale: Social (GFS:S) Wechsler Abbreviated Scale of Intelligence Beck Depression Inventory (BDI) Beck Anxiety Inventory (BAI)	Group comparisons indicated significant differences between HC and the two clinical groups in their ability to identify and describe feelings, as well as the use of suppression and reappraisal emotion‐regulation strategies. Specifically, the CHR and schizophrenia groups displayed comparable deficits in all domains of emotion awareness and emotion regulation. Additionally, a link between poor emotional awareness and poor social functioning in CHR individuals was found, with difficulties in emotional awareness predicting 23.2% of the variance in social functioning. The results indicate that CHR individuals display substantial emotion awareness and emotion‐regulation deficits, at severity comparable with those observed in individuals with schizophrenia. Such deficits, in particular difficulties describing feelings, predate the onset of psychosis and contribute significantly to poor social functioning in this population.
Lysaker et al. (2007)	Stigma	Indiana Cross‐sectional	75 SSD (64M/11F) Outpatient	48.25 (7.01)	*Diagnostic and Statistical Manual of Mental Disorders*, fourth edition (DSM‐IV) (SCID) Positive and Negative Syndrome Scale (PANSS) Quality of Life Scale (QOLS) Internalized Stigma of Mental Illness Scale (ISMIS) Multidimensional Self‐esteem Inventory (MSEI) Beck Hopelessness Scale (BHS)	High insight/minimal stigma group also had significantly less impaired social function than the other groups. If dysfunctional beliefs stemming from social stigma impact social functioning in such an enduring manner, tailored interventions could be devised to help persons com‐ bat these self‐stigmatising beliefs.
Moe et al. (2021)	Motivation	USA Longitudinal	54 FEP (40M/14F) Outpatient	21.67 (3.89)	DSM‐5 Clinical Assessment Interview for Negative Symptoms (CAINS) Social Functioning Scale (SFS) World Health Organization Quality of Life Scale‐Brief (WHOQOL) Positive and Negative Syndrome Scale (PANSS)	Baseline peer social motivation was associated with both social functioning and social quality of life, while expectation of socialisation in the coming week was associated with only social quality of life. Baseline peer social motivation was the only significant predictor of 6‐month change in social functioning. The results suggest that the unique contribution of aspects of social motivation has implications for treatment, including the importance of developmentally informed interventions to improve peer socialisation in youth and young adults with psychosis.
Patton et al. (2022)	Core beliefs	USA Longitudinal	73 CHR (37M/46F) Outpatient	18.6 (1.8)	DSM‐4 Brief Core Schema Scales (BCSS) Structured Interview for Psychosis‐Risk Syndromes (SIPS) Global Functioning Scale (GFS) Beck Depression Inventory—II (BDI‐II)	Longitudinal changes in self‐beliefs within a CHR sample were associated with symptoms and functioning at 12‐month follow‐up. For depression, changes in self‐beliefs from baseline to follow‐up predicted more negative symptoms and worsening social and role functioning at follow‐up. An increase in negative self‐beliefs marginally or significantly predicted depression and social functioning outcomes. The current study shows incremental validity of self‐beliefs from a novel perspective. It suggests that worsening self‐beliefs have a clinically meaningful impact within CHR populations.
Pennou et al. (2021)	Theory of mind	Canada Cross‐sectional	37 FEP (28M/9F) Outpatient	25.1 (4.1)	Structured Clinical Interview for Diagnosis (SCID, DSM‐IV) Brief Psychiatric Rating Scale‐Expanded (BPRS‐E) First Episode Social Functioning Scale (FESFS) Digit Span (Forward and Backward) of the Wechsler Adult Intelligence Scale Trail‐Making Test‐A/B Combined Stories Task	A first look at the results shows us that theory of mind seems to be more predictive depending on the domain of social functioning assessed. The study shows the potential contributions of ToM related processes, especially the reasoning about someone else's beliefs, on certain domains of social functioning. More studies are necessary to replicate these results in larger samples of FEP patients, while adopting longitudinal designs in order to firmly establish the predictive value of ToM deficits in this population.
Perry et al. (2011)	Emotional suppression	Sydney Cross‐sectional	33 SZ (gender unclear) 36 HCs (gender unclear) Outpatient	SZ 43.7(9.89) HCs 40.8 (11.49)	Wechsler Abbreviated Scale of Intelligence (WASI) Scale for the Assessment of Negative Symptoms (SANS) Scale for the Assessment of Positive Symptoms (SAPS) Social Functioning Scale Depression Anxiety Stress Scales (DASS) Emotion Regulation Questionnaire (ERQ) Acceptance and action questionnaire (AAQ)	No statistically significant group differences were found between individuals with SZ and HCs in the use of suppression or reappraisal strategies (all *p* > 0.05), but individuals with SZ reported less use of acceptance (*p* < 0.001). For the SZ group, greater use of reappraisal showed a statistically significant negative correlation with negative symptoms (*r* = −0.48, *p* = 0.005). Increased use reappraisal was related to decreased levels of depression (*r* = −0.40, *p* = 0.021), and greater use of suppression was associated with poorer social functioning (*r* = −0.40, *p* = 0.022). For the SZ group, greater use of acceptance showed a statistically significant negative correlation with depression (*r* = −0.36, *p* < 0.05), anxiety (*r* = −0.41, *p* < 0.05) and stress (*r* = −0.40, *p* < 0.05) and a statistically significant positive correlation with social functioning (*r* = 0.50, *p* = 0.003). For HCs, greater use of acceptance showed a statistically significant negative correlation with depression (*r* = −0.56, *p* < 0.05), anxiety (*r* = −0.39, *p* < 0.05) and stress (*r* = −0.39, *p* < 0.05) Individuals with SZ do not differ in their use of suppression or reappraisal compared with HCs. However, they report significantly less use of acceptance strategies. For the SZ group, greater use of reappraisal is significantly associated with fewer negative symptoms and lower levels of depression, indicating its potential benefit in symptom management. Although greater use of suppression is linked to poorer social functioning. Increased acceptance in the SZ group is significantly associated with reduced depression, anxiety and stress and improved social functioning, suggesting that acceptance‐based interventions may enhance overall well‐being. These findings underscore the importance of promoting acceptance and reappraisal strategies in therapeutic interventions for individuals with SZ to improve their emotional and social outcomes.
Phalen et al. (2017)	Positive symptoms	USA Cross‐sectional	88 SZ (76M/12F) Outpatient	49.58 (8.4)	Structured Clinical Interview for DSM‐IV (SCID) Positive and negative syndrome scale (PANSS) Social Functioning Scale (SFS) The Hinting Test Reading the mind in the eyes test	Mental state reasoning was found to moderate the relationship between persecutory delusions and social functioning, controlling for overall psychopathology. Poor mental state reasoning abilities, more severe delusions of persecution were associated with significant deficits in social functioning. However, for participants with relatively strong mental state reasoning abilities, persecutory delusions did not negatively impact social functioning. Mental state reasoning abilities may buffer the impact of persecutory delusions on social functioning, possibly by helping individuals avoid applying global beliefs of persecution to specific individuals or by allowing for the correction of paranoid inferences. Patients who can develop more comprehensive and integrated understandings of other people may be able to maintain better social relationships even in the presence of persecutory delusions. One promising set of treatments that target this process may be metacognitively oriented psychotherapies such as Metacognitive Reflection and Insight Therapy (MERIT)
Romm et al. (2012)	Social anxiety	Norway Cross‐sectional	144 FEP (92M/52F) Outpatient	12.4 (2.6)	Structured Clinical Interview for DSM‐IV (SCID‐I) Liebowitz Social Anxiety Scale (LSAS‐SR) Insight Scale Rosenberg Self‐Esteem Scale Global Assessment of Functioning (GAF)	The most severe social anxiety group revealed poorer premorbid adjustment, lower social functioning and higher levels of depression. The present study supports previous research that has pointed to the importance of incorporating targeted monitoring and treatment of affective and anxiety symptoms in severe mental illness.
Schlosser et al. (2015)	Negative symptoms	USA Cross‐sectional	85 CHR (58%M/42%) Outpatient	18.67 (4.5)	Structured Interview for Prodromal States (SIPS) Scale of Prodromal Symptoms (SOPS) Scale for the Assessment of Positive Symptoms (SAPS) Scale for the Assessment of Negative Symptoms (SANS) Brief Psychiatric Rating Scale (BPRS) Global Functioning: Social Scale (GF‐S) Measurement and Treatment Research to Improve Cognition in Schizophrenia (MATRICS) Problem solving (D‐KEFS Tower Test) Speed of processing (Trail Making Test Part A; category fluency animal naming) Working memory (letter–number span; WMS‐III spatial span) Verbal learning and verbal memory (HVLT‐R immediate and delayed recall) Visual learning and visual memory (BVMT‐R immediate and delayed recall) problem solving (D‐KEFS Tower Test)	Negative symptom severity was found to be uniquely predictive of social functioning, above and beyond depression/anxiety and neurocognition. Experiential negative symptoms were more predictive of social functioning, relative to expressive symptoms, suggesting that avolition and anhedonia are more important in determining level of social functioning than affective flattening and alogia. Further, the mediated path analysis demonstrated that experiential negative symptoms mediated the relationship between expressive negative symptoms and social functioning. Thus, expressive symptoms may influence social functioning via their effect on experiential symptoms. The results suggest that experiences of motivational impairment are more important in determining social functioning, relative to affective flattening and alogia, in CHR individuals, thereby informing the development of more precise therapeutic targets. Developing novel interventions that stimulate goal‐directed behaviour and reinforce rewarding experiences in social contexts are recommended.
Sullivan et al. (2013)	Theory of mind	UK Cross‐sectional	53 FEP (57%M/43F%) Outpatient	26.7 (8.4)	Social and Occupational Functioning Scale (SOFAS) The Hinting Test (ToM) Positive and Negative Syndrome Scale (PANSS)	There was evidence of an association between social functioning and theory of mind performance when measured using the Hinting test after adjustment for data collection centre, psychotic and negative symptoms and confounders (gender, verbal IQ, memory, and depression scores), indicating that for each unit increase in Hinting test scores there was 70% greater odds of being in the good social functioning group. Social functioning is associated with theory of mind, assessed using the Hinting test, in a sample of people with first episode psychosis. Further, longitudinal research is needed to improve understanding of the causes leading to the poor social functioning suffered by so many with psychotic disorders.
Sullivan et al. (2014)	Theory of mind	UK Longitudinal	61 FEP (57%M/43%F) Outpatient	26.5 (8.3)	Social and Occupational Functioning Scale (SOFAS) The Hinting Test (ToM) Logical Memory Test National Adult Reading Test (NART) Positive and Negative Syndrome Scale (PANSS) Calgary Depression Scale (CDS)	There was no evidence that baseline ToM ability or baseline psychotic symptoms were longitudinally associated with social functioning outcome, suggesting that neither ToM ability nor psychotic symptoms are causally related to social functioning in this population of people with FEP. Further longitudinal work is needed to understand the origin of social functioning deficits in psychosis.
Taylor and Harper (2017)	Schema	UK Cross‐sectional	20 SZ (13M/7F) Outpatient	44 (11.4)	ICD‐10 Young's Schema Questionnaire—Short Version (YSQ‐S) Social Functioning Scale (SFS) Clinical Outcomes In Routine Evaluation (CORE)	Dependency and enmeshment schema were significantly inversely associated with social functioning with a moderate effect size for both (*r*s = −0.50; −0.54, respectively). Eight schemas were significantly associated with distress as measured by the CORE, with effect sizes ranging from moderate to very strong (*r*s = 0.51–0.82). EMSs: Abandonment, Mistrust/Abuse, Social Alienation, Failure, Dependency, Vulnerability to Harm, Enmeshment and Subjugation of needs. These results suggest that early maladaptive schema may have an important role in psychosis and could be considered as part of psychological therapies that seek to enhance social functioning and reduce distress.
Vines et al. (2022)	Emotional regulation difficulties	USA Cross‐sectional	32 CHR (17M/15F) 42 HC (21M/21F) 13 SZ (5M/8F) Setting not specified	CHR 18.5 (2.9) HC 17.8 (2.8) SZ 17.2 (1.7)	Global Functioning: Social Scale (GFS) Global Functioning: Role Scale (GFR) The Emotion Regulation Questionnaire (ERQ) Difficulty in Emotion Regulation Scale (DERS) Wechsler Abbreviated Scale of Intelligence (WASI) Emotion Reactivity Scale (ERS)	CHR (*p* < 0.001) and SZ (*t* = 4.4, *p* < 0.001) participants reported statistically significant elevation in emotion reactivity compared to HCs. CHR reported significantly less engagement in cognitive reappraisal compared to HCs (*t* = 4.3, *p* < 0.001); however, there was no statistically significant difference between SZ and HCs (*t* = 0.96, *p* = 0.34). SZ reported significantly more engagement in cognitive reappraisal compared to CHR (*t* = 2.0, *p* = 0.04). Compared to HCs, SZ were significantly more likely to engage in emotionally expressive behaviour (*t* = 2.9, *p* < 0.001). However, there was no significant difference between CHR and HCs (*t* = 1.7, *p* = 0.08) in expressive suppression. CHR, SZ and HCs demonstrated a statistically significant effect of ERS Total Score on emotion regulation, with higher emotion reactivity being associated with greater impairment on the DERS (*R* ^2^ = 0.16) and less reported engagement in cognitive reappraisal (*R* ^2^ = 0.05). Compared to HCs, CHR (*t* = 6.4, *p* < 0.001) and SZ (*t* = 8.5, *p* < 0.001) endorsed greater overall emotion regulation impairments. In CHR and SZ, greater negative symptom severity was statistically significantly associated with less cognitive reappraisal (*β* = 0.27, *t* = 2.4, *p* = 0.02) and greater overall impairment on the DERS (*β* = 0.31, *t* = 2.4, *p* = 0.02). In CHR and SZ, lower social functioning was statistically significantly associated with greater impairment on the DERS (*β* = 0.30, *t* = 2.1, *p* = 0.04). There was no statistically significant difference between CHR and HCs (*t* = 1.7, *p* = 0.08) in expressive suppression. CHR and SZ endorsed experiencing heightened levels of emotion reactivity and greater difficulty utilising emotion regulation strategies compared to HCs. These impairments were stable across time and adolescent development. Greater levels of emotion reactivity were associated with greater emotion regulation impairments. Greater impairments in emotion regulation were associated with lower social functioning and greater negative symptom severity. Therapeutic interventions designed to reduce emotion reactivity and improve one's ability to utilise emotion regulation strategies may be effective in reducing clinical symptomatology and improving real‐world functioning in CHR and SZ.
Voges and Addington (2005)	Negative self‐statements and negative symptoms	Canada Cross‐sectional	60 FEP (41M/19F) Outpatient	27.45 (8.28)	DSM‐4 Structured Clinical Interview for DSM‐IV (SCID‐I) Social Phobia and Anxiety Inventory (SPAI) Social Interaction Self‐Statement Test (SISST) Quality of Life Scale (QLS) Social Functioning Scale (SFS) Positive and Negative Syndrome Scale (PANSS) Calgary Depression Scale for Schizophrenia (CDSS)	Negative symptoms and negative self‐statements were significant predictors of social functioning. The self‐statements generate negative affect and feelings of anxiety that interferes with their ability to focus on the content of the social interaction, which in turn impedes their performance. The results have implications for addressing these negative cognitions in early psychosis in order to improve social functioning.
Woolverton et al. (2018)	Social cognition	USA Cross‐sectional	71 FEP (76%M/24F) Outpatient	23.09 (4.22)	DSM‐4 Social Functioning Scale (SFS) Internal, Personal and Situational Attributions Questionnaire (IPSAQ) Facial Emotion Identification Test (FEIT) Situational Feature Recognition Test (SFRT) Social Cue Recognition Task (SCRT) Hinting Task	Social cognition (i.e., social perception) was positively associated with participant's perceptions of their ability to complete social functioning tasks (i.e., independence‐competence), social cognition domains (i.e., social perception and emotion processing) were negatively associated with the successful completion of social functioning tasks (e.g., participation in prosocial and recreational activities). The data suggest that the relationship between social cognition and social functioning may be more complex than previously hypothesized. Ultimately, greater clarification of the nature of this relationship may help to inform the development and refinement of evidence‐based social cognition interventions for individuals with psychosis.

Abbreviations: CHR, clinical high risk; DSM‐3/4/5, *Diagnostic and Statistical Manual of Mental Disorders* third/fourth/fifth editions; FEP, first episode psychosis; HCs, healthy controls; ICD‐10, *International Classification of Diseases* 10th edition; SCID‐1/2/4/5, Structured Clinical Interview for DSM Disorders first/second/fourth/fifth editions; SSD, schizophrenia spectrum disorder; SZ, schizophrenia.

### 3.13 High Social Functioning Factors

Factors found to be associated with high social functioning in psychosis (see Table 5) included emotional awareness, acceptance of emotions, positive affect, cognitive reappraisal, positive performance beliefs and adaptive coping. Therefore, the factors associated with high or low social functioning could help to inform the development of social functioning interventions for psychosis.

**FIGURE 2:**
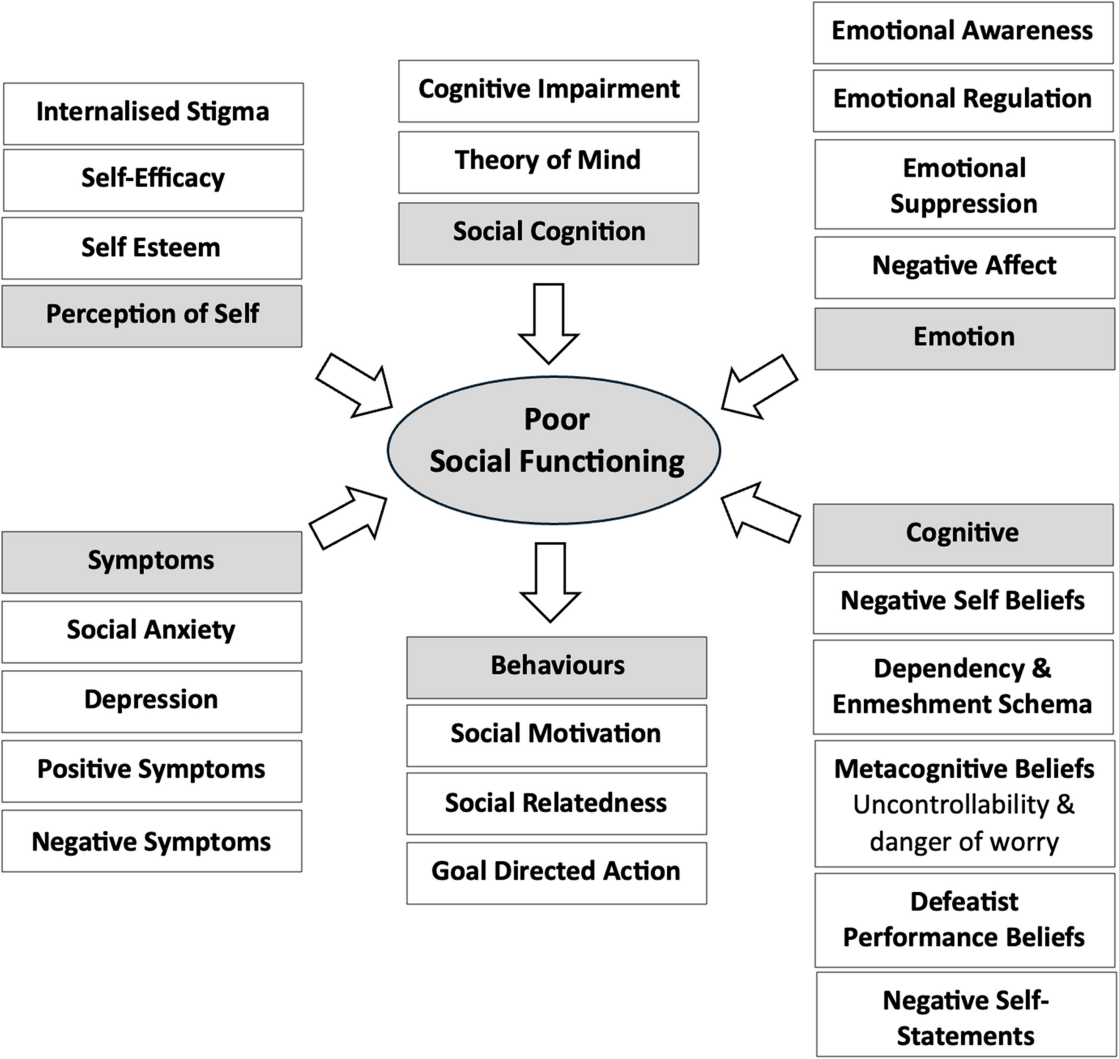
Negative factors contributing to poor social functioning in psychosis.

Based on the factors found to be associated with low social functioning (see Table 5), we have visually summarised the *negative factors contributing to poor social functioning in psychosis* (see Figure 2). The factors associated with poor social functioning in psychosis can be categorised under the domains of *self‐perception*, *symptoms*, *emotion*, *cognition*, *social cognition* and *behaviours*. These factors therefore highlight important psychosocial intervention targets in order to enhance social functioning in psychosis.

**FIGURE 3:**
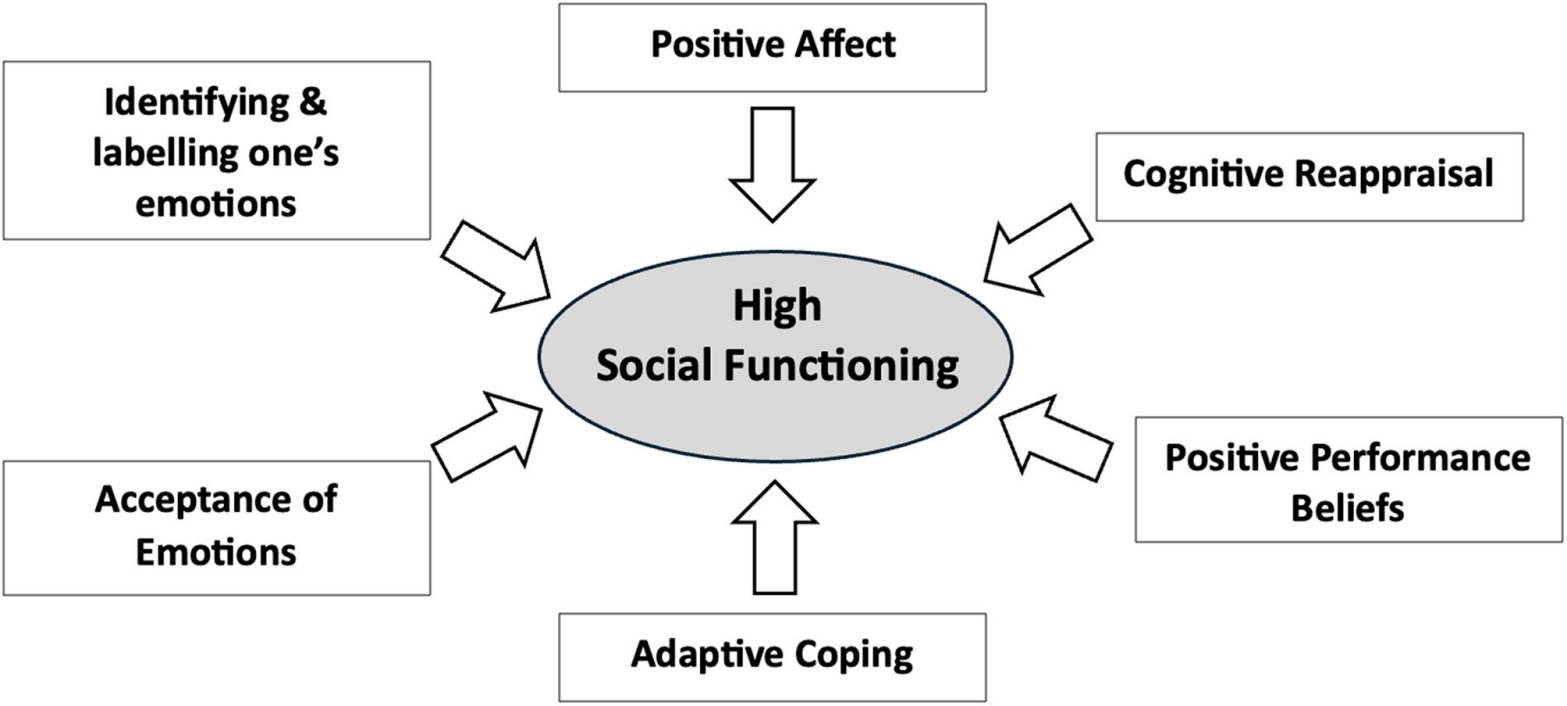
Positive factors contributing to social functioning in psychosis.

Based on the factors found to be associated with high social functioning (see Table 5), we have visually summarised the *positive factors contributing to social functioning in psychosis* (see Figure 3). The factors associated with high social functioning in psychosis include *positive affect*, *identifying and labelling one's emotions*, *acceptance of emotions* (as opposed to emotional avoidance), *cognitive reappraisal*, *positive performance beliefs* and *adaptive coping behaviours* such as active problem‐solving and the pursuit of goal‐directed activity. These factors therefore highlight potential intervention targets in CBTp to enhance social functioning in psychosis.

### 3.14 Cognitive Model of Social Functioning in Psychosis

**FIGURE 4:**
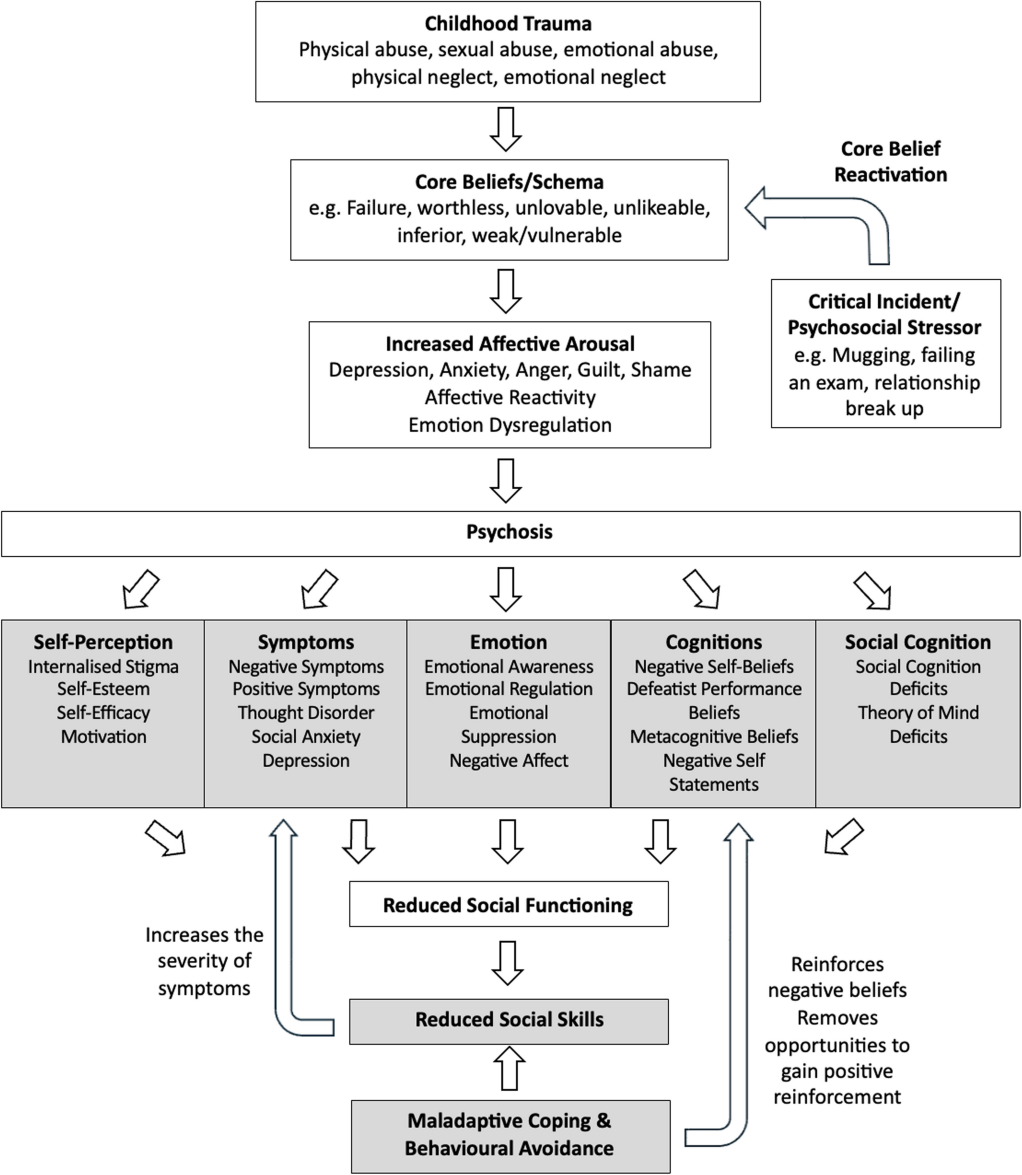
Cognitive model of social functioning in psychosis.

Based on the findings of the present review regarding the factors associated with high or low social functioning in psychosis and building on our previous published work (Jorovat et al. 2025; Georgiades et al. 2023), we propose the following *cognitive model of social functioning in psychosis* (see Figure 4). Our model proposes that childhood traumatic events such as physical, sexual, and emotional abuse, as well as physical and emotional neglect contribute to the formation of core beliefs such as ‘I am a failure, inferior, weak/vulnerable, worthless, and/or unlovable/unlikeable’ and others are ‘untrustworthy, dangerous, uncaring and/or unreliable’. A critical incident/psychosocial stressor (e.g., mugging, failing an exam, relationship break‐up) would serve to reactivate pre‐existing core beliefs, thereby inducing a state of high negative affect leading to the development of psychosis. The onset of psychosis could then impact the individual in terms of their *self‐perception, symptoms*, *emotions*, *cognitions* and *social cognition*, where one or more of these domains would contribute to reduced social functioning and subsequently to reduced *social skills*, which in turn would exacerbate the severity of symptoms. Additionally, the use of *maladaptive coping and behavioural avoidance* would reinforce negative self‐beliefs, as they remain unchallenged, and would reduce the opportunity to obtain positive rewards, thereby maintaining high levels of anxiety and depression.

This formulation model therefore highlights a number of possible interventive avenues to enhance social functioning in psychosis within each of the domains of *self‐perception*, *symptoms*, *emotions*, *cognitions*, *social cognition*, *maladaptive coping and behavioural avoidance* and *reduced social skills*. Clinicians can thus explore each of these domains collaboratively with their clients in order to devise a personalised list of factors impeding on that client's social functioning capabilities, which can then be explicit targets for intervention (see Table 6). For example, within the *self‐perception* domain, the clinician could address internalised stigma, low self‐esteem and self‐efficacy and can explore factors that may increase intrinsic motivation and goal‐directed activities. Within the *symptom* domain, clinicians could explore how symptoms of psychosis and/or mood impact on social functioning so strategies can then be employed to overcome symptom difficulties (e.g., by employing voice coping strategies, acting against the urge to avoid as in social anxiety and depression and increasing activities via behavioural activation for depression). Within the *emotion* domain, clinicians could explore emotional processes such as emotional avoidance/suppression, emotional regulation skills, one's coping with negative affect and the level of emotional awareness (i.e., ability to identify and recognise one's emotional state). The clinician could then support the client to enhance one's emotional awareness, foster emotional regulation skills, reduce emotional suppression and explore more helpful strategies for coping with negative affect. Moreover, for the *cognition* domain, clinicians could explicitly target negative self‐beliefs (‘I'm a failure, worthless, inferior’) and defeatist performance beliefs (‘I can't succeed, there's no point in trying, I can't compete with others’), metacognitive beliefs (‘I must dwell on negative thoughts, all my thoughts are dangerous and must be controlled’) and negative self‐statements (‘I'm not good enough, I can't do anything right’). For the *social cognition* domain, clinicians could help clients to think about the possible thoughts, feelings and intentions of others within social interactions to facilitate less threatening interpretations. Furthermore, exploring *maladaptive coping and behavioural* avoidance in response to each of these domains may reveal important additional intervention targets such as rumination, substance misuse and social withdrawal. Clinicians would therefore seek to reduce *maladaptive coping and behavioural avoidance* and increase more adaptive behaviours (i.e., problem‐solving, exercise, spending time with others). Moreover, exploring *social skills*, such as use of eye contact, body language, assertiveness, expressiveness and responsiveness in social interactions would highlight additional interpersonal intervention targets.

**TABLE 6:** Socratic questions exploring each social functioning domain and associated CBT strategies.

**Self‐perception** *How has your experience of psychosis impacted your view of yourself? (failure, inferior, weak/vulnerable, worthless, unlovable/unlikeable)—Work on strengthening positive core beliefs*. *Has your episode of psychosis affected your self‐esteem at all? In what way?—Employ cognitive restructuring for negative appraisals about self and develop more compassionate beliefs to hold instead, e.g., I experienced too much stress and developed psychosis, I am still a worthwhile and valuable human being*. *What about your ability to get tasks done?—Construct a behavioural hierarchy of exposure tasks to build activities in the client's week*. *How is your motivation currently? How does it compare to before your episode/before you came to our service?—Explore the client's values and life goals and break down goals into daily steps to achieve them*. **Symptoms** *Do the voices ever make it difficult for you to go outside and/or interact with others? If so, what happens?—Explore beliefs about the voice and the need to comply, conduct behavioural experiments to challenge negative predictions about the voice*. *Does the suspiciousness (delusional belief) impact on your ability to interact with others or do things you want to do?—Conduct behavioural experiments to test the likelihood of the feared prediction coming true*. *Do you ever feel anxious socially? Do you tend to avoid people?—Address avoidance and conduct behavioural experiments to re‐engage in social tasks in a graded manner and practise external focused attention rather than internal focused attention*. *Do you ever feel low in mood? Does that stop you from doing anything you want to be doing? (socialising/work/study)—Address behavioural avoidance, acting against the urge to avoid, building weekly activities, engaging with activities despite feelings of low mood as engagement in activities will enhance mood over time*. **Emotion** *Can you identify what emotions you are feeling?—Discuss emotions and how to identify them in the body*. *Can you identify what emotions someone else is feeling?—Discuss recognising emotions in others and the value of this for social connection, provide psychoeducation about emotions*. *When you feel stressed or overwhelmed emotionally, how do you cope, what do you do? (Socially withdraw/isolate/act confrontationally/irritably/use alcohol/substances/overthinking)—Explore unhelpful coping with emotions*. *What helps when you are emotionally overwhelmed/what does not help?—Explore helpful and unhelpful emotional coping, encourage more helpful coping and the reduction/cessation of unhelpful coping*. *How do you cope with negative emotions (sadness/anger/anxiety/guilt/shame)?—Explore how negative affect is responded to (negative affect is avoided vs. mindfully acknowledged and processed/used as a source of information)*. *Do you tend to feel these difficult emotions, or do you try to avoid them?—Discuss advantages and disadvantages of emotional avoidance, may be helpful in the short term but may increase psychosis emergence in the long term, need to process emotions rather than suppress them*. *What makes you feel positive emotions?—Explore thoughts and behaviours that enhance positive affect*. **Cognition** *How do you tend to view yourself? (failure, inferior, weak/vulnerable, worthless, unlovable/unlikeable)—Work on strengthening positive core beliefs*. *Do you feel confident in your ability to complete tasks? If not, what do you think will happen?—Encourage active problem‐solving, explore the presence of any negative/defeatist performance beliefs and use cognitive restructuring and behavioural experiments to test these negative predictions and to build confidence in one's sense of self‐efficacy/enhance positive performance beliefs*. *How do you tend to view your thoughts, e.g., as important, significant, dangerous, uncontrollable, must be dwelled on?—Explore metacognitive beliefs and use cognitive restructuring/behavioural experiments to test the validity of these beliefs/how helpful they are to hold*. *Do you make any negative self‐statements about yourself, e.g., regarding failure/competence/inferiority, etc.?—Use cognitive restructuring and replace these negative self‐statements with more positive compassionate self‐statements to hold instead, e.g., I am growing as a person each day; I will try to be the best person I can be; I am worthwhile and valuable; I have experienced a lot of stress but that does not have to define who I am*. **Social cognition** *Can you think of a time when you misunderstood what someone was thinking or feeling? What happened?—Explore social misunderstandings, social cues and the client's perception of others' intentions and behaviours*. *Are there times when you feel unsure about how to react to what others are saying or doing, because you are unsure of what they are thinking or feeling?—Role‐play direct communication skills/assertiveness*. *Do you find it difficult to understand how your actions might affect other people's feelings?—Try out new ways of behaving and explore its impact on social interactions*. *Do you sometimes misunderstand someone's body language or facial expressions? Can you think of a time when that happened?—Use role‐play for managing social interactions/explore alternative ways of thinking and coping*. *Do you ever struggle to understand what people expect of you in social situations?—Encourage active problem‐solving and role‐play direct communication skills*.

Therefore, the *cognitive model of social functioning in psychosis* enables the development of a personalised and idiosyncratic formulation regarding social functioning difficulties in psychosis that would facilitate a tailored and targeted intervention within CBTp. None of the interventions within the scope of this review explicitly address the multiple factors found to be associated with high or low social functioning, which this model has integrated within a single framework. This model can be used as a stand‐alone intervention or can be integrated within existing interventions to promote functional recovery in psychosis. This could then be utilised within individual CBTp feasibility studies and subsequently within RCTs to determine its clinical utility.

### 3.15 Case Example: ‘John’

John grew up in a highly critical household where he was frequently told that he was ‘useless’ and ‘good for nothing’ by his parents (emotional abuse). He was a shy child and struggled to make friends at school, which led him to be physically bullied by his peers (physical abuse). These experiences led him to develop beliefs about himself as a ‘failure’ and others as ‘critical’ and ‘untrustworthy’ (core beliefs). He attended university but found it difficult to make friends and failed his first set of exams, so decided to drop out of university (critical incident) rather than retake his exams, as he did not think ‘there was any point in trying’ (defeatist performance beliefs). He applied for multiple jobs but was unsuccessful (critical incident). These events reinforced his belief that he was a ‘failure’ (core belief reactivation) leading him to experience intense feelings of sadness (increased affective arousal). He then began hearing an aggressive voice that sounded like his father telling him that he was ‘useless’ and would ‘amount to nothing’ (positive symptoms of psychosis, auditory hallucinations), which further lowered his mood (depression). He began to worry that he was becoming mentally unwell and thought that others would reject him if they knew he was hearing voices, so kept his symptoms to himself (internalised stigma). He bottled up his emotions (emotional suppression) and noticed that whenever he felt any sadness emerging (negative affect), he would drink alcohol to make him ‘forget all about it and sleep’ (maladaptive coping). He socially withdrew from people to avoid disclosing that he did not complete university and could not find a job (behavioural avoidance). The more he avoided people, the less confident he felt in his ability to interact with them (reduced social skills) and noticed that his voices got louder and more aggressive (increased symptom severity). He also walked down the street with his hoody up and his head down, avoided eye contact and spoke in a whisper to remain as ‘invisible as possible’ (behavioural avoidance). When he did have a conversation with someone, he thought that they were ‘judging him negatively’ for his lack of achievements and would know all about his failures (ToM deficits).

In therapy, his clinician would address each of the domains contributing to his reduced social functioning as follows:

- **Self‐perception**
  ○ *Internalised stigma:* Explore stigmatising beliefs and the situations in which self‐disclosure may be helpful or unhelpful (e.g., health professionals/people he can trust vs. strangers). Could carry out a behavioural experiment to ask what someone might think of someone who hears voices and whether it is as negative as he anticipates and, if it is negative, explore how he would cope
- **Symptoms**
  ○ *Positive symptoms of psychosis (auditory hallucinations):* Introduce voice coping strategies and psychoeducation about psychosis
  ○ *Depression:* Introduce behavioural activation to increase (pleasure and mastery) activities and structure in one's week
- **Emotion**
  ○ *Emotional suppression:* Explain that emotional suppression may make symptoms of psychosis worse and discuss the importance of expressing thoughts and feelings rather than bottling them up
  ○ *Negative affect:* Increase mindful awareness of difficult emotions (e.g., sadness, anxiety) and gradually increase tolerance by experiencing these emotions for increasingly longer durations in the absence of unhelpful coping (i.e., alcohol)
- **Cognitions**
  ○ *Negative self‐beliefs, e.g., failure:* Explore the evidence for and against those beliefs, advantages and disadvantages of believing those beliefs and of dwelling on them
  ○ *Defeatist performance beliefs:* Explore the client's values and encourage behaviours that align with those values, encourage engagement with meaningful activities despite having these unhelpful thoughts, develop compassionate self‐statements (e.g., ‘I can try my best and learn from my mistakes’)
- **Social cognition**
  ○ *ToM deficits:* Explore possible perspectives, intentions and emotions of others. Role‐play direct communication skills.
- **Maladaptive coping and behavioural avoidance**
  ○ *Social withdrawal:* Explore the advantages and disadvantages of behavioural avoidance and how to begin re‐engaging with important life areas (work/study, family/friends)
  ○ *Alcohol use:* Explore the advantages and disadvantages of alcohol consumption and replace with more adaptive behaviours (e.g., exercise, hobbies, connection with others)
- **Reduced social skills**
  ○ *Poor eye contact, posture, whispering:* Role‐play improving posture, making eye contact and not whispering and conduct behavioural experiments to see what type of reaction the client receives from others and whether his feared predictions come true (e.g., I will be rejected, shouted at, harmed)

## 4 Discussion

This systematic review sought to comprehensively summarise the existing literature regarding interventions explicitly designed to improve social functioning in psychosis, while also identifying the factors associated with high or low social functioning. All interventions examined were found to improve social functioning and reduced both positive and negative symptoms by targeting cognitive, emotional and behavioural processes.

### 4.1 Duration and Frequency

The duration and frequency of interventions that demonstrated effective outcomes included: physical exercise interventions for 1–2 h weekly, occurring for 10 weeks to 3 months; SST for 10–15 sessions; SRT for 12–16 sessions; online therapies for 10–12 sessions; psychosocial interventions for 12–16 sessions; metacognitive interventions for 12–16 sessions; and VR interventions for 10–15 sessions. Art interventions did not specify the exact session count or duration. Overall, the optimal therapy duration and frequency varied across interventions, with individual interventions requiring between 12 and 16 sessions and group interventions requiring between 8 and 12 sessions.

### 4.2 Individual Versus Group

Metacognitive (*n* = 2) and VR interventions (*n* = 6) employed individual sessions only. Physical exercise (*n* = 5), art (*n* = 3), OT (*n* = 3) and SST (*n* = 8) interventions favoured a group format. SRT (*n* = 2), online interventions (*n* = 4) and psychosocial interventions (*n* = 16) all involved a combination of individual and group sessions. Of these interventions, 19 were group‐based. Group formats may offer therapeutic benefits through peer interactions and shared learning, which may potentially explain the observed improvements in clinical symptoms (Lyons et al. 2021). However, group formats increase the difficulty in devising personalised formulations for individual group members due to the collective focus on skills acquisition. This therefore highlights an important gap in the social functioning literature, namely, the development of personalised formulations within one‐to‐one CBTp interventions that explicitly target the factors found to be associated with high or low social functioning in psychosis.

### 4.3 Factors Associated With High or Low Social Functioning

Regarding the factors associated with high or low social functioning in psychosis, the interventions reviewed varied regarding what they explicitly targeted. Physical exercise interventions involved aerobic and resistance training, which were specifically designed to improve physical health such as BMI and cardiorespiratory fitness. These interventions also involved group activities/role‐playing scenarios to enhance social engagement. Art interventions (drawing and music) targeted emotional awareness, suppression and avoidance. SRT targeted a sense of agency, hopelessness, feelings of stigma and negative beliefs about self and others. This intervention employed behavioural experiments, graded exposure and the active promotion of social activities. Metacognitive interventions improved emotional awareness, metacognitive beliefs and positive performance beliefs by encouraging clients to reflect on their emotions and thoughts through role‐playing and interactive exercises, while also fostering adaptive coping behaviours through goal setting. SST focused on improving social interaction performance, interpersonal skills and reducing social anxiety. VR interventions provided exposure to scenarios replicating previously avoided situations. Online interventions addressed social cognitive domains of affect perception (both visual and vocal), perception of social cues, ToM and self‐referential processing. Psychosocial interventions focused on improving emotional awareness, identifying and labelling one's emotions, acceptance of emotions, negative affect, motivation and social cognition, as well as reducing defeatist performance beliefs, low self‐efficacy beliefs, negative self‐statements, negative beliefs about self and others, hopelessness and feelings of stigma. According to the factors associated with low social functioning in psychosis (see Table 5), these psychosocial interventions did not explicitly target the following factors: emotional regulation, emotional suppression, dependency and enmeshment schemas, social relatedness, positive symptoms, depression and self‐esteem. These interventions also did not explicitly address the factors associated with high social functioning (see Table 5), namely cognitive reappraisal and positive affect. Therefore, integrating the components found to be associated with high or low social functioning could enhance social functioning interventions for psychosis.

### 4.4 Existing Evidence

Negative symptoms such as avolition and anhedonia contribute to social functioning deficits yet demonstrate a limited response to pharmacotherapy (Blanchard et al. 2011; Hunter and Barry 2012; Leucht et al. 2017). In contrast, cognitive remediation therapy (CRT) has been found to improve negative symptoms with moderate effect sizes (−0.30), which was maintained at follow‐up (Cella et al. 2017). Therefore, it is possible that improvements in negative symptoms via cognitive remediation would have an indirect positive effect on social functioning. Moreover, social cognition has been found to play a significant mediating role in the relationship between cognitive impairments and social functioning in psychosis (Uchino et al. 2023). Social cognitive abilities refer to an individual's mental processes concerned with perceiving, interpreting and processing social information (Green et al. 2008). Meta‐analytical evidence indicates a strong association between social cognition deficits and impaired social functioning in psychosis (Fett et al. 2011). Additionally, ToM, a component of social cognition (defined as an individual's ability to understand and interpret the beliefs, thoughts and intentions of others, allowing for the prediction and explanation of their behaviours, Premack and Woodruff 1978), has also been found to contribute to social functioning deficits (Roncone et al. 2002). This therefore implicates social cognition as a critical target for interventions. Conceptual disorganisation, cognitive impairments and ToM deficits can also significantly impact how individuals with psychosis interpret social events and others' intentions, often leading to misperceptions and social withdrawal (Dodell‐Feder et al. 2013; Green and Horan 2010; Pan et al. 2021). Indeed, conceptual disorganisation has been found to negatively impact how individuals with psychosis interpret others' behaviour, resulting in interpersonal conflicts and increased social avoidance (Pan et al. 2021). Additionally, cognitive impairments in psychosis, exacerbated by stress‐induced neuroinflammation, may impair emotional processing and social reasoning, leading to negative interpretations of social events resulting in social avoidance and reduced social functioning (Dunne et al. 2017). Avoidance of social situations reinforces negative self‐beliefs, as they remain unchallenged and removes opportunities to obtain positive rewards, thereby maintaining high levels of anxiety and depression. Furthermore, deficits in ToM can make social interactions challenging by heightening social stress and reducing social reward, resulting in social anhedonia and reduced social functioning (Dodell‐Feder et al. 2013). Collectively, these findings underscore the importance of cognitive impairments and ToM deficits in the development of negative interpretations of events and others' intentions, which in turn leads to misperceived threats and reduced social functioning in psychosis.

In terms of symptomatology, command hallucinations and auditory hallucinations have also been found to contribute to social avoidance and reduced social functioning. Individuals with psychosis experiencing command hallucinations and auditory hallucinations may feel compelled to avoid social interactions to protect against perceived threats made by the voices (Chaix et al. 2014; Varese et al. 2011). Social avoidance is driven by the sense of danger and mistrust, which further exacerbates social withdrawal and impairs social functioning (Farina et al. 2022). Therefore, addressing command/auditory hallucinations would also be clinically meaningful, as they can hinder an individual's ability to engage in and maintain their relationships, often resulting in social isolation and impaired functioning. According to the *cognitive model of positive symptoms of psychosis* (Garety et al. 2001) and the *Bio‐Psychosocial Model of Transition to Psychosis* (Georgiades et al. 2023), avoidance and social withdrawal, as well as psychosocial stress and interpersonal sensitivity, contribute to the onset and maintenance of psychosis symptoms. Avoidance behaviours prevent challenges to one's psychosis appraisals, while negative social environments increase negative schemas and emotional distress. According to the ‘Cognitive Model of Persecutory Delusions’ (Freeman et al. 2002), avoidance and social withdrawal reinforce delusional distress and conviction. Moreover, the *interpretation of intrusions model of psychosis* (Morrison 2001) emphasises the role of misinterpreted thoughts and avoidance in worsening psychosis symptoms and social functioning. Additionally, the ‘Integrative Cognitive Model of Internalized Stigma’ (Wood et al. 2017) highlights how internalised stigma, driven by societal stereotypes, leads to avoidance and social withdrawal, further impairing social functioning in psychosis. In addition to the negative influence of social withdrawal on psychosis, emerging evidence has been found for the role of core beliefs in psychosis symptom emergence (Jorovat et al. 2025). A recent systematic review and meta‐analysis found that compared to healthy controls, SZ and CHR scored significantly higher for negative self and negative other‐beliefs and significantly lower for positive self and positive other‐beliefs (Jorovat et al. 2025). The meta‐analytic findings revealed statistically significant large effects for negative self (*d* = 0.91, 95% CI [0.75, 1.07]) and negative other‐beliefs (*d* = 0.89, 95% CI [0.59, 1.20]) in SZ compared to controls on the Brief Core Schema Scale (BCSS) (Jorovat et al. 2025). These authors thus concluded that the explicit targeting of negative self and other‐beliefs, whilst also enhancing positive self‐beliefs would be clinically meaningful intervention targets in CBTp (Jorovat et al. 2025). Therefore, focusing on negative core beliefs such as failure and inferiority within social functioning interventions may lessen social avoidance and increase social engagement in psychosis.

### 4.5 Strengths and Limitations

This systematic review sought to provide a comprehensive summary of the existing literature regarding interventions explicitly designed to improve social functioning in psychosis, while also identifying the factors associated with high or low social functioning. Employing a mixed methods synthesis of quantitative and qualitative studies enabled a comprehensive synthesis of the extant findings regarding social functioning interventions in psychosis, which is a strength of the current review (Sandelowski et al. 2006). Another strength was the inclusion of studies employing validated measures of social functioning, which enabled a comparison of findings across studies and clinical populations. From these findings, we have proposed a novel cognitive model, namely, the *cognitive model of social functioning in psychosis* (see Figure 4), which can be used to devise personalised formulations to enhance social functioning in psychosis within CBTp practices. Drawing from the existing literature, we have visually summarised the *negative factors contributing to poor social functioning in psychosis* (see Figure 2) as well as *positive factors contributing to social functioning in psychosis* (see Figure 3) to elucidate the factors associated with low as well as high social functioning, respectively.

Some limitations of the present study pertain to the risk of not capturing relevant papers from the search outputs despite a manual search also being conducted. Additionally, the current review only included studies published in English and peer‐reviewed journals, possibly overlooking applicable research such as grey literature. Furthermore, most studies reviewed lacked long‐term follow‐up evaluations, which precludes examination of the sustainability of the learnt skills and the interventions' long‐term effects. Incorporating long‐term follow‐ups following a social functioning intervention would not only highlight which aspects of social functioning are indeed maintained/improved at follow‐up but which may need further targeting within the active intervention and/or via booster sessions. Follow‐ups may also reveal possible delayed effects of an intervention that short‐term assessments might not capture. The length of follow‐up is thus essential for evaluating whether the benefits observed are transient or long‐standing, highlighting the importance of longitudinal designs in future research of social functioning interventions. Many studies also lacked control groups and randomisations, limiting the ability to attribute social functioning improvements solely to the intervention. Moreover, the use of non‐validated or self‐reported measures in some studies raises concerns regarding the reliability of the outcomes (Baskaran et al. 2023; Restek‐Petrović et al. 2014). Additionally, while some participants, especially those with better social understanding, may benefit from online social interactions, others might struggle with the ambiguity of group‐based online environments (Alvarez‐Jimenez et al. 2021; Lal et al. 2023; Özer and Dişsiz 2024). This could potentially lead to unhealthy thought patterns and perceptual distortions in some individuals, resulting in negative responses to the intervention. Many studies also failed to control for pre‐intervention neuropsychological functioning, vital for metacognitive interventions. Severely cognitively impaired individuals may need cognitive remediation prior to engagement with social functioning interventions to fully benefit. Lastly, participants sociodemographic and clinical variations (diagnoses, presenting symptoms, duration of untreated psychosis, comorbidities) were not consistently collected, thereby making comparisons across studies difficult. Furthermore, no study to date has examined social functioning across the psychosis continuum, for example, in CHR, FEP and SZ, which would elucidate important social functioning related differences across groups.

### 4.6 Clinical Implications

The identification of factors associated with high or low social functioning has important clinical implications. The synthesis of *negative factors contributing to poor social functioning in psychosis* provides a framework for identifying important intervention targets in the domains of *self‐perception*, *symptoms*, *emotion*, *cognition*, *social cognition* and *behaviours*. These factors can therefore be applied in the development of novel social functioning interventions to address one's sense of self‐efficacy/self‐esteem and internalised stigma, symptoms impacting on social functioning (social anxiety, depression, positive and negative symptoms), emotional regulation/suppression and coping with negative affect, appraisals, negative self‐statements, negative performance beliefs, negative self‐beliefs, dependency and enmeshment schema, and meta‐cognitive beliefs, theory of mind deficits, behavioural avoidance and unhelpful coping, as well as increasing goal‐directed activities and social relatedness and enhancing inherent motivation in accordance with one's values. The synthesis of *positive factors contributing to social functioning in psychosis* further highlights a strengths‐based approach to enhancing social functioning in psychosis, namely, by increasing the following: emotional awareness, acceptance of emotions, positive affect, cognitive reappraisals, positive performance beliefs and adaptive coping. No social functioning interventions to date have explicitly incorporated both sets of positive and negative factors within their intervention protocols, which highlights an avenue for future research. Additionally, the development of the *cognitive model of social functioning in psychosis* enables the development of idiosyncratic case formulations regarding social functioning impairments, which would enhance current CBTp practices.

### 4.7 Future Research

Future research would benefit from examining the effectiveness of the *cognitive model of social functioning in psychosis* for individual case formulations within CBTp interventions across CHR, FEP and SZ. Moreover, a feasibility and acceptability study examining the use of this formulation within individualised CBTp interventions explicitly targeting the identified factors associated with high or low social functioning in psychosis would be clinically meaningful. There is also a notable lack of qualitative studies exploring the subjective experience of social functioning in CHR, FEP and SZ, which could potentially provide further insights and identify additional unaddressed factors impacting on social functioning in psychosis. Furthermore, future research would benefit from investigating the impact of comorbid conditions, substance abuse, personality disorders and neurodiversity on social functioning in individuals with psychosis (González‐Blanch et al. 2015; Porter et al. 2025; Simonsen et al. 2008). Additionally, the role of cultural factors in psychosis in regard to social functioning also warrants further investigation (Ghanem et al. 2023). An exploration of how these factors influence response to social functioning interventions would enable adaptations to be made, ultimately guiding the development of more inclusive and effective therapeutic interventions.

### 4.8 Conclusion

This systematic review sought to synthesise the existing evidence regarding interventions explicitly designed to improve social functioning in psychosis. The findings demonstrated that interventions such as physical exercise, art therapy, SRT, metacognition, SST, VR, online programmes and psychosocial interventions effectively improved social functioning and reduced both positive and negative symptoms. A number of factors were associated with high or low social functioning in psychosis, which highlights important intervention targets for novel social functioning interventions. The *cognitive model of social functioning in psychosis* could facilitate the development of personalised and idiosyncratic formulations and targeted interventions in CBTp to enhance social functioning in psychosis.

## Ethics Statement

The author has abided by the *Ethical Principles of Psychologists and Code of Conduct* as set out by the BABCP and BPS.

## Conflicts of Interest

The authors declare no conflicts of interest.

## Data Availability

Data sharing is not applicable to this article as no new data were created or analysed in this study.
